# Rapid Screening of Ellagitannins in Natural Sources via Targeted Reporter Ion Triggered Tandem Mass Spectrometry

**DOI:** 10.1038/s41598-018-27708-3

**Published:** 2018-07-10

**Authors:** Jeremiah J. Bowers, Harsha P. Gunawardena, Anaëlle Cornu, Ashwini S. Narvekar, Antoine Richieu, Denis Deffieux, Stéphane Quideau, Nishanth Tharayil

**Affiliations:** 1 Department of Plant and Environmental Sciences, Clemson University, Clemson, SC 29631 USA; 2 Janssen Research and Development, The Janssen Pharmaceutical Companies of Johnson and Johnson, Spring House, PA 19477 USA; 3 University Bordeaux, ISM (CNRS-UMR 5255), 351 cours de la Libération, 33405 Talence Cedex, France

## Abstract

Complex biomolecules present in their natural sources have been difficult to analyze using traditional analytical approaches. Ultrahigh-performance liquid chromatography (UHPLC-MS/MS) methods have the potential to enhance the discovery of a less well characterized and challenging class of biomolecules in plants, the ellagitannins. We present an approach that allows for the screening of ellagitannins by employing higher energy collision dissociation (HCD) to generate reporter ions for classification and collision-induced dissociation (CID) to generate unique fragmentation spectra for isomeric variants of previously unreported species. Ellagitannin anions efficiently form three characteristic reporter ions after HCD fragmentation that allows for the classification of unknown precursors that we call targeted reporter ion triggering (TRT). We demonstrate how a tandem HCD-CID experiment might be used to screen natural sources using UHPLC-MS/MS by application of 22 method conditions from which an optimized data-dependent acquisition (DDA) emerged. The method was verified not to yield false-positive results in complex plant matrices. We were able to identify 154 non-isomeric ellagitannins from strawberry leaves, which is 17 times higher than previously reported in the same matrix. The systematic inclusion of CID spectra for isomers of each species classified as an ellagitannin has never been possible before the development of this approach.

## Introduction

The quality and composition of nutraceuticals derived from fruits and vegetables has been placed under greater scrutiny in recent years in part due to the willingness of health-conscious consumers to spend more for higher quality agricultural products. In addition to bioactive compounds such as vitamins E, C, sterols and carotenoids, the polyphenolic compounds in plants offer various degrees of antioxidant, anticancer, antimicrobial, anti-inflammatory, and anti-neurodegenerative benefits^1–6^. One specific group of polyphenolic metabolites that is of a higher nutraceutical and ecological value and is widely distributed in higher plants are ellagitannins^7–13^. In brief, plants first synthesize the molecular precursors of ellagitannins by enzymatic conversion of dehydroshikimic acid into gallic acid, then galloylated glucose forms are generated along the biosynthetic pathway until neighboring galloyl groups undergo oxidative coupling to form the hexahydroxydiphenoyl (HHDP) group^14^. The HHDP group has been leveraged in quantitative methods to measure ellagitannin content since hydrolysis liberates hexahydroxydiphenic acid that rapidly lactonizes into ellagic acid, irrespective of the chemical identity of the ellagitannin species^15^. However, this approach does not provide much insight into the structure of individual ellagitannins, which is critical since the nutraceutical value of ellagitannins are regulated by their molecular identity. Ellagitannins are one of the most diverse groups of plant phenolics and their complexity presents a major hindrance to structural elucidation efforts^6,16,17^.

Ultrahigh-performance liquid chromatography coupled with tandem mass spectrometry (UHPLC-MS/MS) is one of the more efficient approaches to characterize plant metabolites, including phenolics, in complex extracts^18–22^. Mass spectrometry is a robust technique for many phenolics, but ellagitannins present significant and unique challenges to current analytical measurement techniques as different numbers of galloyl and hexahydroxydiphenoyl subunits are esterified with glucose, which complicates fragmentation spectra and often requires manual interpretation^23–25^. Although each species generates reproducible fragmentation spectra, many first-generation product ions vary by isomeric form and automation of proposed structures to match existing spectral libraries becomes challenging without nuclear magnetic resonance (NMR) to offer complimentary structural confirmation^26,27^. More recently, the number of studies focusing on compound-specific fragmentation using multiple reaction monitoring (MRM) methods on triple-quadrupole mass spectrometers (QqQ-MS) has increased^20–22,28–34^. Although targeted assays are effective, unbiased discovery focused exclusively on ellagitannins can be improved by incorporating compound-specific fragmentation for class association into new method designs. Focused large-scale discovery efforts to detect unknown ellagitannins have generally been unaddressed. This is the first attempt to rapidly screen ellagitannins and systematically catalogue unique fragmentation spectra of isomers for potential inclusion in spectral libraries.

Exponential increases in protein identification and reproducibility of peptide measurements in recent years is a direct consequence of substantial advances in mass spectrometer design^35–41^. The unique instrument architecture of a quadrupole-Orbitrap-ion trap platform (Tribrid Orbitrap Fusion) has enabled proteomic applications with new measurement capabilities where multiple dissociation modes have been used in tandem^42–45^. Firstly, using refined and synthesized ellagitannin standards (Fig. S1), we leverage the high resolution and accurate mass capabilities of the Orbitrap mass analyzer to classify precursors with specific product ions generated from higher energy collision dissociation (HCD) as ellagitannins^46–52^. Further, we utilized collision-induced dissociation (CID) to generate unique fragmentation spectra of isomeric variants to differentiate between isomeric forms. Determination of constitutional or stereochemical isomerism was beyond the scope of this work and all isobaric species classified as ellagitannins with steric differences that resulted in unique CID fragmentation kinetics were retained under the label, isomer, although potential changes in conformational isomerism in the gas-phase cannot be addressed at this time. We demonstrate how a tandem HCD-CID experiment might be used to screen natural sources for ellagitannins using UHPLC-MS/MS by application of 22 method conditions from which an optimized data-dependent acquisition (DDA) that classified 154 non-isomeric ellagitannins emerged.

## Results

### Fragmentation of Ellagitannins

**Figure 1:**
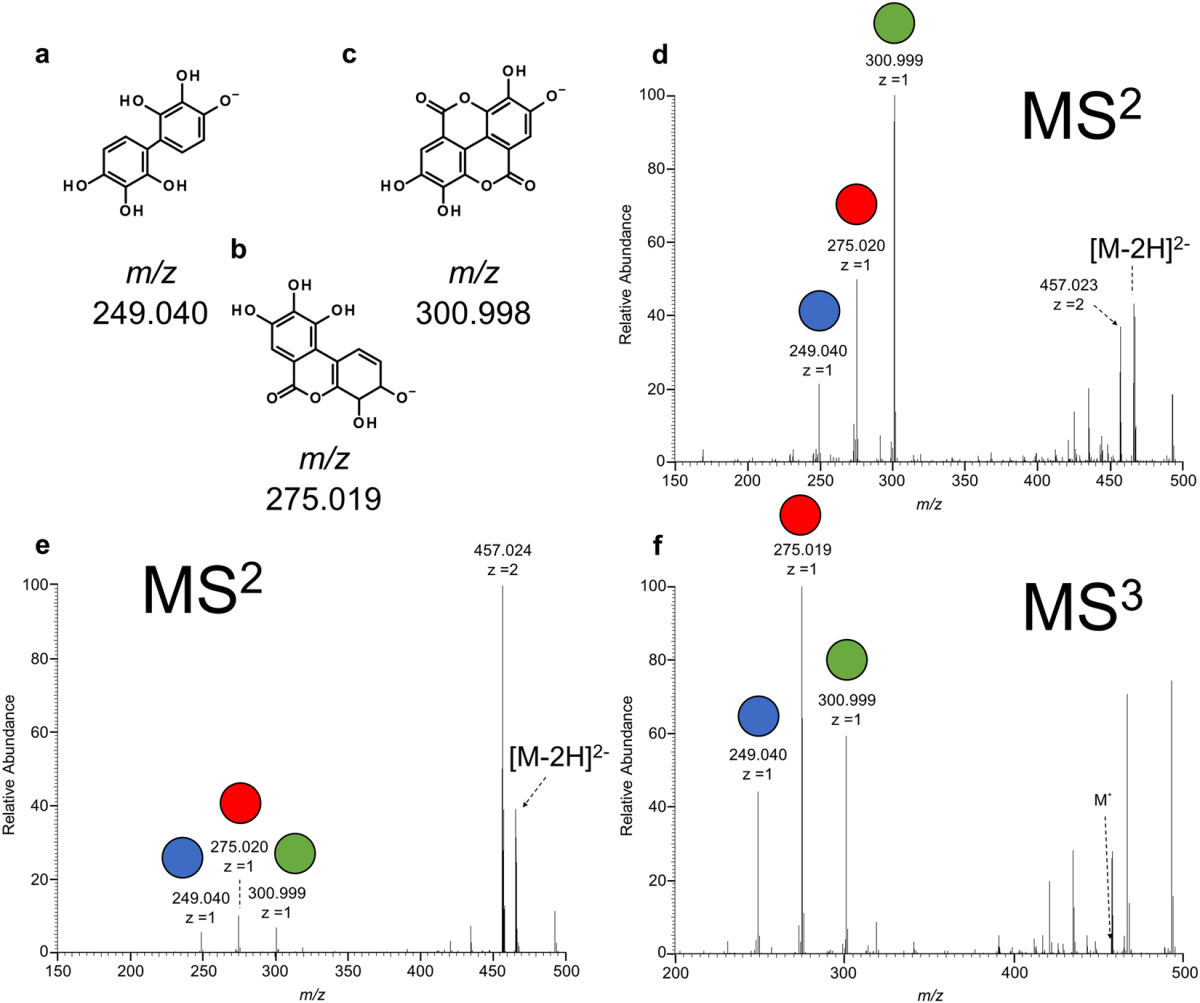
Characteristic reporter ions used to classify precursors as ellagitannins: (**a**) 2,2**′**,3,3**′**,4,4**′**-hexahydroxybiphenyl, (**b**) 3,4,8,9,10-pentahydroxydibenzo[b,d]pyran-6-one, (**c**) ellagic acid; CID MS^2^ spectra for: (**d**) castalagin, (**e**) vescalagin, and (**f**) the CID MS^3^ spectrum of the isolated 457 product ion from vescalagin. Blue, red, and green dot graphics above the 249, 275, and 301 reporter ions were added for improved contrast of the relative intensities in product ion spectra.

The tandem HCD-CID screen employed multiple modes of fragmentation to minimize the number of discrete experiments required to classify precursors and then generate fragmentation spectra for isomeric variants. Precursors were first subjected to HCD to generate characteristic product ions specific to ellagitannins (Fig. 1a–c) for classification without the need for sequential fragmentation inherent within traditional ion trap type CID MS^n^ approaches. This was followed by CID as it was better suited to generate unique fragmentation spectra of any isomers observed given the inherent specificity of the method since only first-generation product ions were formed^53–57^. These two conditions formed the basis of the proposed tandem HCD-CID screen designed to detect ellagitannins and acquire fragmentation spectra that could be used to develop annotated spectral libraries.

Infused standards were first subjected to CID to illustrate the disadvantage of selective excitation of precursor ions to classify unknown compounds as ellagitannins. Although fragmentation of intact castalagin anions provided prominent 249/275/301 reporter ions (Fig. 1d), most of the other standards did not produce significant amounts of these reporters (Figs 1e and S2) and required subsequent MS^3^ of either 457 (Figs 1f and S3) or 487 and MS^4^ of 465 (Fig. S4) to generate sufficient quantities of 249/275/301 ions. Tabular summaries of these results (Tables S1–S5) and MS^2^ spectra of lower charge states (Fig. S5) are available in the Supplemental Information. Given that precursor classification was driven by the detection of these reporter ions, an approach to maximize the abundance of these reporters within MS^2^ spectra of any ellagitannin was prudent to maximize method sensitivity.

Although in-source fragmentation has been employed previously for classification of ellagitannins based on observation of the ellagic acid 301 ion, that approach was less applicable since the isolation of precursor ions before fragmentation was found to be a more effective approach to properly associate unknown candidate species with the appearance of reporter ions given the potential coelution of different ellagitannins^32^. Modification of the linear ion trap (LIT) to maximize the formation of 249/275/301 ion populations in CID MS^2^ spectra through custom firmware was found to be impractal^58–60^. In contrast, HCD generated extensive fragmentation beyond the isolated precursor in MS^2^ spectra which maximized the accrual of all three reporter ions without any hardware or software modifications. Thus, discrete HCD fragmentation energies were applied to the [M-2H]^2−^ of each infused ellagitannin (Fig. S6) and the abundance of 249/275/301 reporter ions was used to create plots (Fig. S7) to visualize the optimal normalized collision energy (NCE) for each standard. Doubly charged anions were initially selected given the relatively low abundance of triply or singly charged precursors available (Fig. S8) under chromatographically relevant conditions. No significant difference in the formation of reporter ions was observed for species with triply charged anions (Fig. S9) and chromatographic conditions were not altered to improve the ionization of triply charged anions in later UHPLC-MS/MS experiments, as higher pH conditions adversely affected dianion ionization efficiencies.

Most ellagitannin species formed 301 more readily than 275 and 249, but fragmentation of grandinin and roburin E resulted in larger 249 product ion populations than 275 or 301. To minimize false-positive identifications, all three 249/275/301 ions were required to be present and within the top 10 most abundant peaks in each HCD spectrum to classify an unknown precursor candidate as an ellagitannin under targeted 3 reporter ion triggering (T-3RT). To optimize this assignment, the next step was to determine what NCE values would generate the largest cumulative ion populations of all three reporter ions. Sums of all three reporter ion abundances at each NCE value was normalized to the greatest total for each ellagitannin (Table S6) and no single NCE was observed to generate the largest total populations of 249/275/301 reporter ions. Although values of 25, 30, 35, and 40 each had maximums for certain species, others had significantly reduced values. An NCE of 35 or 40 might be adequate but the available stepped collision energy option for HCD allowed for the partitioning of precursor ions to be fragmented at three different collision energies and combined into a single scan.

Theoretical stepped NCE results were calculated (Table S7) from the empirical data (Table S6) to estimate that 40 +/− 10 was likely to provide the maximum generation of reporter ions for standards and this motivated acquisition of stepped energies of +/− 5 within the narrower 25–45 NCE range that appear in Table 1. The mean and standard deviation of the response of the standard set of ellagitannins to specific HCD conditions showed that 40 +/− 5 provided a slightly higher mean of 98.06 and a lower standard deviation of 1.87 than corresponding values for 35 +/− 5. Although 40 +/− 10 was a maximum for intervals of +/−10 (Table S8), the smaller mean of 88.53 and larger standard deviation of 4.29 indicated that the smaller interval was better. Using the responses from these ellagitannins as a basis, 40 +/− 5 was selected as the HCD setting to be used when screening to classify precursors as ellagitannins.

**Table 1:** Normalized Sums of 249/275/301 Intensities for NCEs Stepped by 5.

Standard Compound	25 +/− 5	30 +/− 5	35 +/− 5	**40 +/− 5**	45 +/− 5
castalagin	61.4	88.5	100	**95.4**	84.2
vescalagin	71.3	94.7	100	**98.6**	84.8
acutissimin A	45.7	72.7	100	**96.2**	97.5
epiacutissimin A	46.4	78.5	100	**96.8**	95.5
roburin A	50.5	81.2	100	**99.3**	95.2
roburin D	48.9	71.9	86	**100**	98.5
roburin B/C	62.7	89	100	**99.9**	90.6
grandinin	68.8	90	100	**96.3**	83.2
roburin E	63.1	79.9	94.2	**100**	77
$\bar{{\rm{x}}}$	57.64	82.93	97.8	**98.06**	89.61
σ	9.85	8.02	4.82	**1.87**	7.59

Although HCD performed better for reporter ion generation, fragmentation spectra generated by CID would be better for inclusion in spectral libraries. Even though a lower, non-stepped HCD collision energy could possibly provide comparable and consistent fragmentation of precursors classified as ellagitannins to that of CID, it would be more likely that acquired spectra would contain non-trivial and uncertain amounts of second and third-generation product ions as the exact threshold energies would be unknown^61^. An NCE of 20 was previously used to obtain pseudo-CID spectra for ellagitannins, but even relatively mild HCD conditions may result in different degrees of secondary fragmentation, as evidenced by changes in relative abundance of common fragments depending on molecular structure^62^.

Comparison of HCD and CID spectra for representative standard ellagitannins that produce different major first-generation product ions (Fig. S10) show that the ambiguity created by HCD could increase the difficulty of assignments within spectral library searches. Although grandinin and acutissimin A have different major first-generation product ions, 487 and 457 respectively, the most abundant second and third-generation product ions are the same 249/275/301 reporter ions. These reporter ions are multi-generational and their intensities would no longer accurately be associated with specific product ion generations if spectral libraries were comprised of pseudo-CID spectra from even mild HCD conditions^63^. Standard normalized collision energies for CID (Tables S1 and S2) ranged from 15–25 and 30 was chosen to ensure complete fragmentation while minimizing the possibility that smaller species would be ejected from the LIT.

### Analyzer Optimization

Specific MS and CID analyzer settings that were selected when applying an HCD-CID screen were derived from iterations of select instrumental settings and filters available in the Supporting Information within a similarly titled section. Ellagitannin standards were subjected to an HCD-CID screen during analyzer optimization that included a MIPS filter, an intensity filter of 1e5, and a charge state filter that only allowed dianions to be subjected to HCD.

### Mass Trigger Validation

Control experiments are provided in more detail in the Supporting Information. In brief, the response of the standard basis set showed that 2.5 ng of material was the minimum amount required to anticipate full peak shapes for species classified as ellagitannins. TRT conditions were altered to compare the differences of requiring all three reporter ions in T-3RT and variants that allowed any two reporter ions (T-2RT) or any reporter ion (T-1RT) to be observed in the top 10 most abundant peaks in an HCD spectrum to result in CID acquisition. Leaf extracts of Palmer amaranth (*Amaranthus palmeri*) that had no ellagitannin or HHDP derivatives was chosen to test for false positive CID events under each TRT condition. This extract provided over 280 precursor candidates to serve as potential false positive targets and although amaranth had no ellagitannins, it contained an abundance of quercetin-glycosides including quercetrin, isoquercitrin, and rutin. Major fragment ions of rutin and isoquercitrin included 300.029 and 301.037 (Fig. S38) which are close to the ellagic acid trigger ion of 300.998 that would have generated a false positive result without high resolution mass spectrometry (HRMS). By employing both HRMS and multiple trigger ions we were able to avoid false positive identification in complex plant extracts, which attests to the robustness of the optimized method. An additional advantage of HRMS over QqQ instrumentation is that MS^3^ of the 301 ion is unnecessary to confirm classification which allows more time for the instrument to scan for additional ellagitannin candidates without increasing the false positive rate. Further, a mixture of the ellagitannin standards was spiked to the amaranth extract to estimate false negative classification. No false negatives occurred under any TRT condition, but false positive events were observed under T-1RT which resulted in the acquisition of CID data in the absence of ellagitannin content. Strawberry analysis omitted T-1RT analysis given the false positives observed in amaranth.

### Filter Optimization

The Rosaceae family has been found to be higher in ellagitannin content than other fruits and vegetables and was chosen to test the tandem HCD-CID screen given the availability of existing reports available for comparison^62,64–67^. The high concentration of ellagitannins present in strawberry leaves provided an opportunity to examine the outcome of applying filters that directly affected which precursor ions were subjected to HCD and subsequently classified. Various filter combinations were combined to create the screens described in the Methods section of this report. Complete lists of ions classified as ellagitannins under each screen condition for both T-3RT (Tables S9–S19) and T-2RT (Tables S20–S30) are available in the Supporting Information.

The most pertinent results were condensed for T-3RT (Table S31) and T-2RT (Table 2) and show that the latter provided greater numbers of unique species classified. The cumulative values for the condensed tables differ from the complete lists in the Supporting Information as they do not include duplicates of species that were observed in multiple charge states or whose C^13^ isotope peak was not properly excluded by the MIPS filter to more accurately reflect the number of unique precursors classified using a specific screen. Estimation of the degree to which in-source fragmentation of larger unknown precursors may have contributed to these unique counts would require direct examination of larger standards in the 3-4 kDa range which are unavailable. Given that none of the larger ~2 kDa standards showed evidence of in-source fragmentation (Fig. S8), it seemed reasonable to infer that potential in-source fragmentation of larger ellagitannins did not significantly contribute to the summary values in the condensed tables. Additionally, though strawberry leaf extract had potential interferents such as quercetin glycosides present with along native ellagitannins this did not influence the robustness of the method in correctly identifying ellagitannins. The reason for this is that the optimized method utilizes both HRMS to select characteristic fragment ions specific to ellagitannins and then requires multiple reporter ions to be present before classifying a precursor which minimizes the possibility of false positive classification.

**Table 2:** Precursor ions present in strawberry leaves that met all criteria to be classified as ellagitannins under conditions [I–XI] using T-2RT.

T-2RT	I.	II.	III.	IV.	V.	VI.	VII.	VIII.	IX.	X	XI.
Anions	20	25	0	47	41	38	**50**	0	23	24	0
<5e5 Intensity	9	16	0	**45**	36	32	44	0	17	16	0
>5e5 Intensity	**11**	9	0	2	5	6	6	0	6	8	0
<900 Da	15	18	0	31	28	24	**33**	0	15	16	0
<1100 Da	20	24	0	46	40	37	**49**	0	23	24	0
>1100 Da	0	1	0	1	1	1	1	0	0	0	0
Dianions	45	0	97	124	109	100	0	**134**	51	0	52
<5e5 Intensity	22	0	80	115	99	84	0	**126**	33	0	33
>5e5 Intensity	**23**	0	17	9	10	16	0	8	18	0	19
<900 Da	3	0	**7**	6	**7**	**7**	0	**7**	2	0	2
<1100 Da	18	0	39	**45**	39	38	0	**45**	20	0	20
>1100 Da	27	0	58	79	70	62	0	**89**	31	0	32
Total	65	25	97	**171**	150	138	50	134	74	24	52
< 5e5 Intensity	31	16	80	**160**	135	116	44	126	50	16	33
>5e5 Intensity	**34**	9	17	11	15	22	6	8	24	8	19
Unique Mass	58	25	97	**154**	134	122	50	134	67	24	52

Screen descriptions defined in the methods section include: I. (Intensity), II. (Anion), III. (Dianion), IV. (DE 12 s), V. (DE 3 s), VI. (DE 3 s, Apex), VII. (−1, DE 3 s), VIII. (−2, DE 3 s), IX. (DE 3 s, 5e5), X. (−1, DE 3 s, 5e5), XI. (−2, DE 3 s, 5e5).

The smallest ellagitannin, 2,3-hexahydroxydiphenoylglucose, is defined from the condensation of single HHDP and glucose subunits (482.070 Da) but whether to include species with molecular weights lower than this when reporting ellagitannins has not yet been standardized^62,68^. A targeted mass exclusion list was initially considered to ensure no singly charged *m/z* below 481.062 would be included for precursor selection, but was not added to the method parameters given general interest and precedent set by recent studies^62,69–72^. Species below 482.070 Da were omitted from T-2RT enumeration (Table 2) to accurately report ellagitannin summary values in addition to the aforementioned adjustments for charge state and isotope duplicates. Initial transcription efforts from Freestyle allowed for screens [III] and [IV] to be used as a comparison when parameters of the Proteome Discoverer workflow were modified to the final form reported in the Supporting Information. An assessment of the most significant parameters within the Proteome Discoverer workflow that affected proper grouping of isobaric species is also available in the Supporting Information.

**Figure 2:**
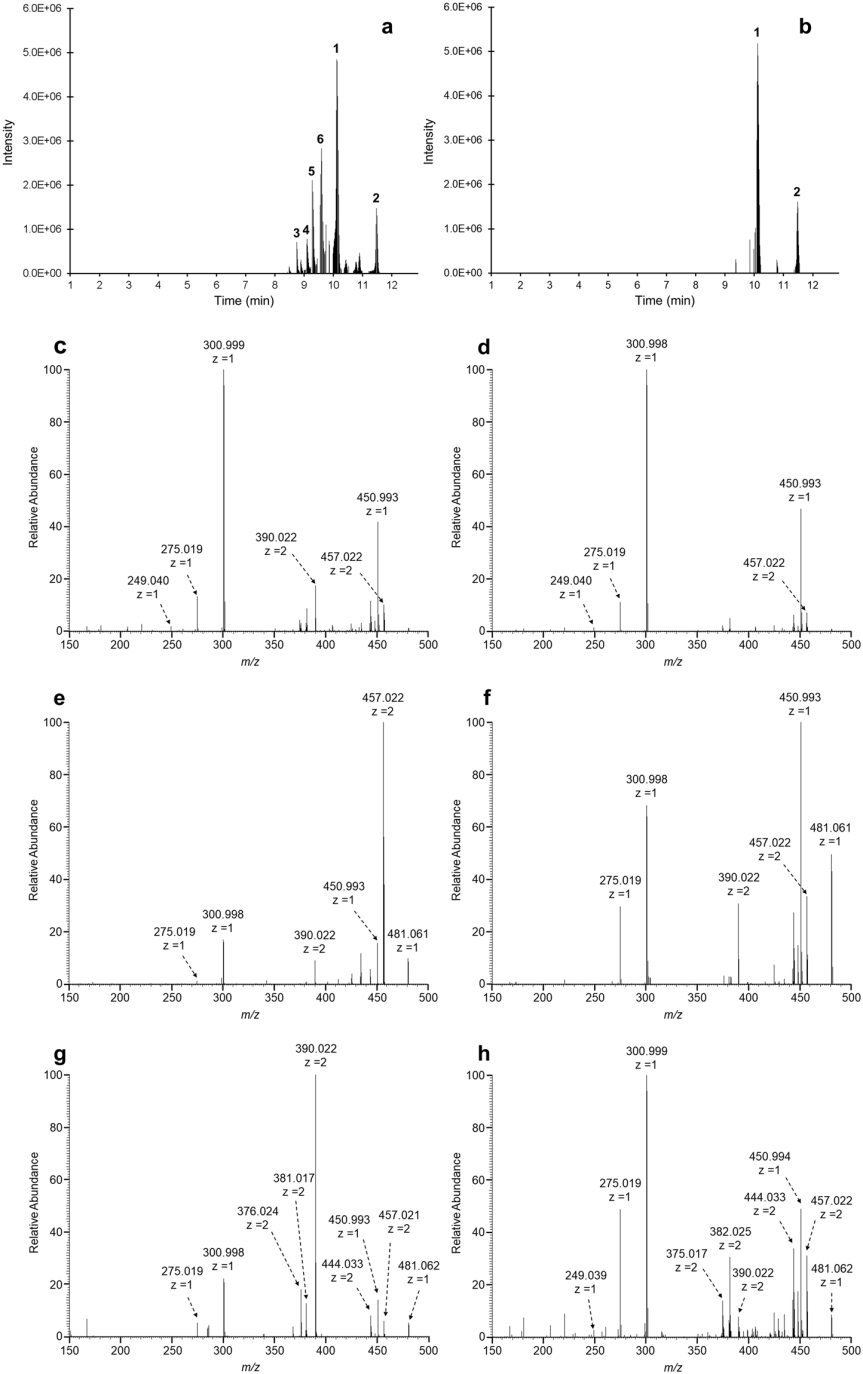
Elution profile of screen [III] for the isobaric 466.029 species present in strawberry under different TRT conditions: (**a**) T-2RT, (**b**) T-3RT; CID spectra of the 466.029 species acquired using screen [III] under T-2RT: (**c**) isobar ‘1’, (**d**) isobar ‘2’, (**e**) isobar ‘3’, (**f**) isobar ‘4’, (**g**) isobar ‘5’, (**h**) isobar ‘6’. Isobars with abundances greater than 5e5 observed with T-3RT and T-2RT at 10.13 min and 11.49 min were labeled 1 and 2 while those observed only under T-2RT at 8.75 min, 9.10 min, 9.29 min, and 9.59 min were labeled 3, 4, 5, and 6 respectively.

A previously reported species isobaric with the vescalagin (3.81 min) and castalagin (5.48 min) standards (Fig. S27) in strawberry leaf extract allowed for direct comparisons of how different TRT conditions affected which isomers were observed^66^. Examination of the [M-2H]^2-^ 466.029 *m/z* species acquired under all method variants except those with an anion charge state filter for both T-3RT (Fig. S39) and T-2RT (Fig. S40) conditions showed that more isomeric forms were observed with T-2RT given low levels of a single reporter ion, 249, prevented many isomers from being classified under T-3RT. Tentative structures of significant ions (Fig. S41) were prepared assuming even electron configurations for convenience, but proposal of gas-phase fragmentation mechanisms was beyond the scope of this work given the need to exclude stable radical dianions from consideration. Screen [III] was selected to illustrate the advantages of T-2RT over T-3RT (Fig. 2a,b) since manual selection of isomers by elution profile was easier given the absence of an automated pipeline to perform the same task for screens that utilized dynamic exclusion (DE). Isomers were readily differentiated by averaged CID spectra (Fig. 2c–h), but an automated process to extract, combine, and average spectra to build a spectral library of ellagitannins would be a required before product ion structures and associated gas-phase fragmentation mechanisms could be proposed on timescales common in other omics fields.

Ellagitannins from recent studies were tabulated (Table S32) and the 154 non-isomeric ellagitannins (Table S33) observed under T-2RT [IV] had 9 of 11 potential matches from species reported in strawberry leaves^32,62,64,72–74^. Two species that had masses above 2400 Da (Table S33) were not observed since this was above our set mass range. Expanding to include reports that utilized material other than strawberry leaves (Table S34) resulted in 21 tentative identifications. Although a subset of previous tentative labels includes multimers (Table S33), manual examination of CID spectra indicates that a systematic review of assignments based on fragmentation spectra will be required in future studies to confirm non-covalent associations.

## Discussion

Given the increased interest in biosourced commodities, there is a need for improved discovery methods using advanced LC-MS platform designs. We defined a new mode of operation, targeted reporter ion triggering (TRT) to classify one specific group of polyphenolic secondary metabolites in plants, the ellagitannins. The T-2RT condition requiring the observation of any two reporter ions to be observed was sufficient to ensure no incorrect classifications in the amaranth control and resulted in more ellagitannins and isomers than T-3RT in strawberry leaf extract. Despite their occurrence along with potential interferents such as quercetin glycosides, the ellagitannins in the strawberry leaf extract were correctly classified due to the utilization of HRMS and multiple reporter ions. Specific stepped HCD NCE conditions of 40 +/− 5 allowed for the maximum generation of 249/275/301 reporter ions used to label precursors as ellagitannins and an NCE value of 30 for CID was sufficient for the generation of fragmentation spectra of isomers. Leveraging the high resolution and accurate mass capacities of the Orbitrap with a tandem HCD-CID experiment resulted in an optimized method that detected 154 non-isomeric species in a single data dependent acquisition (DDA). Comparison with recent literature data showed that only 9 non-isomeric ellagitannins in strawberry leaves were provided tentative identifications from previous studies^64,73^. Based on this optimized method we were able to putatively identify 154 non-isomeric ellagitannins from strawberry leaves, which is 17 times higher than the number of ellagitannins reported in the same matrix. Broadening the search to include ellagitannins from any source increased this value to 21 tentative matches. The potential of comprehensive and systematic inclusion of CID spectra for the isomers of each unique species has never been possible before development of this TRT approach.

The tandem HCD-CID method presented is well positioned to be applied across other classes of natural products, where HCD derived reporter ions can serve as triggers for screening a compound class, although the 249/275/301 reporter ions are specific to ellagitannins and not applicable to other compound classes. The paucity of annotated structures of plant-based natural products such as ellagitannins makes accelerated structural assignment of CID spectra challenging^32,62,64,73,74^. In this regard, CID spectral libraries of compounds would first need to be created with existing tentative structure identifications most commonly found in tabulated lists which currently include a few hundred non-isomeric ellagitannins^14,32,62,64,74^. Complementary NMR data could then be acquired with preparatory UHPLC methods to systematically confirm tentative structural assignments. Hybrid search of MS spectral libraries structurally validated by NMR would drastically decrease the effort required to identify unknown ellagitannins^75^. We envision that our TRT method presents opportunities to rapidly generate CID spectral libraries and enable the adoption of quantitative omics workflows for plant natural product experiments. This method enabled the rapid classification of unknown precursors while still retaining a comprehensive and facile systematic inclusion of fragmentation spectra for isomers to support the development of annotated spectral libraries for a previously inaccessible class of compounds in natural sources.

## Methods

### Chemicals and Reagents

Castalagin, vescalagin, roburin A, roburin D, roburin E, grandinin, and a 1:1 mixture of roburin B and roburin C were extracted and purified from oak heartwood, while acutissimin A and epiacutissimin A were obtained by hemisynthesis using vescalagin and catechin^50–52^. Chemical structures of these ellagitannins standards are presented in Fig. S1. Optima UHPLC-MS grade acetonitrile and water, as well as Optima LC/MS grade methanol and formic acid, were purchased from Fischer Chemical; HPLC grade chloroform was obtained from Fischer Scientific.

### Strawberry Leaf Extraction

Description of the sample preparation procedure can be found in the Supporting Information.

### Instrumentation

All analyses were performed using an Ultimate 3000 HPLC (Thermo Scientific, Waltham, MA, USA) coupled to an Orbitrap Fusion (Thermo Scientific) Tribrid mass spectrometer equipped with an electrospray ion source using tune application software 2.1.1565.18 and Xcalibur 4.0.27.13.

### Infusion-MS/MS Analysis

Description of instrument parameters utilized for infusion of standard compounds can be found in the Supporting Information.

### UPLC-MS/MS Analysis

All samples subjected to LC-MS/MS analysis were separated on a Waters (Waters Corp., Milford, MA, USA) Acquity UPLC HSS T3 (150 × 2.1 mm, 1.8 µm) column at 30 ^o^C. The following gradient program utilizing water with 0.1% formic acid as mobile phase A and acetonitrile as mobile phase B was employed: 0 min, 10% B; 2 min, 10% B; 8 min, 30% B; 12.5 min, 60% B; followed by a 3-minute washing step at 90% B and a subsequent re-equilibration for 7 min at 10% B. The flow rate was set to 0.22 mL/min and the injection volume chosen was 2 µL. The mass spectrometer was operated in negative ionization mode with a data dependent MS^2^ HCD-CID method. The interface conditions were as follows: emitter voltage, −2600 V; vaporizer temperature, 325 ^o^C; ion transfer tube, 325 ^o^C; sheath gas, 55 (arb); aux gas, 10 (arb); and sweep gas, 1 (arb).

### Method Settings

Internal mass spectrometer settings utilized for MS scans unless stated otherwise were as follows: mass range 150 *m/z* to 1200 *m/z*; RF lens, 60%; AGC target, 4e5; maximum injection time, 50 ms; and 1 µscan in profile mode at 50 K resolution on the Orbitrap mass analyzer. The method then sequentially included a series of filters prior to any HCD MS^2^ events. A monoisotopic peak selection filter was included and set as peptide for all methods as this setting functioned as well as others available. An intensity filter of 1e5 was utilized for all methods unless stated otherwise. An optional charge state filter was included for some methods to select precursor charge states of either 1, 2, or 1 & 2. An optional dynamic exclusion (DE) filter was included for some methods with either a 12 s or 3 s exclusion window and had common parameters of: exclude n = 1 times; +/−3 ppm; exclude isotopes; and single charge state per precursor. Apex detection was included for one method and was set to: expected peak width, 6 s; desired apex window, 30%. There were five ddMS^2^ OT-HCD scans with the following settings unless stated otherwise: quadrupole isolation, 1.6 *m/z* isolation window; HCD collision energy, 40%, stepped 5%; detector type, Orbitrap, auto *m/z* normal scan range, 15 K resolution, 100 m/z first mass; AGC Target, 5e4, inject ions for all available parallelizable time, 35 ms maximum injection time; 1 µscan, profile. A targeted reporter ion trigger (TRT) followed ddMS^2^ OT-HCD and included ions 249.040, 275.019, and 300.998; +/− 5 ppm error tolerance; with the detection of either 3 or 2 or 1 ions from the list as explicitly stated; only ions within the top 10 most intense for all mass triggers. Subsequent ddMS^2^ OT-CID conditions were as follows unless stated otherwise: MS^n^ Level, 2; quadrupole isolation, 1.6 *m/z* isolation window; CID collision energy, 30; activation Q, 0.25; detector type, Orbitrap, auto *m/z* normal scan range, 15 K resolution; AGC Target, 5e4, inject ions for all available parallelizable time, 22 ms maximum injection time; 1 µscan, profile. The number of dependent scans between ddMS^2^ OT-HCD and ddMS^2^ OT-CID was set to 1. A summary of screen method parameters for a given TRT condition is presented in Table 3. A more complete description of the motivation for the application of each filter combination can be found in the Supporting Information.

**Table 3:** Screen specific modifications of applied filters before precursor selection.

Screen	Description	Intensity Filter	Charge Filter	DE	Apex Detection
I.	(Intensity)	1e5	N/A	N/A	N/A
II.	(Anion)	1e5	−1	N/A	N/A
III.	(Dianion)	1e5	−2	N/A	N/A
IV.	(DE 12 s)	1e5	N/A	t = 12 s	N/A
V.	(DE 3 s)	1e5	N/A	t = 3 s	N/A
VI.	(DE 3 s, Apex)	1e5	N/A	t = 3 s	6 s, 30%
VII.	(−1, DE 3 s)	1e5	−1	t = 3 s	N/A
VIII.	(−2, DE 3 s)	1e5	−2	t = 3 s	N/A
IX.	(DE 3 s, 5e5)	5e5	N/A	t = 3 s	N/A
X.	(−1, DE 3 s, 5e5)	5e5	−1	t = 3 s	N/A
XI.	(−2, DE 3 s, 5e5)	5e5	−2	t = 3 s	N/A

Common settings between methods included: OT-MS Scan: 50 K; MIPS Filter: Peptide; 5 ddMS2 OT-HCD Scans: HCD CE 40 +/− 5, 15 K; TRT: T-3RT or T-2RT as stated; 1 ddMS2 OT-CID Scan: CE 30, 15 K.

### Data Analysis

Description of software used for analysis and figure creation can be found in the Supporting Information.

### Data Availability

The MS/MS datasets generated during the current study are available in the figshare repository, https://figshare.com/projects/Rapid_Screening_of_Ellagitannins_in_Natural_Products_via_Targeted_Reporter_Ion_Triggered_Tandem_Mass_Spectrometry/29656.

## Electronic supplementary material

Supplementary Information
