# Supplementary material for: Rapid Screening of Ellagitannins in Natural Sources via Targeted Reporter Ion Triggered Tandem Mass Spectrometry
Source: Sci Rep. 2018 Jul 10;8:10399. doi: 10.1038/s41598-018-27708-3 (PMC6039434; doi:10.1038/s41598-018-27708-3)
Supplement: Supplementary file 1 — Supplementary Information [file 41598_2018_27708_MOESM1_ESM.pdf]

Rapid Screening of Ellagitannins in Natural Sources via Targeted Reporter Ion Triggered Tandem Mass Spectrometry

Jeremiah J. Bowers,[a] Harsha P. Gunawardena,[b] Anaëlle Cornu,[c] Ashwini S. Narvekar,[a] Antoine Richieu,[c] Denis Deffieux,[c] Stéphane Quideau,[c] and Nishanth Tharayil[a*]

Department of Plant and Environmental Sciences
Clemson University
Clemson, SC 29631

[a]Department of Plant and Environmental Sciences, Clemson University, Clemson, SC 29631

[b]Janssen Research and Development, The Janssen Pharmaceutical Companies of Johnson and Johnson, Spring House, PA 19477

[c]Univ. Bordeaux, ISM (CNRS-UMR 5255), 351 cours de la Libération, 33405 Talence Cedex, France

* Corresponding author: Dr. Nishanth Tharayil
E-mail: ntharay@clemson.edu
Tel: (864) 656-4453

Abstract:

Further information on sample preparation, data extraction, analyzer and filter optimization, chemical structures of standard compounds, CID spectra of standard compounds, elution and infusion MS of standard compounds, HCD summary tables and figures, standard dilution EIC profiles, TRT specificity and control figures, full tables of species classified as ellagitannins under different screen conditions, figures illustrating data points for screen and TRT variants of the 466 strawberry isobars, and annotated tables for comparison of previously reported non-isomeric ellagitannins is contained herein. A full table of contents has been provided for ease of navigation.

Supporting Information

Table of Contents

Experimental S-6
Strawberry leaf extraction S-6
Infusion MS/MS analysis S-6
Data Analysis S-7
Software S-7
Proteome Discoverer workflow S-7
Perl and Excel workflow S-8
Results and Discussion S-9
Analyzer Optimization S-9
Mass Range, MIPS, Intensity, and Z Filters S-9
HCD Dependent Scans, DE, and Apex Detection S-9
MS Analyzer: Resolution and Injection Time S-10
CID Analyzer: OT vs IT, Resolution and Injection Time S-10
Mass Trigger Validation S-11
Control Experiments S-11
Filter Optimization S-12
Proteome Discoverer, Isobaric Grouping Parameters S-12
Screen Selection Parameters S-13
Supplementary Figures S-14
Figure S-1: Molecular Structures, Ellagitannin Standards S-14
Figure S-2: CID, Higher Charge States, MS^2 .... S-15
Figure S-3: CID 457, MS^3 .... S-16
Figure S-4: CID 487 MS^3, 465 MS^4 .... S-17
Figure S-5: CID, Lower Charge States, MS^2 .... S-18
Figure S-6: Infusion MS S-19
Figure S-7: HCD Series, $[M-2H]^{2-}$ S-20
Figure S-8: Elution MS S-21

Figure S-9: HCD Series, $[M-3H]^{3-}$ S-22
Figure S-10: MS^2 Spectra – CID 20 and HCD 20 .... S-23
Figure S-11: CID Data Points – MS 50K, 50 ms S-24
Figure S-12: CID Data Points – MS 30K, 50 ms S-25
Figure S-13: CID Data Points – MS 15K, 50 ms S-26
Figure S-14: CID Data Points – MS 50K, 25 ms S-27
Figure S-15: CID Data Points – MS 30K, 25 ms S-28
Figure S-16: CID Data Points – MS 15K, 25 ms S-29
Figure S-17: CID Data Points – CID 50K, AGC 5e4 .... S-30
Figure S-18: CID Data Points – CID 30K, AGC 5e4 .... S-31
Figure S-19: CID Data Points – CID 15K, AGC 5e4 .... S-32
Figure S-20: CID Data Points – CID 50K, AGC 1e4 .... S-33
Figure S-21: CID Data Points – CID 30K, AGC 1e4 .... S-34
Figure S-22: CID Data Points – CID 15K, AGC 1e4 .... S-35
Figure S-23: CID Data Points – IT Normal, AGC 1e4 .... S-36
Figure S-24: CID Data Points – IT Rapid, AGC 1e4 .... S-37
Figure S-25: CID Data Points – IT Turbo, AGC 1e4 .... S-38
Figure S-26: CID Spectra – Orbitrap vs Ion Trap S-39
Figure S-27: CID EICs – Standards, 40 ng to 1.25 ng S-40
Figure S-28: CID Data Points – Standards, 5 ng S-41
Figure S-29: CID Data Points – Standards, 2.5 ng S-42
Figure S-30: CID Data Points – Standards, 1.25 ng S-43
Figure S-31: CID Data Points – Single Isomeric Injections S-44
Figure S-32: Mixture Specificity, T-3RT S-45
Figure S-33: Mixture Specificity, T-2RT S-46
Figure S-34: Mixture Specificity, T-1RT S-47
Figure S-35: Mass Trigger Controls, T-3RT S-48
Figure S-36: Mass Trigger Controls, T-2RT S-49
Figure S-37: Mass Trigger Controls, T-1RT S-50
Figure S-38: HCD Product Ions - Potential Interferents S-51
Figure S-39: CID Data Points, 466 *m/z* – Screen Variants, T-3RT S-52
Figure S-40: CID Data Points, 466 *m/z* – Screen Variants, T-2RT S-53

Figure S-41: Proposed Structures – Ions of Interest S-54
Supplementary Tables S-55
Table S-1: Precursor CID, 457 Major Product Ion S-55
Table S-2: Precursor CID, 487 Major Product Ion S-55
Table S-3: CID 457, MS^3 .... S-56
Table S-4: CID 487, MS^3 .... S-56
Table S-5: CID 465, MS^4 .... S-56
Table S-6: ∑ (249, 275, 301) Intensities, Discrete HCD Series S-57
Table S-7: ∑ (249, 275, 301) Intensities, Theoretical Stepped (+/-10) HCD Series S-57
Table S-8: ∑ (249, 275, 301) Intensities, Stepped HCD Series S-58
Table S-9: Strawberry, Screen [I], T-3RT S-59
Table S-10: Strawberry, Screen [II], T-3RT S-60
Table S-11: Strawberry, Screen [III], T-3RT S-61
Table S-12: Strawberry, Screen [IV], T-3RT S-62
Table S-13: Strawberry, Screen [V], T-3RT S-64
Table S-14: Strawberry, Screen [VI], T-3RT S-66
Table S-15: Strawberry, Screen [VII], T-3RT S-67
Table S-16: Strawberry, Screen [VIII], T-3RT S-68
Table S-17: Strawberry, Screen [IX], T-3RT S-69
Table S-18: Strawberry, Screen [X], T-3RT S-70
Table S-19: Strawberry, Screen [XI], T-3RT S-71
Table S-20: Strawberry, Screen [I], T-2RT S-72
Table S-21: Strawberry, Screen [II], T-2RT S-74
Table S-22: Strawberry, Screen [III], T-2RT S-75
Table S-23: Strawberry, Screen [IV], T-2RT S-78
Table S-24: Strawberry, Screen [V], T-2RT S-83
Table S-25: Strawberry, Screen [VI], T-2RT S-87
Table S-26: Strawberry, Screen [VII], T-2RT S-91
Table S-27: Strawberry, Screen [VIII], T-2RT S-93
Table S-28: Strawberry, Screen [IX], T-2RT S-97
Table S-29: Strawberry, Screen [X], T-2RT S-99
Table S-30: Strawberry, Screen [XI], T-2RT S-100

Table S-31: Strawberry, Summary of Screens [I-XI], T-3RT S-102
Table S-32: Referenced Annotated Ellagitannins S-103
Table S-33: Screen [IV], T-2RT Comparison Match Strawberry Leaves S-107
Table S-34: Screen [IV], T-2RT Comparison Match Any Source S-110
References: S-119

Experimental

Strawberry leaf extraction

Initially 500 µL of methanol was added to the provided dried extracts of each species after which 5 µL of these were diluted with methanol to a final volume of 500 µL to create final concentrations of 250 µM. Harvested strawberry (*Fragaria ananassa* cv. Albion) and Palmer amaranth (*Amaranthus palmeri*) leaf tissues were initially stored at -80° C for 18 hours after which a mortar and pestle was utilized to finely powder them with dry ice. The mixture was stored at -20° C for 24 hours to allow for complete sublimation before weighing out 100 mg into 1 mL of methanol. Homogenization by sonication was then performed by utilizing two cycles of 40s at 50% amplitude followed by 20s in an ice bath for a total of two minutes per sample. Samples were subsequently vortexed and then centrifuged at 1048g for 10 min at 25° C after which 0.5 mL of supernatant was transferred to new precooled microfuge tubes. Then 0.25 mL of water and 0.25 mL of chloroform were added followed by 20 inversions before subsequent centrifugation at 1048g for 10 min at 25° C. Approximately 0.1 mL of the aqueous-methanol phase was transferred to LC-MS vials for analysis. Ellagitannins pose no known health hazard and do not require specialized handling procedures.

Infusion MS/MS analysis

Ellagitannin standards were examined by direct infusion utilizing a flow rate of 5 µL to 10 µL per minute. The mass spectrometer was operated in negative ionization mode. The interface conditions were as follows: emitter voltage, -2600V; vaporizer temperature, 30° C; ion transfer tube, 275° C; sheath gas, 12 (arb); aux gas, 5 (arb); and sweep gas, 0 (arb). Internal mass spectrometer settings utilized were as follows: RF lens, 60%; AGC target, 4e5; maximum injection time, 50 ms; and quadrupole isolation width, 2 *m/z*. HCD and CID energies were applied as described in corresponding figures and tables. Mass ranges selected for MS/MS were: roburin E, grandinin, castalagin, and vescalagin, (150-1200 *m/z*); acutissimin A and epiacutissimin A, (150-1250 *m/z*); roburin A, roburin D, and roburin B/C, (200-2000 *m/z*).

Data Analysis

Software

Data analysis was performed with Freestyle visualization software 1.1.175.0 (Thermo Scientific), Proteome Discoverer 2.1.0.81 (Thermo Scientific), ActivePerl-5.24.1.2402-MSWin32-x64-401627 (ActiveState Inc., Vancouver, BC, CA), and Microsoft Excel Office 365 (Microsoft Corp., Redmond, WA, USA). Freestyle display options for chromatograms were set to centroid and scan filters were manually selected from the filter dropdown menu. Specific workflows for Proteome Discoverer and Perl are provided in the following sections. Mass Spectra were annotated with Microsoft Powerpoint Office 365 (Microsoft Corp.) and chemical structures were created in Biovia Draw 2017 v17.1 (Dassault Systemes, BIOVIA Corp., San Diego, CA) and ChemDraw 2017 v17.0.0.206 (PerkinElmer Informatics, Inc., Waltham, MA).

Proteome Discoverer workflow

The workflow tree for Proteome Discoverer to process (*.raw) files consisted of four serial nodes: Spectrum Files, [Node_0]; Spectrum Selector, [Node_1]; Scan Event Filter, [Node_2]; Spectrum Grouper, [Node_3]. After Spectrum Grouper two Scan Event Filters were employed in parallel, [Node_6] and [Node_4], each of which had a Spectrum Exporter connected in series, [Node_7] and [Node_5] respectively. Specific settings for nodes used were as follows unless stated otherwise: [Node_1] – 1. General Settings: Use MS^1 Precursor Selection, 2. Spectrum Properties Filter: Min. Precursor Mass = 150 Da, Max Precursor Mass = 2400 Da, Minimum Peak Count = 1, all other values = 0, 3. Scan Event Filters: Mass Analyzer = (Not Specified), MS Order = Any, Activation Type = Any, Min. Collision Energy = 0, Max Collision Energy = 1000, Scan Type = Is Full, Polarity Mode = (Not Specified), 4. Peak Filters: S/N Threshold (FT-Only) = 0, 5. Replacements for Unrecognized Properties: U. Charge R. = Automatic, U. Mass Analyzer R. = FTMS, U. MS Order R. = MS^2, U. Activation Type R. = CID, U. Polarity R. = -, U. MS Resolution @ 200 R. = 50000, U. MS^n Resolution @ 200 R. = 15000. [Node_2] – 1. Filter Settings: Mass Analyzer = (Not Specified), MS Order = Is MS^2, Activation Type = Any, Min. Collision Energy = 0, Max Collision Energy = 1000, Scan Type = (Not Specified), Polarity

Mode = (Not Specified). [Node_3] – 1. Spectrum Match Criteria: Precursor Match Criterion = Same Measured Mass-to-Charge, Precursor Mass Tolerance = 10 ppm, Max. RT Difference [min] = 13, Allow Mass Analyzer Mismatch = False, Allow MS Order Mismatch = False. [Node_4] – 1. Filter Settings: Mass Analyzer = (Not Specified), MS Order = Is MS^2, Activation Type = Is HCD, Min. Collision Energy = 0, Max Collision Energy = 1000, Scan Type = (Not Specified), Polarity Mode = (Not Specified). [Node_5] – 1. Output Data: File Name = (no entry), File Name Suffix = HCD, Export Format = Mascot Generic Format (*.mgf). [Node_6] – 1. Filter Settings: Mass Analyzer = (Not Specified), MS Order = Is MS^2, Activation Type = Is CID, Min. Collision Energy = 0, Max Collision Energy = 1000, Scan Type = (Not Specified), Polarity Mode = (Not Specified). [Node_7] – 1. Output Data: File Name = (no entry), File Name Suffix = CID, Export Format = Mascot Generic Format (*.mgf).

Perl and Excel workflow

A perl script was written to process the (*.mgf) files associated with [Node_7] into corresponding (*.csv) files. The output csv file contained columns: File = raw file name; Pepmass = precursor *m/z*; Intensity = NL; Charge = z; RT in seconds = time range precursors were observed; Scans = scan range precursors were observed. Output files in mgf format were placed in a common folder along with the mgf-to-csv.pl script within the path, (C:\Perl64\). Command line execution from within the common folder to create csv files used the specific syntax: perl mgf-to-csv.pl input_filename.mgf output_filename.csv. Output csv files were converted to full Excel workbooks in xlsx format. The MAX, COUNTIF, and COUNTIFS functions were then used to generate summary values in each excel file.

Results and Discussion

Analyzer Optimization

Mass Range, MIPS, Intensity, and Z Filters

The mass range was set from 150 *m/z* to 1200 *m/z* for mass spectra as standards eluted primarily as $[M-2H]^{2-}$ ions, but a dynamic software adjustment was allowed for both HCD and CID spectra. This allowed for monitoring of any incidental in-source fragmentation that may produce 249 / 275 / 301 reporter ions as an indicator of problems in precursor ion transmission. The lower value of 150 *m/z* was chosen to ensure sugars such as glucose could be observed but high abundance solvent ions would not adversely affect ion accumulation of analyte ions due to the AGC applied to the FT analyzer. The upper range of 1200 *m/z* was selected to balance minimizing total scanning time to enable more data points to be collected while still allowing for observation of multiply charged ellagitannins. Larger standards formed multiply charged species preferentially and it seemed reasonable to expect increasing charge states with ellagitannin size so 1200 *m/z* was selected to allow for the observation of more ellagitannins for this pilot study. The heated capillary reduced the transmission of non-covalent multimers (i.e. dimers, etc.) and additional in source fragmentation was not required for standard compounds. Isotopes of precursors were excluded by applying the peptide setting of the monoisotopic peak selection (MIPS) filter. An intensity filter was used to discriminate initial precursor ions with values of 1e5 and 5e5 chosen for comparison. Inclusion of a dianion charge state filter was implemented for standard analysis.

HCD Dependent Scans, DE, and Apex Detection

The number of data dependent HCD scans was set to 5. This value ensured that all eligible precursors could also be subjected to CID. Values higher than 5 resulted in HCD being prioritized over CID with fewer eligible precursors being subjected to CID before the next MS scan. An optional dynamic exclusion (DE) filter was considered. The apexes of the closest resolved standard isomers were roughly 24s apart and half that value was initially selected as an exclusion window. Species with narrow elution profiles widths of

approximately 6s observed in strawberry motivated the inclusion of a 3s exclusion window in later filter iterations. Dynamic exclusion only impacted the number of data points acquired for standard compounds and was not explicitly discussed. Apex detection was included for one DE method for comparison.

MS Analyzer: Resolution and Injection Time

Analyzer conditions for initial MS spectra were varied by Orbitrap resolution (at *m/z* 200) and maximum injection times to determine the impact on the number of CID spectra that could be acquired. Discrete CID data points of the $[M\text{-}2H]^{2-}$ ion for 40 ng of each ellagitannin acquired with a maximum injection time of 50 ms or 25 ms at an Orbitrap resolution of 50K, 30K, and 15K appear in Figures S-11 to S-13 and Figures S-14 to S-16 respectively. Given the transient lengths for 50K, 30K, and 15K were 96 ms, 64 ms, and 32 ms it was unsurprising that there were generally a few more data points at lower resolution settings at either maximum injection time. Since resolution settings of 500K, 240K, and 120K with their substantially longer transients of 1024 ms, 512 ms, and 256 ms were not utilized, the MS resolution setting chosen had less effect on the number of CID data points acquired. A 50K resolution with a 50 ms maximum ion accumulation was selected. Inspection of precursor scans from strawberry suggested that higher resolutions than this may be beneficial for some low abundance ellagitannins with narrow elution profiles but resolution settings of 30K or 15K also provided equivalent screening performance under most conditions.

CID Analyzer: OT vs IT, Resolution and Injection Time

Analyzer conditions for CID spectra were varied by analyzer and automatic gain control (AGC) values to determine the impact on the number of CID spectra that could be acquired in addition to spectra quality. Discrete CID data points of the $[M\text{-}2H]^{2-}$ ion for 40 ng of each ellagitannin acquired using an AGC Target of 5e4 or 1e4 at an Orbitrap resolution of 50K, 30K, and 15K appear in Figures S-17 to S-19 and Figures S-20 to S-22 respectively. Similar data acquired with an AGC value of 1e4 at Ion Trap scan rates of Normal, Rapid, and Turbo are shown in Figures S-23 to S-25. It was observed that the default 5e4 AGC settings with 15K resolution for Orbitrap CID spectra was comparable in

terms of number of data points generated for isomer differentiation but superior in mass accuracy as well as resolution over the Ion Trap. This difference in mass accuracy between spectra generated by the Orbitrap and Ion Trap was apparent even for castalagin which had the widest elution profile and the most number of data points for averaging as shown in Figure S-26. Lowering the number of target ions by reducing the AGC setting to 1e4 on the Orbitrap did increase the number of CID data points but also slightly reduced the relative intensity lower abundance fragments. The Thermo default of 5e4 for 15K Orbitrap CID scans was retained to ensure the abundance of low intensity fragment ions was not reduced for marginal method time improvement.

Mass Trigger Validation

Control Experiments

The functionality of the conditional TRT used to initiate acquisition of CID data was first evaluated by estimating a practical lower bound for ellagitannin injections. The CID extracted ion chromatograms (EICs) of 40 ng to 1.25 ng injections (Figure S-27) indicated that either 5 ng or 2.5 ng might be the minimum amount of an ellagitannin required. Closer inspection of the discrete CID data points generated for 5 ng, 2.5 ng, and 1.25 ng injections (Figure S-28 to S-30) showed that all ellagitannin standards maintained similar numbers of data points through 1.25 ng but shoulder truncation became more pronounced below 2.5 ng. This implied that 2.5 ng would be the minimum amount required to ensure limited truncation for species classified as ellagitannins. Caution must be advised when selecting CID data points for averaging given that close eluting isomers may or may not have data points between the apexes (Figure S-31) that have non-trivial contributions from each isomer.

To examine the specificity of TRT amaranth was selected as a test matrix as it contained no ellagitannin or HHDP derivatives that should result in classification of any precursor as an ellagitannin. TRT conditions were examined to compare the differences of requiring all three reporter ions in T-3RT and variants that allowed any two reporter ions (T-2RT) or any reporter ion (T-1RT) to be observed in the top 10 most abundant peaks in an HCD spectrum to result in CID acquisition. A 10 µg/mL sample of mixed standard

ellagitannins, 100 mg/mL of amaranth, and a mixed sample of standard ellagitannins and amaranth at the same final concentrations were prepared. Each ellagitannin standard was correctly screened (Figures S-32 to S-34) and subjected to CID in both the ellagitannin and mixed amaranth / ellagitannin samples under all TRT conditions. The slight improvement observed for standards present in the mixture could either be resultant from more complete desorption from the column or greater ion transmission efficiency through the heated capillary. Repeating this experiment on a mass spectrometer without a capillary interface could readily exclude the latter possibility. A full critique of the quantitative limitations of the tandem HCD-CID screen on the Fusion platform was beyond the scope of this work.

The charge state filter was then modified to allow precursor candidates present in amaranth that were anions, dianions, or both anions and dianions to be eligible for HCD to allow for the possibility of incorrectly classified precursors. Inspection of the resultant MS, HCD, and CID chromatograms showed that no precursors were incorrectly classified as ellagitannins and subjected to CID in amaranth when a T-3RT (Figure S-35) or T-2RT (Figure S-36) was employed. Two false positive classifications were observed for T-1RT conditions (Figure S-37) which resulted in the acquisition of CID data in the absence of ellagitannin content. Strawberry analysis omitted T-1RT analysis given the false positives observed in amaranth.

Filter Optimization

Proteome Discoverer, Isobaric Grouping Parameters

In brief, the [Node_1] peak filters S/N threshold value resulted in the exclusion of species with fewer data points in methods that utilized dynamic exclusion and zero was chosen so no species classified as ellagitannins would be omitted when the Proteome Discoverer workflow was used that would be manually included if Freestyle was employed. In [Node_3] both the precursor mass tolerance and maximum RT difference were significant parameters. A precursor mass tolerance of 10 ppm was chosen to ensure that precursors +/- 5 ppm would be grouped and an RT value of 13 minutes was chosen to ensure a single *m/z* value would group all potential isomers. Although smaller RT values of 1.5 min, 1.0 min, 0.5 min, 0.13 min, and 0.05 min were examined, Proteome Discoverer

was unable to correctly categorize isomers by expected RT widths as there was too much inherent variation between species. Additional post-analysis workflow that allows spectral library matching to enumerate and associate isomers with unique CID spectra was beyond the scope of this work.

Screen Selection Parameters

Screen [I] provided information on the effectiveness of utilizing only an intensity filter and five data dependent HCD scans. Tannins had previously been reported as readily forming doubly charged anions via ESI and the elution mass spectra of standard ellagitannins (Figure S-8) supported that observation.[1] Screen [II] and [III] were applied to preferentially monitor low mass and high mass ellagitannins respectively. Allowing for both anions and dianions was no different than not having a charge state filter given the ionization constraints during elution and was not included. Condition [IV] was initially estimated from standard elution profiles but additional acquisition under [V] was performed to ensure later eluting species with potentially narrower elution profiles were not omitted. Apex detection was combined with DE in condition [VI] to determine if more low abundance species were classified with the addition of this feature. Combinations that included both charge state and DE filters were examined in [VII] and [VIII] to determine if any advantage in compound discovery was gained. Additional DE variants with higher precursor intensity filters were created in [IX], [X], and [XI] to allow for greater number of higher abundance species to be catalogued if present.

Supplementary Figures

Figure S-1: Molecular Structures, Ellagitannin Standards

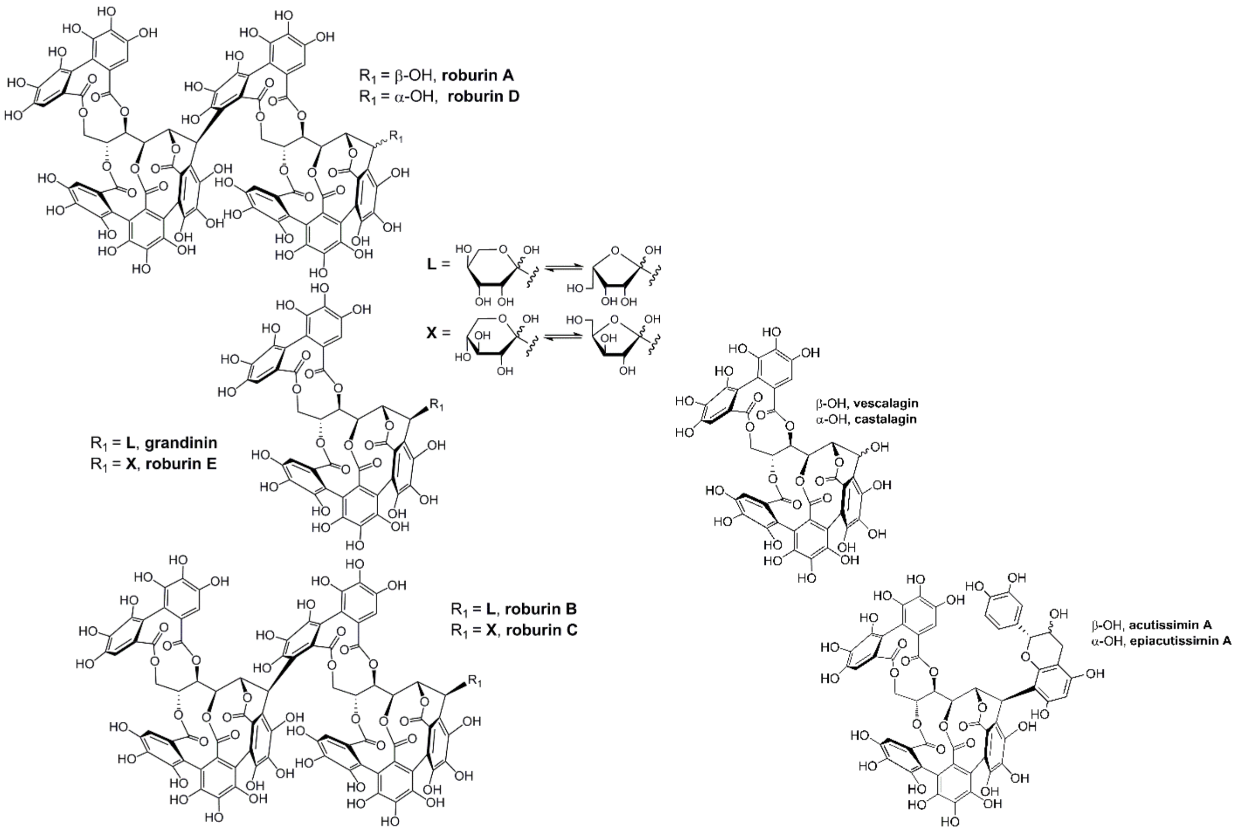

Figure S-1: Molecular structures of acutissimin A, epiacutissimin A, roburin A, grandinin, roburin E, roburin D, roburin B/C, vescalagin, and castalagin.

Figure S-2: CID, Higher Charge States, MS^2

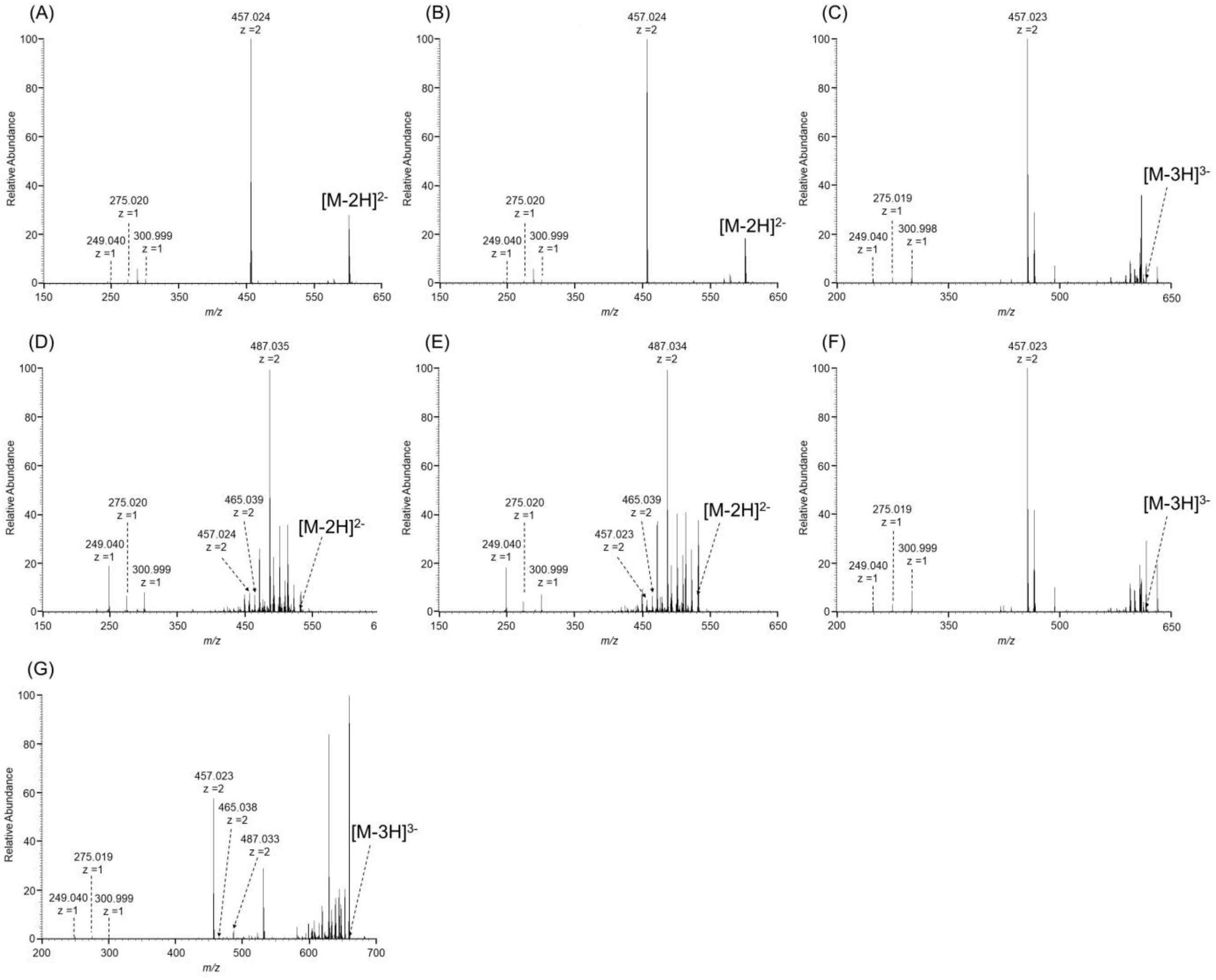

Figure S-2: CID spectra of: (A) acutissimin A $[M-2H]^{2-}$, (B) epiacutissimin A $[M-2H]^{2-}$, (C) roburin A $[M-3H]^{3-}$, (D) grandinin $[M-2H]^{2-}$, (E) roburin E $[M-2H]^{2-}$, (F) roburin D $[M-3H]^{3-}$, (G) roburin B/C $[M-3H]^{3-}$.

Figure S-3: CID 457, MS^3

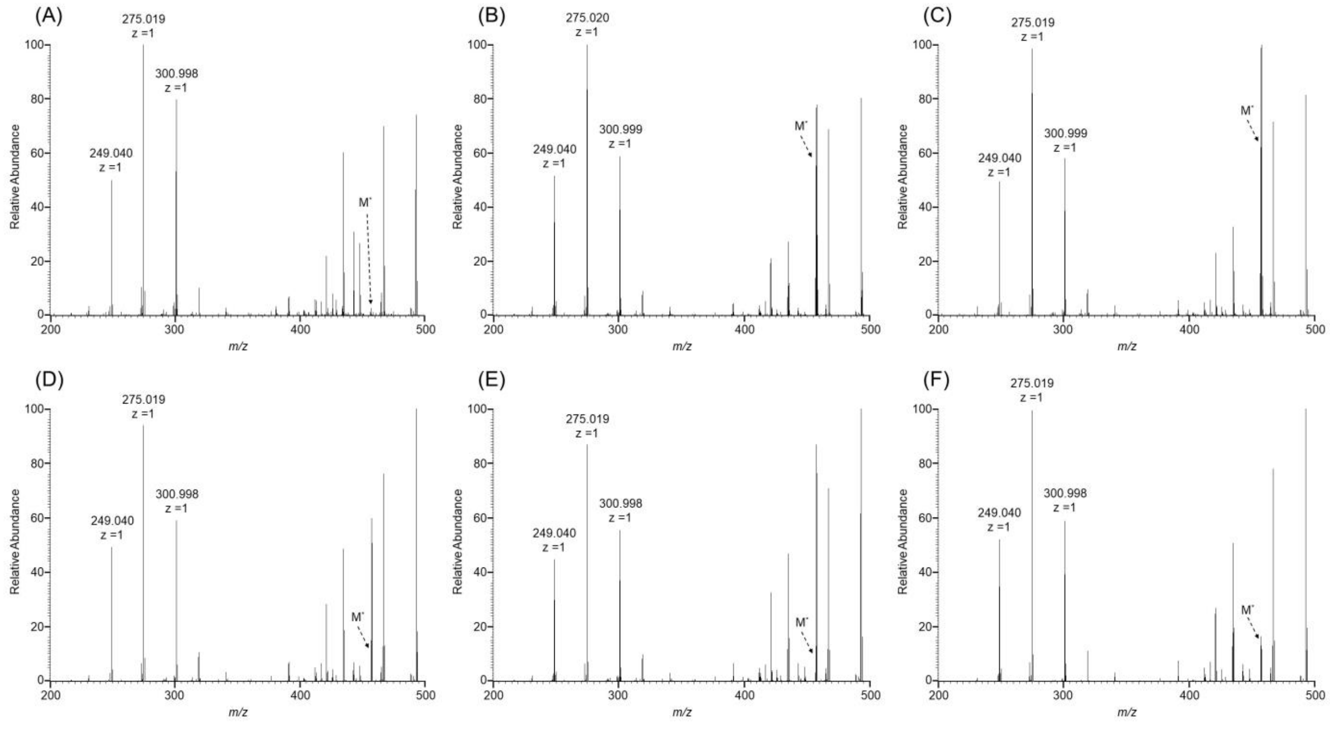

Figure S-3: CID spectra of the isolated 457 species derived from the collision-induced dissociation of: (A) castalagin $[M-2H]^{2-}$, (B) acutissimin A $[M-2H]^{2-}$, (C) epiacutissimin A $[M-2H]^{2-}$, (D) roburin A $[M-3H]^{3-}$, (E) roburin D $[M-3H]^{3-}$, (F) roburin B/C $[M-3H]^{3-}$. The corresponding spectra for Vescalagin appears in the main text and was not duplicated here.

Figure S-4: CID 487 MS^3, 465 MS^4

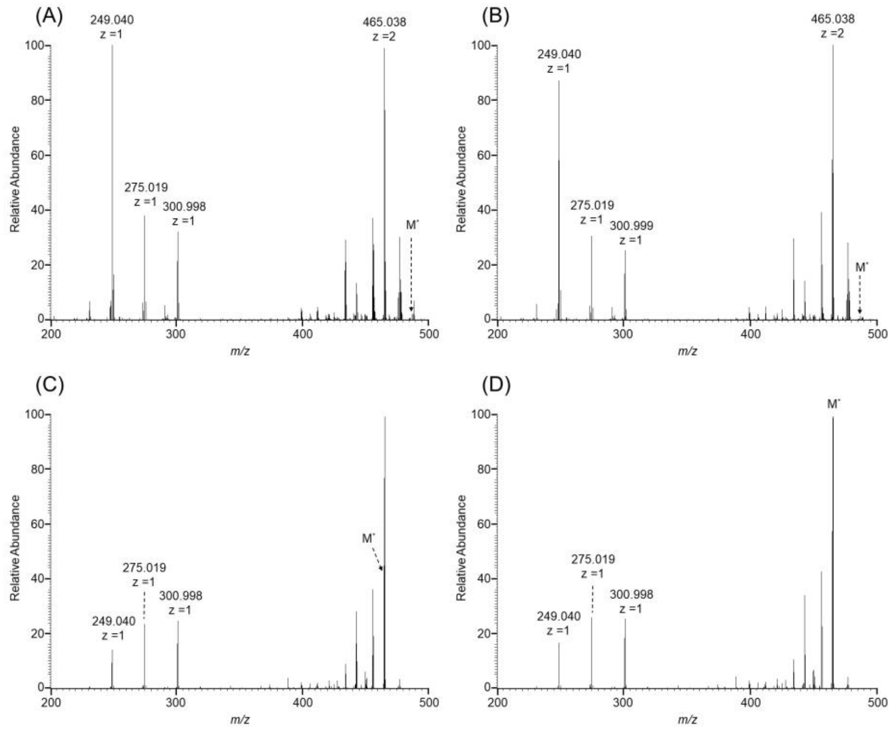

Figure S-4: CID spectra of the isolated collision-induced dissociation of: (A) grandinin 487, MS^3, (B) roburin E 487, MS^3, (C) grandinin 465, MS^4, (D) roburin E 465, MS^4.

Figure S-5: CID, Lower Charge States, MS^2

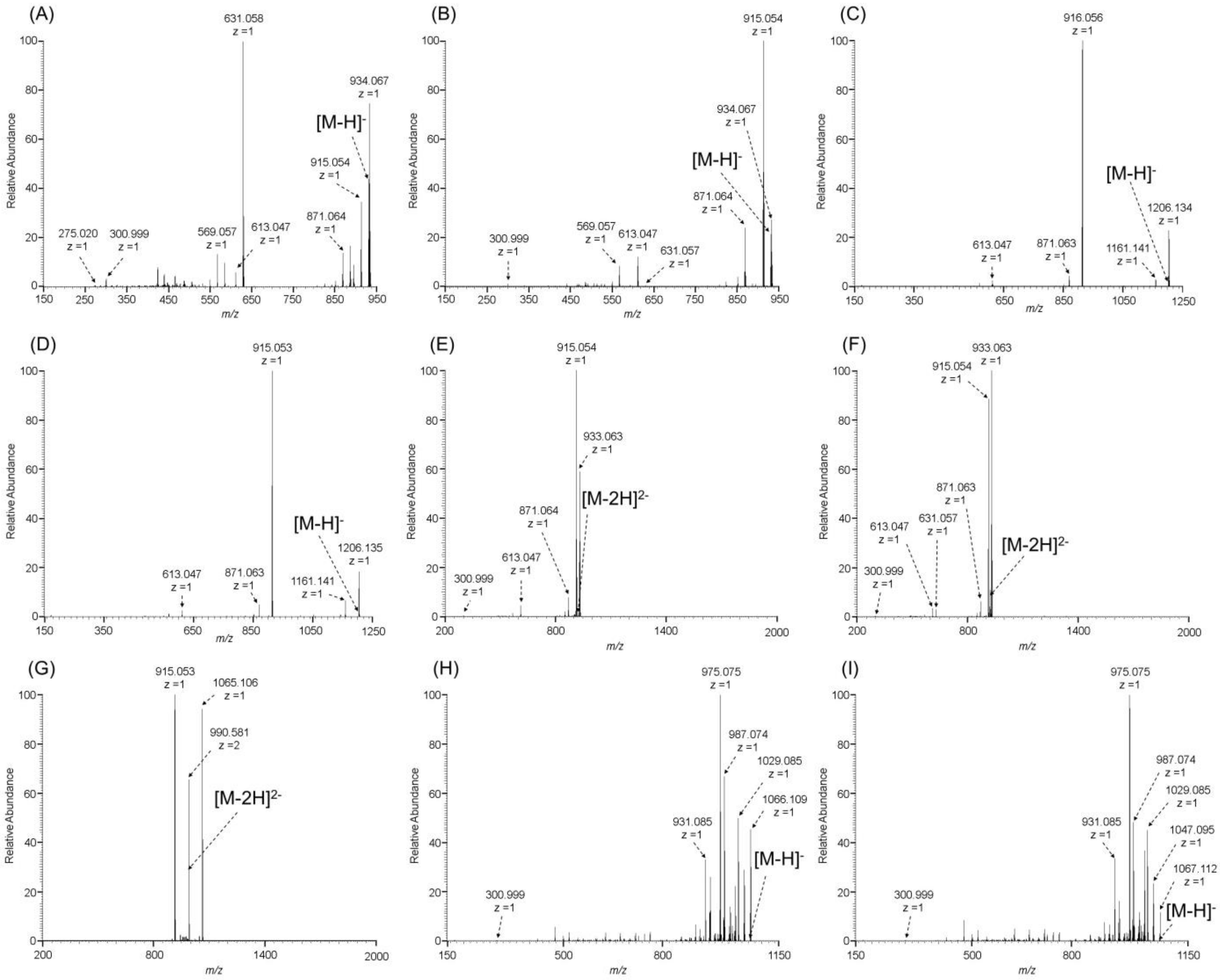

Figure S-5: CID spectra of: (A) castalagin [M-H]$^-$, CE 25; (B) vescalagin [M-H]$^-$, CE 24; (C) acutissimin A [M-H]$^-$, CE 25; (D) epiacutissimin A [M-H]$^-$, CE 25; (E) roburin A [M-2H]$^{2-}$, CE 20; (F) roburin D [M-2H]$^{2-}$, CE 18; (G) roburin B/C [M-2H]$^{2-}$, CE 16; (H) grandinin [M-H]$^-$, CE 25; (I) roburin E [M-H]$^-$, CE 28.

Figure S-6: Infusion MS

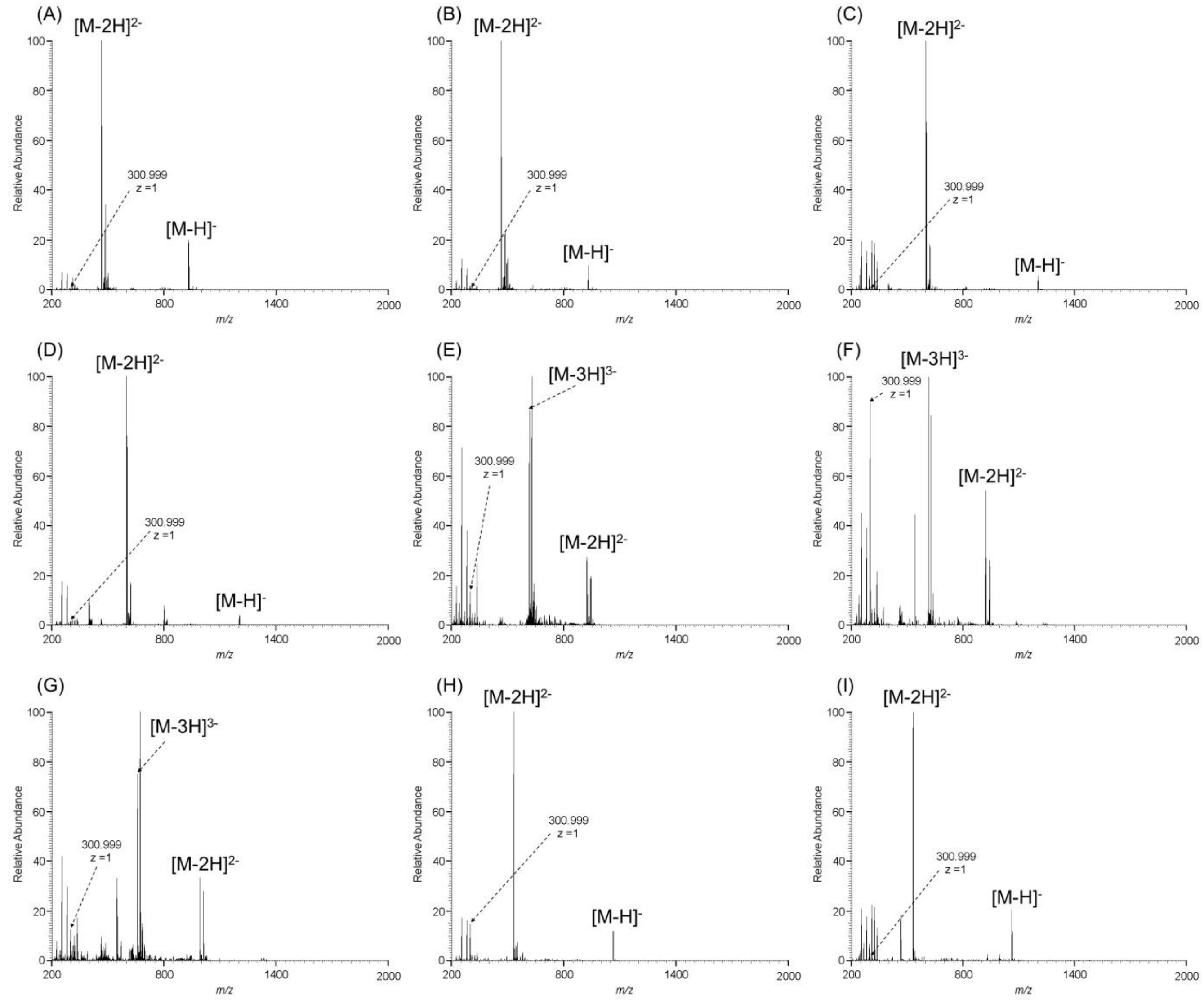

Figure S-6: Infusion mass spectra of: (A) castalagin, (B) vescalagin, (C) acutissimin A, (D) epiacutissimin A, (E) roburin A, (F) roburin D, (G) roburin B/C, (H) grandinin, (I) roburin E.

Figure S-7: HCD Series, $[M-2H]^{2-}$

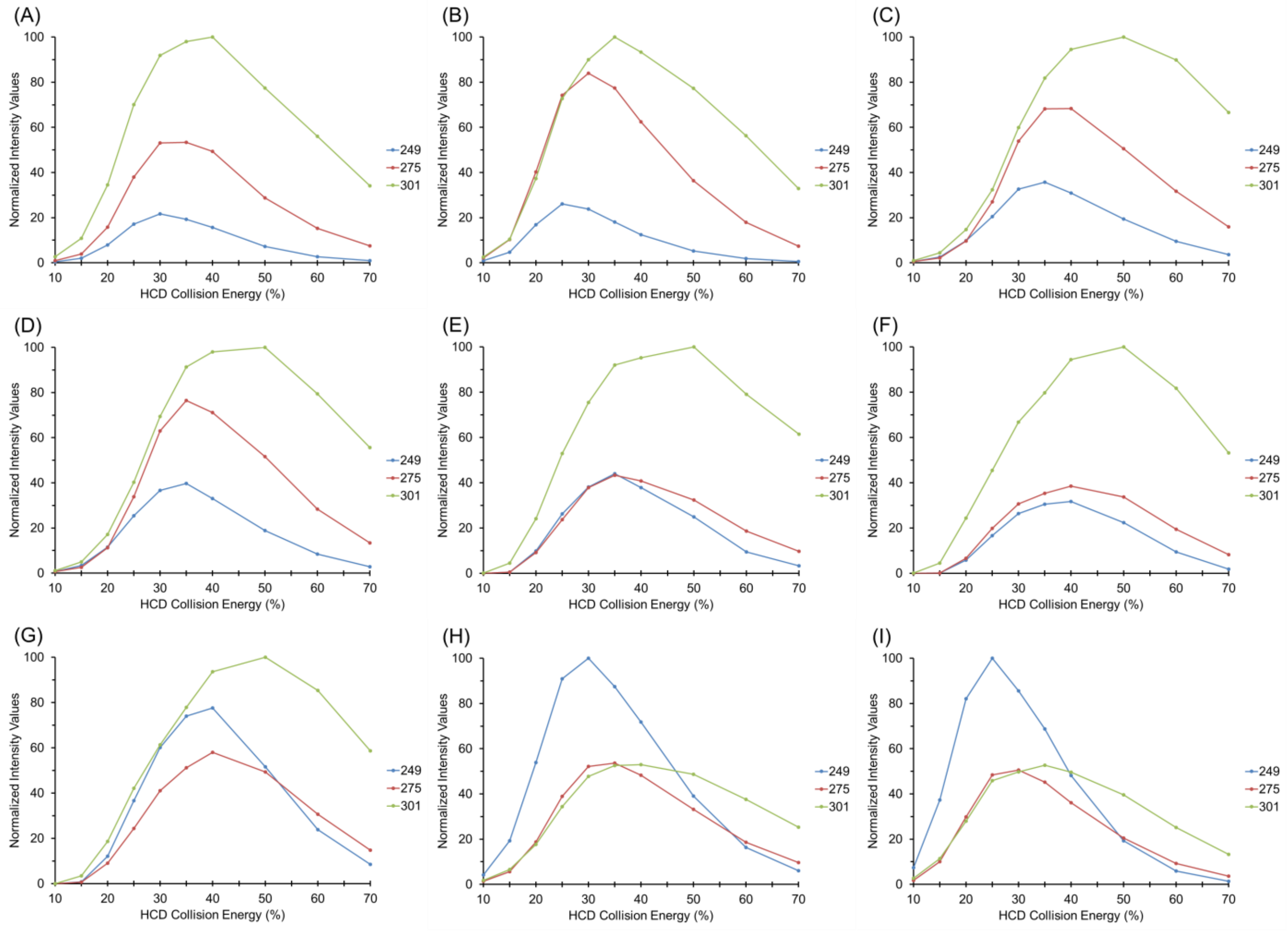

Figure S-7: Normalized abundance of (249, 275, 301) ions with HCD collision energy of: (A) castalagin $[M-2H]^{2-}$, (B) vescalagin $[M-2H]^{2-}$, (C) acutissimin A $[M-2H]^{2-}$, (D) epiacutissimin A $[M-2H]^{2-}$, (E) roburin A $[M-2H]^{2-}$, (F) roburin D $[M-2H]^{2-}$, (G) roburin B/C $[M-2H]^{2-}$, (H) grandinin $[M-2H]^{2-}$, (I) roburin E $[M-2H]^{2-}$.

Figure S-8: Elution MS

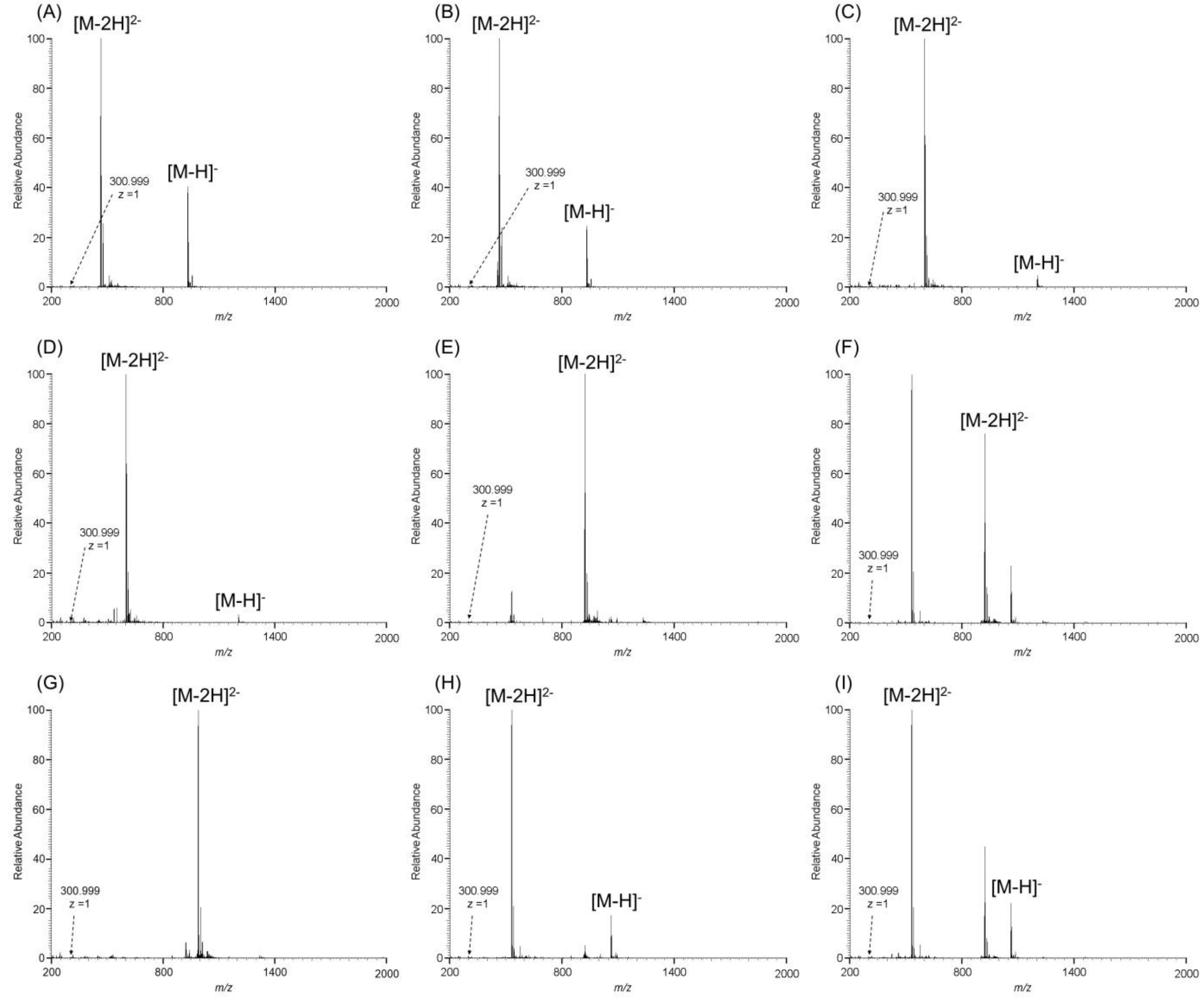

<u>Figure S-8</u>: Injection of a 20 ng standard mixture and resultant mass spectra acquired during the apex of the elution profile of: (A) castalagin (5.37-5.56 min), (B) vescalagin (3.70-3.93 min), (C) acutissimin A (7.15-7.30 min), (D) epiacutissimin A (8.88-8.95 min), (E) roburin A (2.73-2.89 min), (F) roburin D (3.06-3.20 min), (G) roburin B/C (2.48-2.74 min), (H) grandinin (2.88-3.03 min), (I) roburin E (3.11-3.33 min). The unlabeled significant peaks in (E), (F), (H), and (I) are resultant from the presence of coeluting standards.

Figure S-9: HCD Series, $[M-3H]^{3-}$

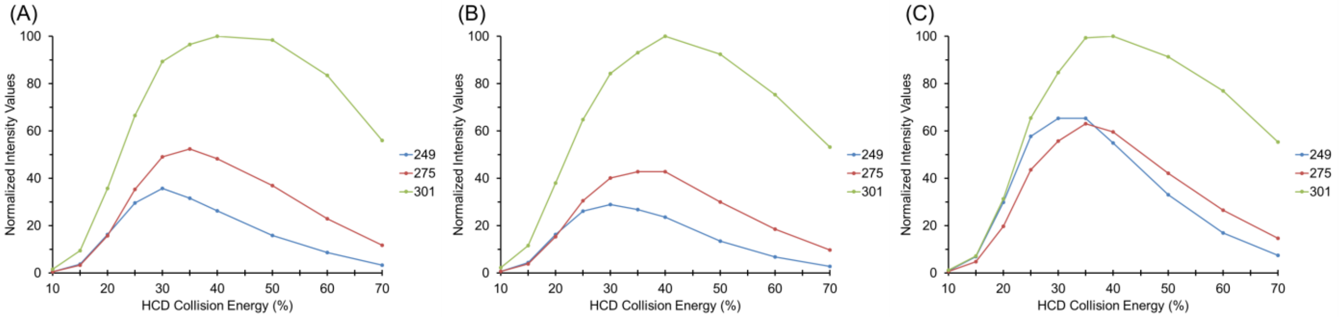

Figure S-9: Normalized abundance of (249, 275, 301) ions with HCD collision energy of: (A) roburin A $[M-3H]^{3-}$, (B) roburin D $[M-3H]^{3-}$, (C) roburin B/C $[M-3H]^{3-}$.

Figure S-10: MS^2 Spectra – CID 20 and HCD 20

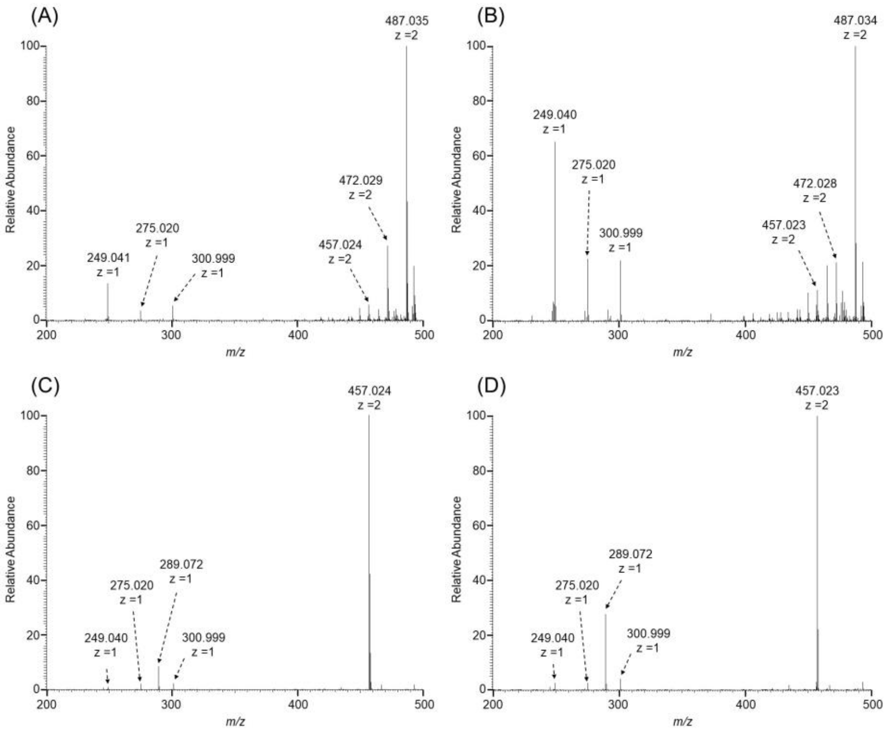

Figure S-10: MS^2 spectra for (A) grandinin CID 20, (B) grandinin HCD 20, (C) acutissimin A CID 20, (D) acutissimin A HCD 20.

Figure S-11: CID Data Points – MS 50K, 50 ms

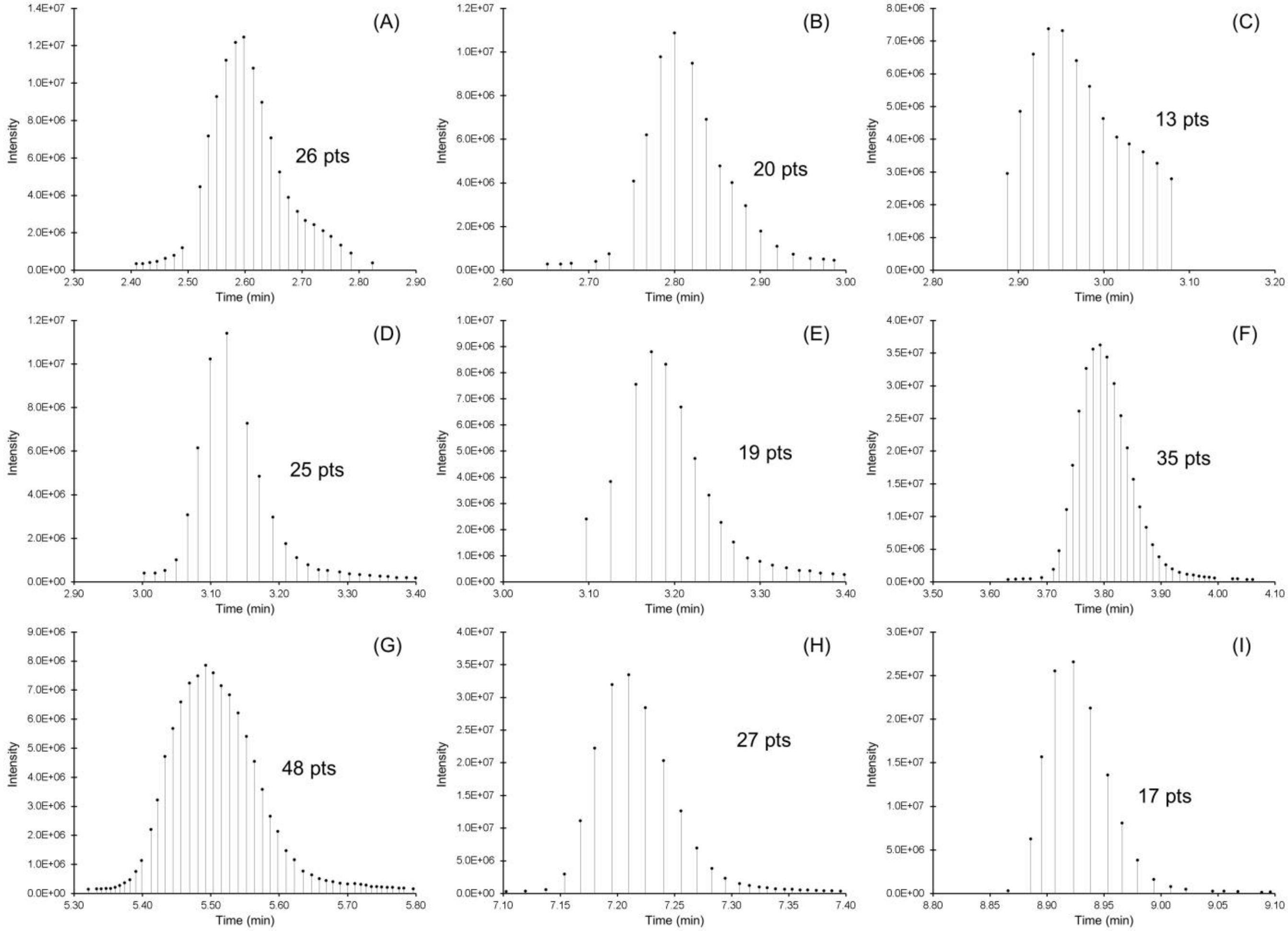

Figure S-11: Discrete CID data points of the $[M-2H]^{2-}$ ion for 40 ng of each ellagitannin acquired with a dianion HCD-CID screen with a maximum injection time of 50 ms at an Orbitrap resolution of 50K for MS spectra: (A) roburin B/C, (B) roburin A, (C) grandinin, (D) roburin D, (E) roburin E, (F) vescalagin, (G) castalagin, (H) acutissimin A, (I) epiacutissimin A.

Figure S-12: CID Data Points – MS 30K, 50 ms

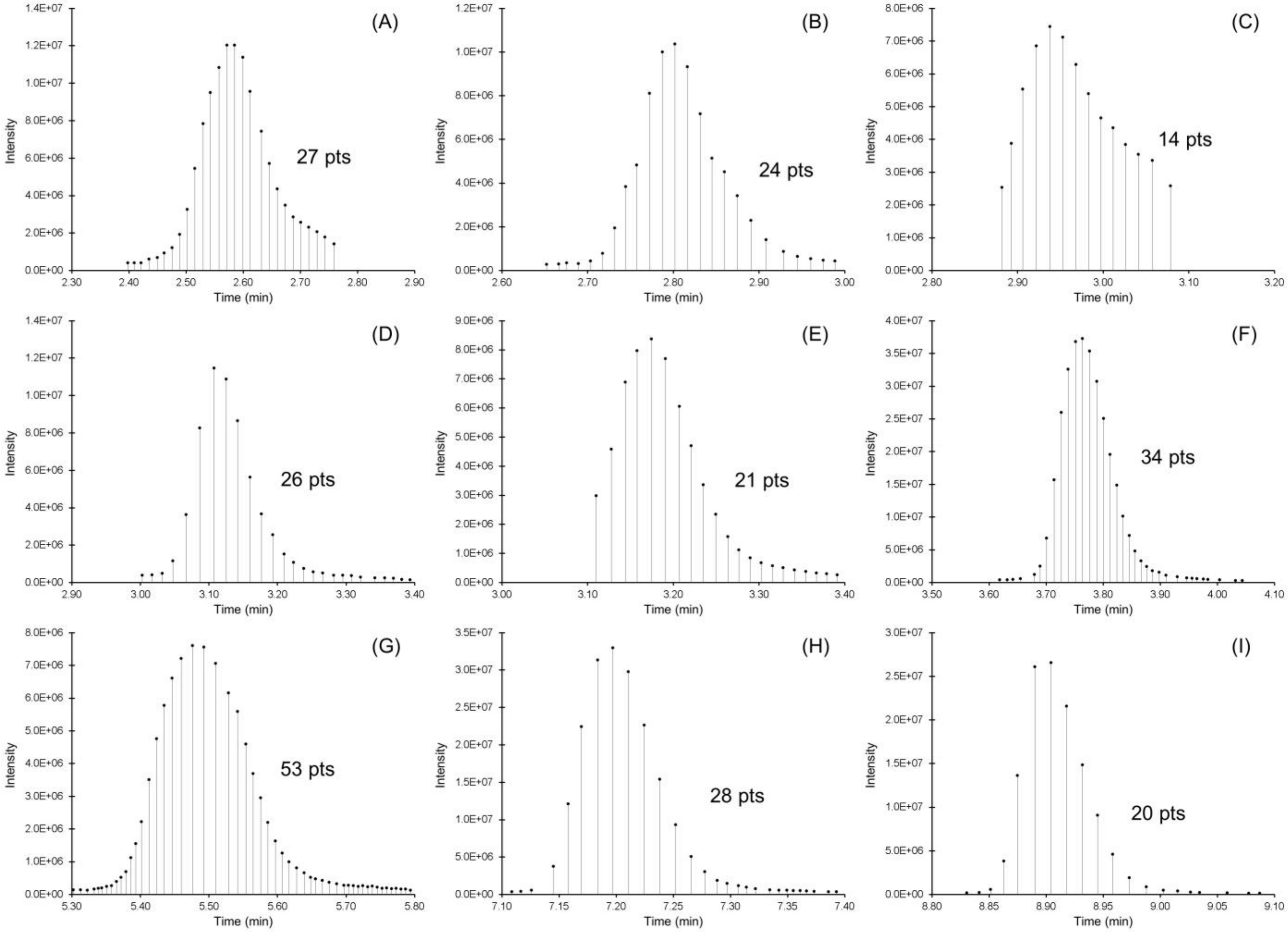

Figure S-12: Discrete CID data points of the $[M-2H]^{2-}$ ion for 40 ng of each ellagitannin acquired with a dianion HCD-CID screen with a maximum injection time of 50 ms at an Orbitrap resolution of 30K for MS spectra: (A) roburin B/C, (B) roburin A, (C) grandinin, (D) roburin D, (E) roburin E, (F) vescalagin, (G) castalagin, (H) acutissimin A, (I) epiacutissimin A.

Figure S-13: CID Data Points – MS 15K, 50 ms

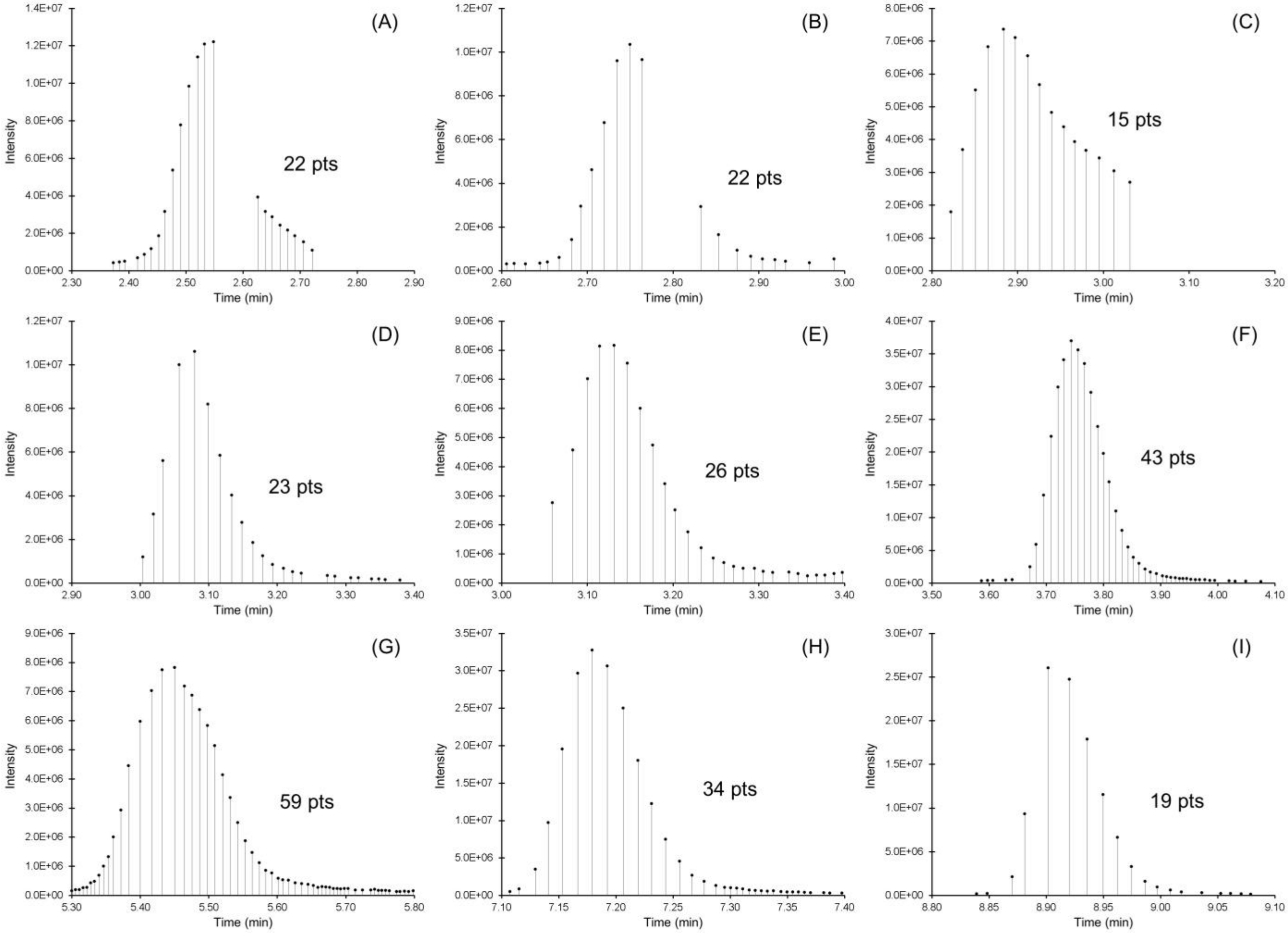

Figure S-13: Discrete CID data points of the $[M-2H]^{2-}$ ion for 40 ng of each ellagitannin acquired with a dianion HCD-CID screen with a maximum injection time of 50 ms at an Orbitrap resolution of 15K for MS spectra: (A) roburin B/C, (B) roburin A, (C) grandinin, (D) roburin D, (E) roburin E, (F) vescalagin, (G) castalagin, (H) acutissimin A, (I) epiacutissimin A.

Figure S-14: CID Data Points – MS 50K, 25 ms

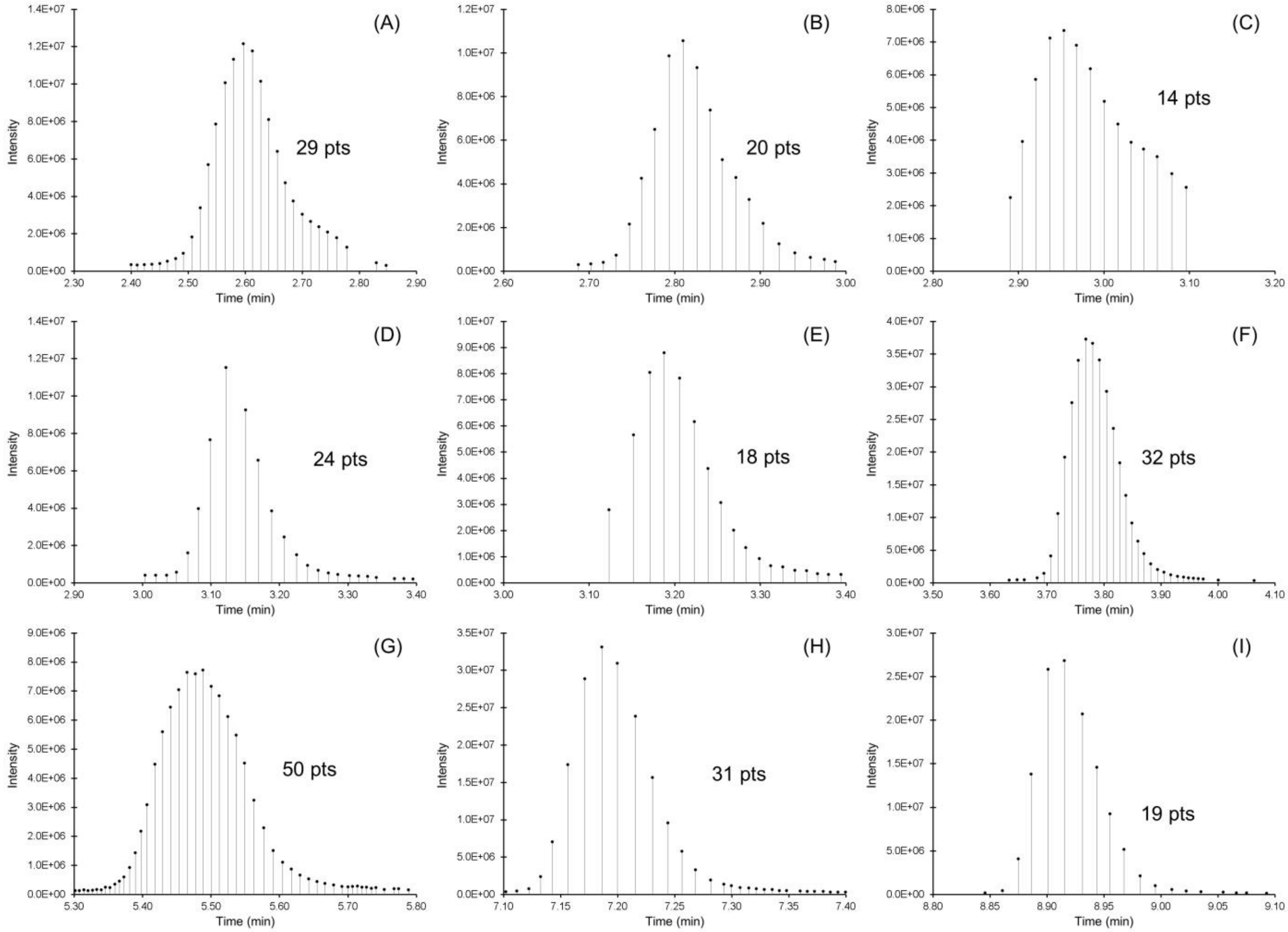

Figure S-14: Discrete CID data points of the $[M-2H]^{2-}$ ion for 40 ng of each ellagitannin acquired with a dianion HCD-CID screen with a maximum injection time of 25 ms at an Orbitrap resolution of 50K for MS spectra: (A) roburin B/C, (B) roburin A, (C) grandinin, (D) roburin D, (E) roburin E, (F) vescalagin, (G) castalagin, (H) acutissimin A, (I) epiacutissimin A.

Figure S-15: CID Data Points – MS 30K, 25 ms

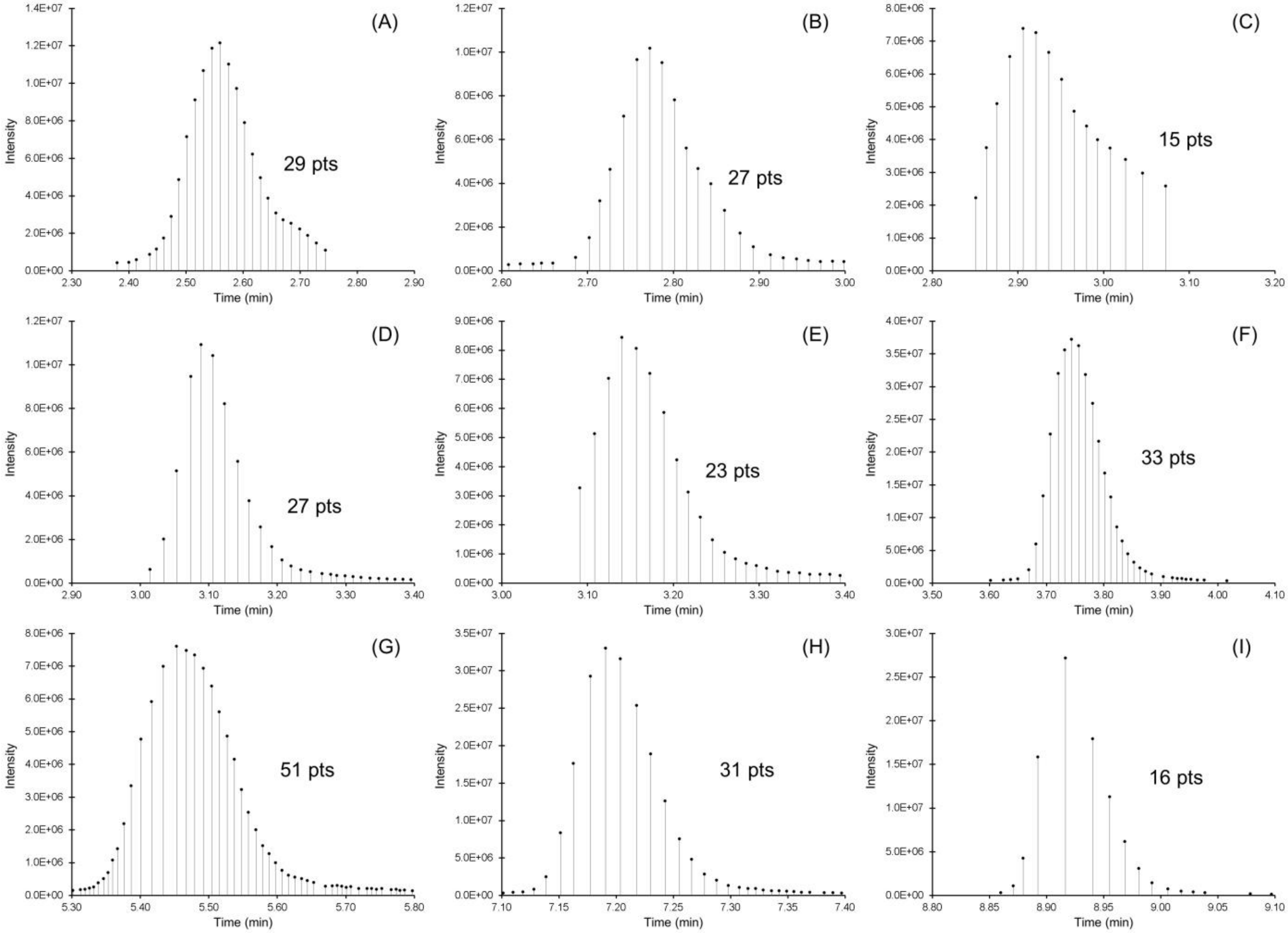

Figure S-15: Discrete CID data points of the $[M-2H]^{2-}$ ion for 40 ng of each ellagitannin acquired with a dianion HCD-CID screen with a maximum injection time of 25 ms at an Orbitrap resolution of 30K for MS spectra: (A) roburin B/C, (B) roburin A, (C) grandinin, (D) roburin D, (E) roburin E, (F) vescalagin, (G) castalagin, (H) acutissimin A, (I) epiacutissimin A.

Figure S-16: CID Data Points – MS 15K, 25 ms

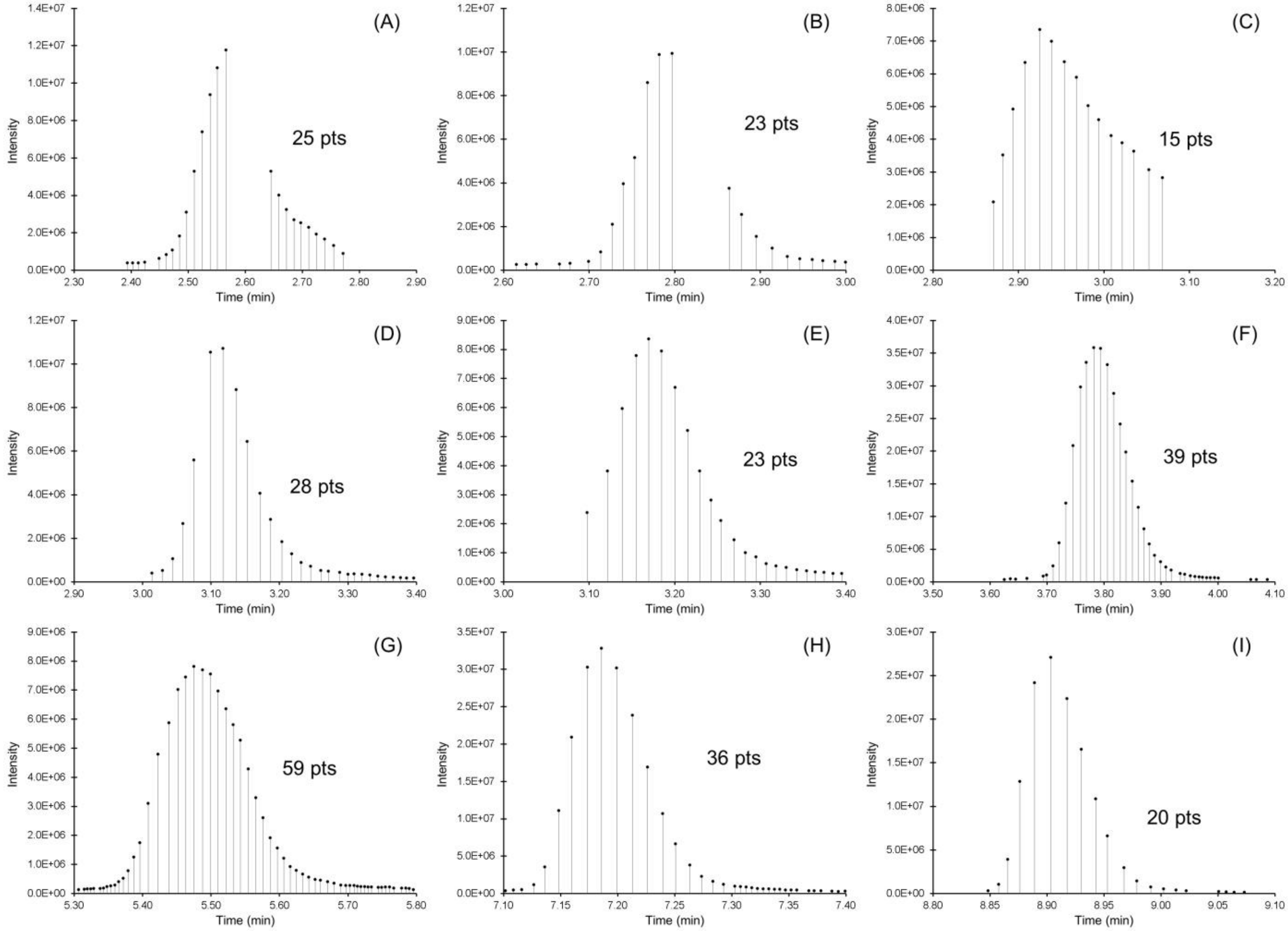

Figure S-16: Discrete CID data points of the $[M-2H]^{2-}$ ion for 40 ng of each ellagitannin acquired with a dianion HCD-CID screen with a maximum injection time of 25 ms at an Orbitrap resolution of 15K for MS spectra: (A) roburin B/C, (B) roburin A, (C) grandinin, (D) roburin D, (E) roburin E, (F) vescalagin, (G) castalagin, (H) acutissimin A, (I) epiacutissimin A.

Figure S-17: CID Data Points – CID 50K, AGC 5e4

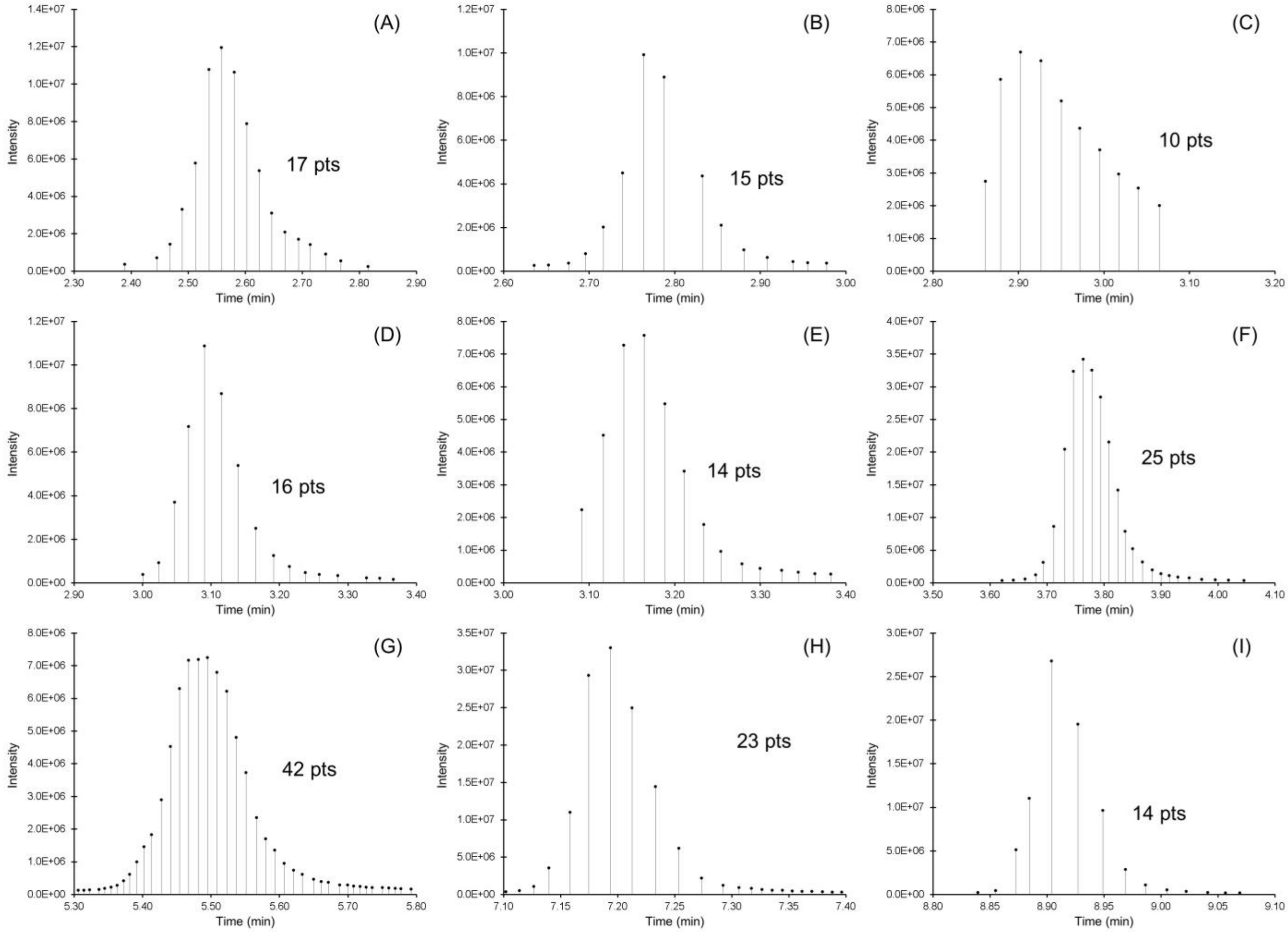

Figure S-17: Discrete CID data points of the $[M-2H]^{2-}$ ion for 40 ng of each ellagitannin acquired with a dianion HCD-CID screen at an AGC setting of 5e4 and Orbitrap resolution of 50K for CID spectra: (A) roburin B/C, (B) roburin A, (C) grandinin, (D) roburin D, (E) roburin E, (F) vescalagin, (G) castalagin, (H) acutissimin A, (I) epiacutissimin A.

Figure S-18: CID Data Points – CID 30K, AGC 5e4

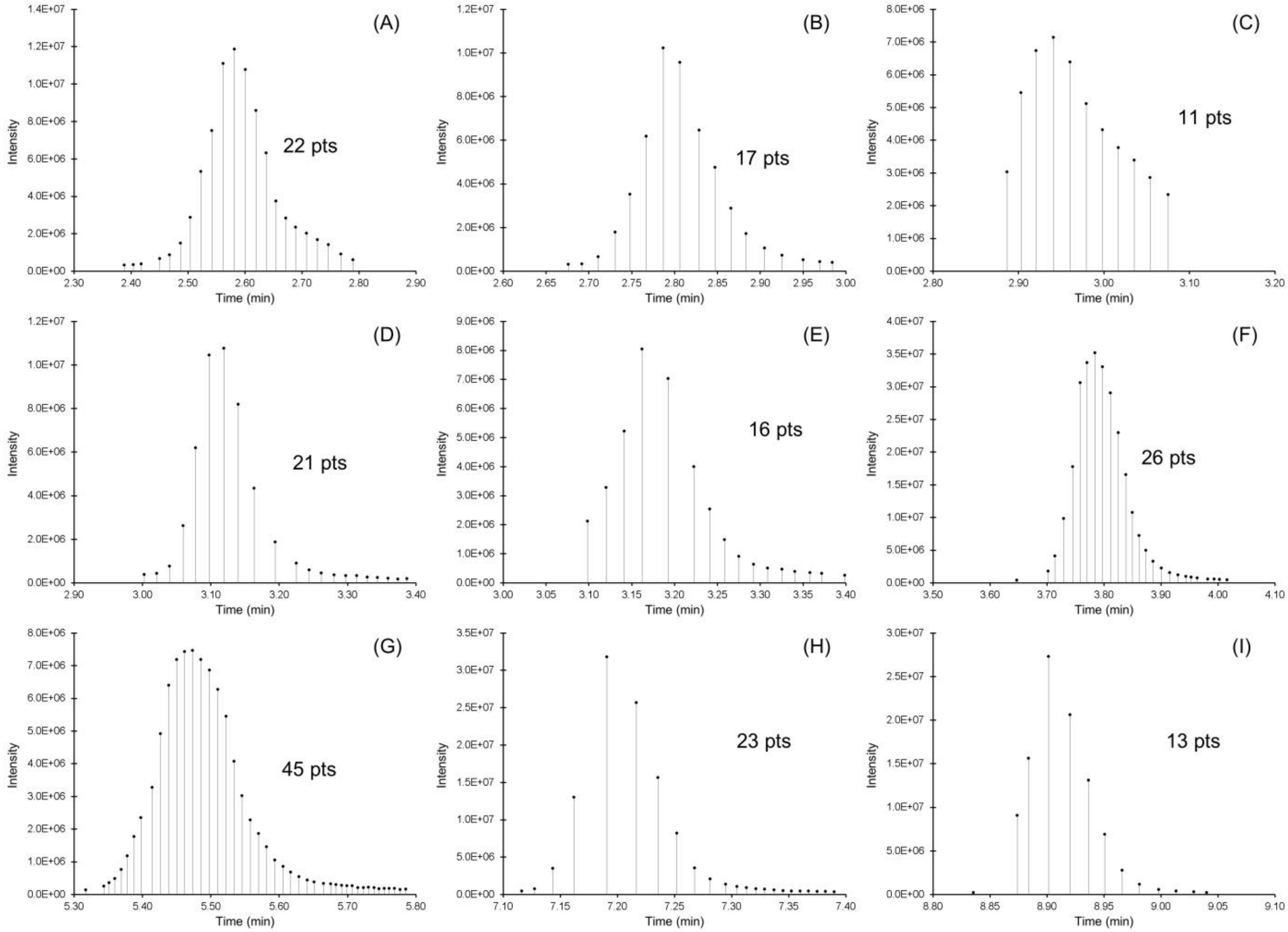

Figure S-18: Discrete CID data points of the $[M-2H]^{2-}$ ion for 40 ng of each ellagitannin acquired with a dianion HCD-CID screen at an AGC setting of 5e4 and Orbitrap resolution of 30K for CID spectra: (A) roburin B/C, (B) roburin A, (C) grandinin, (D) roburin D, (E) roburin E, (F) vescalagin, (G) castalagin, (H) acutissimin A, (I) epiacutissimin A.

Figure S-19: CID Data Points – CID 15K, AGC 5e4

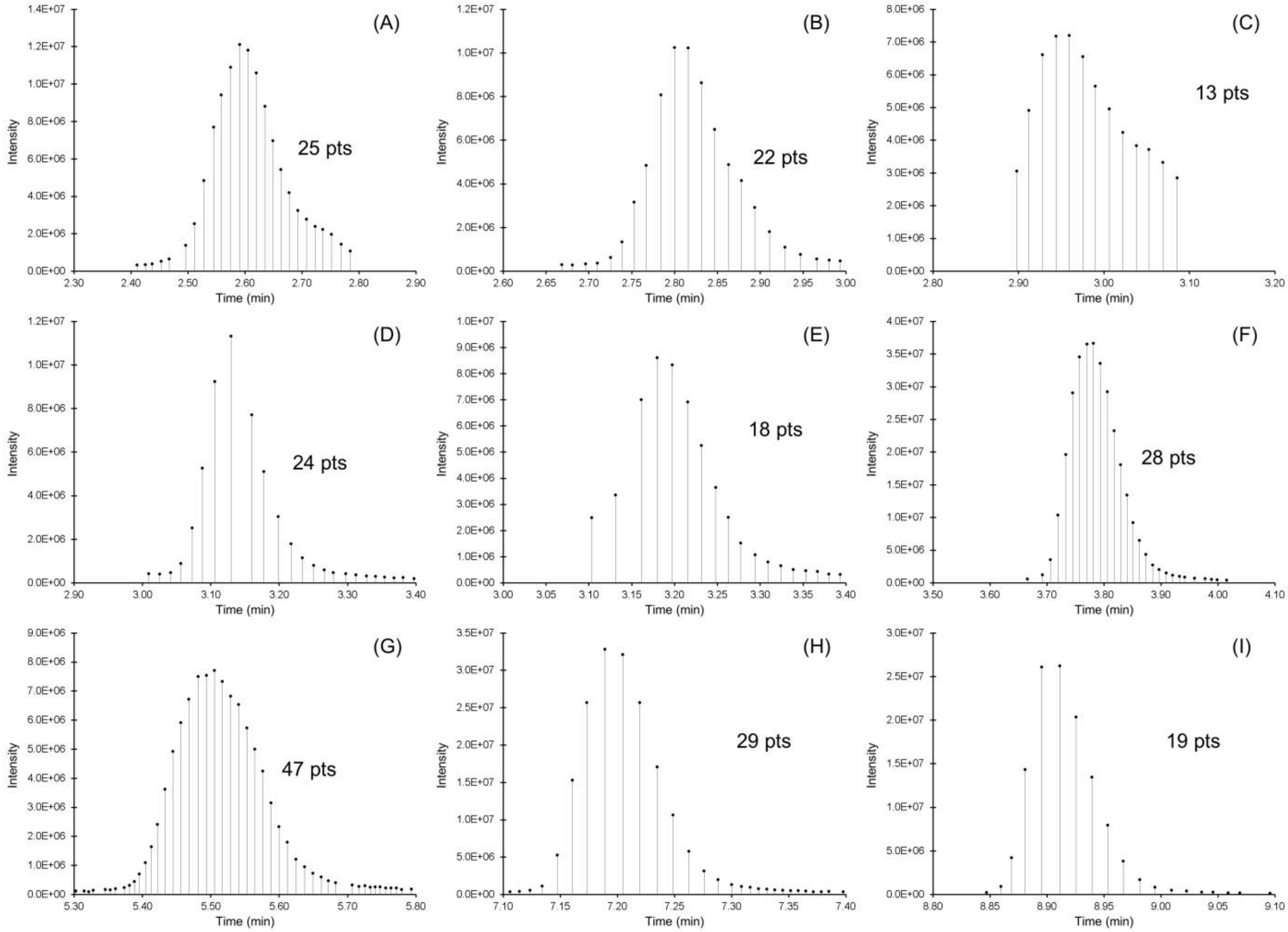

<u>Figure S-19</u>: Discrete CID data points of the $[M-2H]^{2-}$ ion for 40 ng of each ellagitannin acquired with a dianion HCD-CID screen at an AGC setting of 5e4 and Orbitrap resolution of 15K for CID spectra: (A) roburin B/C, (B) roburin A, (C) grandinin, (D) roburin D, (E) roburin E, (F) vescalagin, (G) castalagin, (H) acutissimin A, (I) epiacutissimin A.

Figure S-20: CID Data Points – CID 50K, AGC 1e4

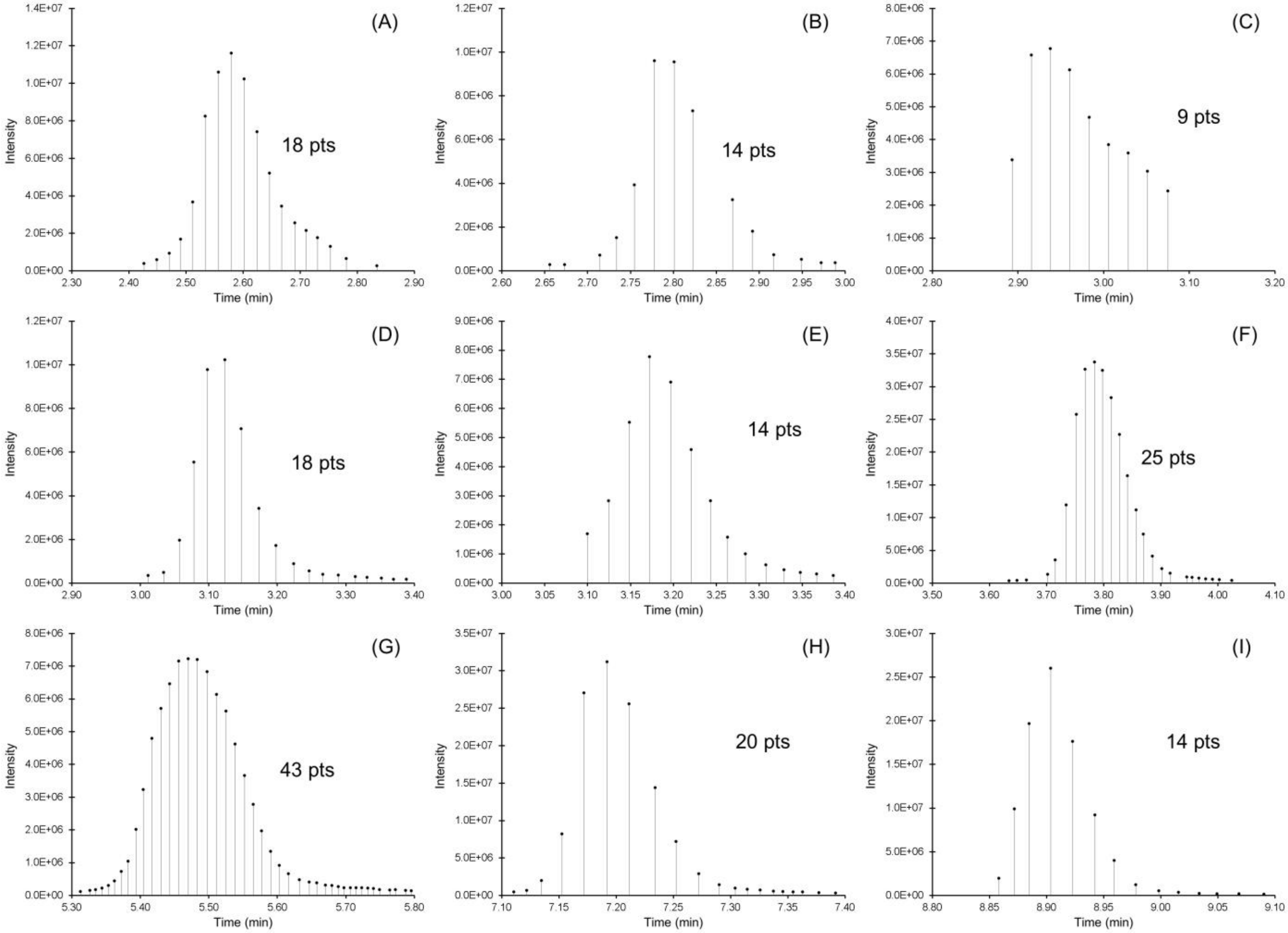

Figure S-20: Discrete CID data points of the $[M-2H]^{2-}$ ion for 40 ng of each ellagitannin acquired with a dianion HCD-CID screen at an AGC setting of 1e4 and Orbitrap resolution of 50K for CID spectra: (A) roburin B/C, (B) roburin A, (C) grandinin, (D) roburin D, (E) roburin E, (F) vescalagin, (G) castalagin, (H) acutissimin A, (I) epiacutissimin A.

Figure S-21: CID Data Points – CID 30K, AGC 1e4

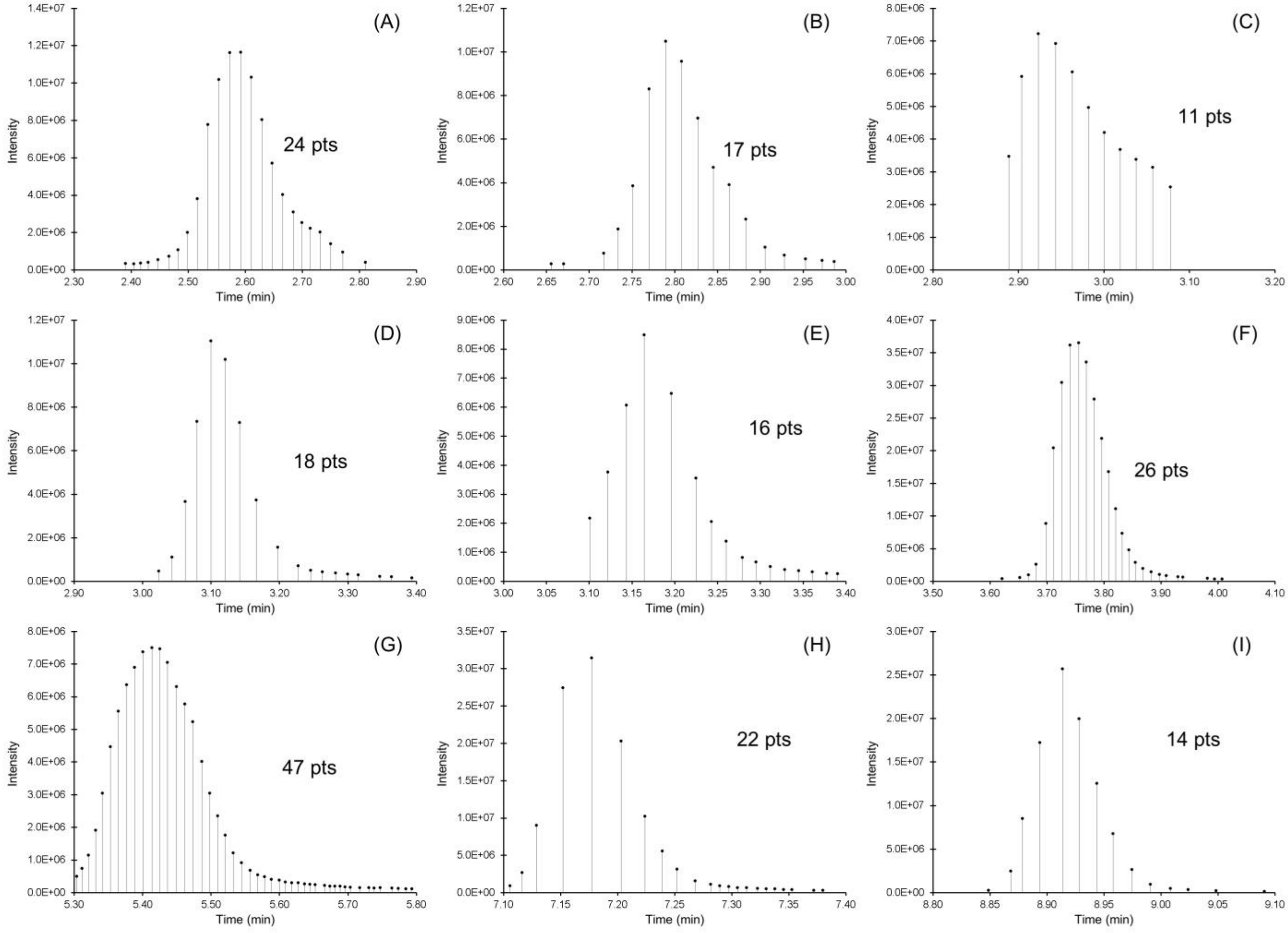

Figure S-21: Discrete CID data points of the $[M-2H]^{2-}$ ion for 40 ng of each ellagitannin acquired with a dianion HCD-CID screen at an AGC setting of 1e4 and Orbitrap resolution of 30K for CID spectra: (A) roburin B/C, (B) roburin A, (C) grandinin, (D) roburin D, (E) roburin E, (F) vescalagin, (G) castalagin, (H) acutissimin A, (I) epiacutissimin A.

Figure S-22: CID Data Points – CID 15K, AGC 1e4

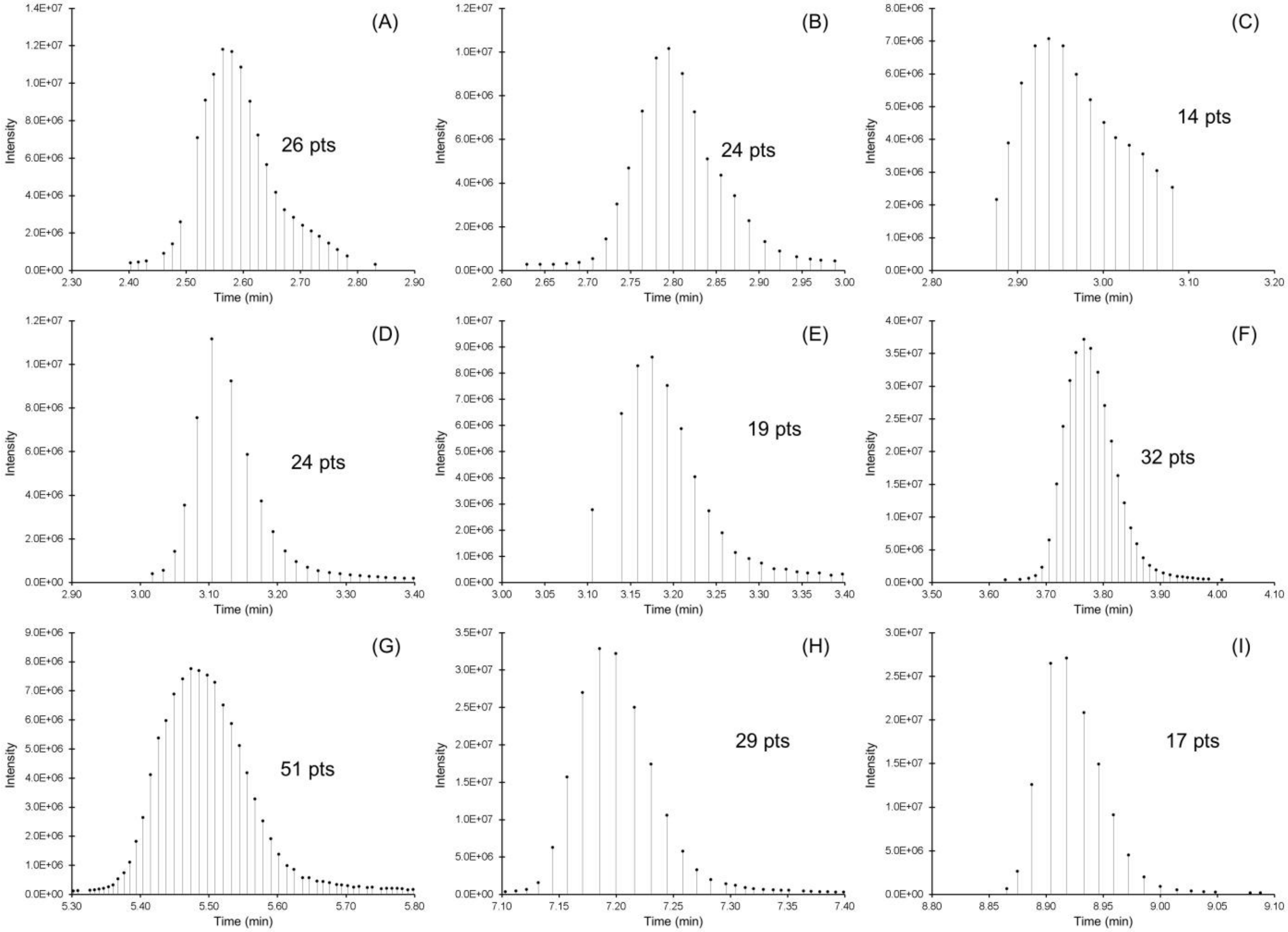

Figure S-22: Discrete CID data points of the $[M-2H]^{2-}$ ion for 40 ng of each ellagitannin acquired with a dianion HCD-CID screen at an AGC setting of 1e4 and Orbitrap resolution of 15K for CID spectra: (A) roburin B/C, (B) roburin A, (C) grandinin, (D) roburin D, (E) roburin E, (F) vescalagin, (G) castalagin, (H) acutissimin A, (I) epiacutissimin A.

Figure S-23: CID Data Points – IT Normal, AGC 1e4

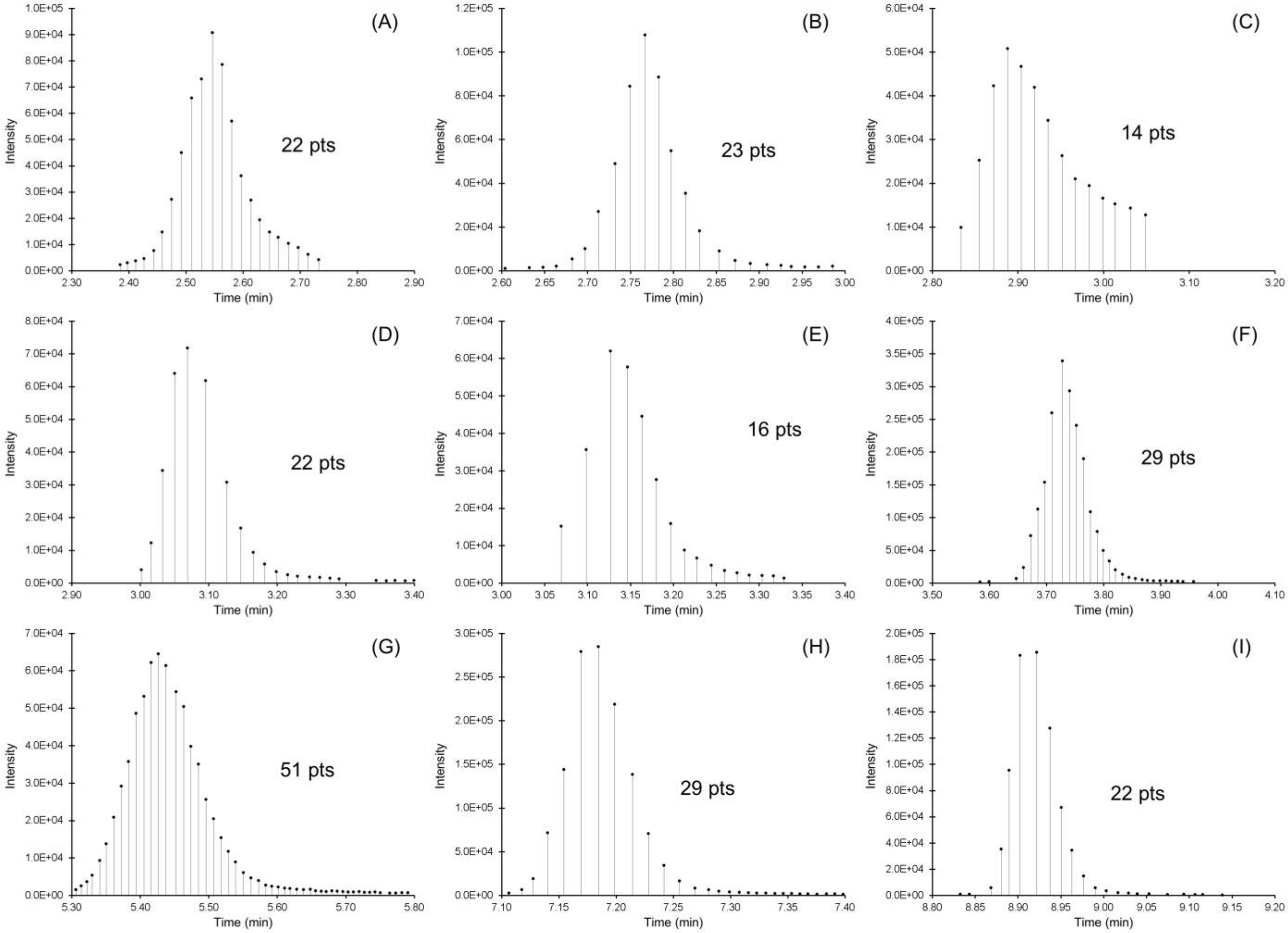

Figure S-23: Discrete CID data points of the $[M-2H]^{2-}$ ion for 40 ng of each ellagitannin acquired with a dianion HCD-CID screen at an AGC setting of 1e4 and Ion Trap scan rate of Normal for CID spectra: (A) roburin B/C, (B) roburin A, (C) grandinin, (D) roburin D, (E) roburin E, (F) vescalagin, (G) castalagin, (H) acutissimin A, (I) epiacutissimin A.

Figure S-24: CID Data Points – IT Rapid, AGC 1e4

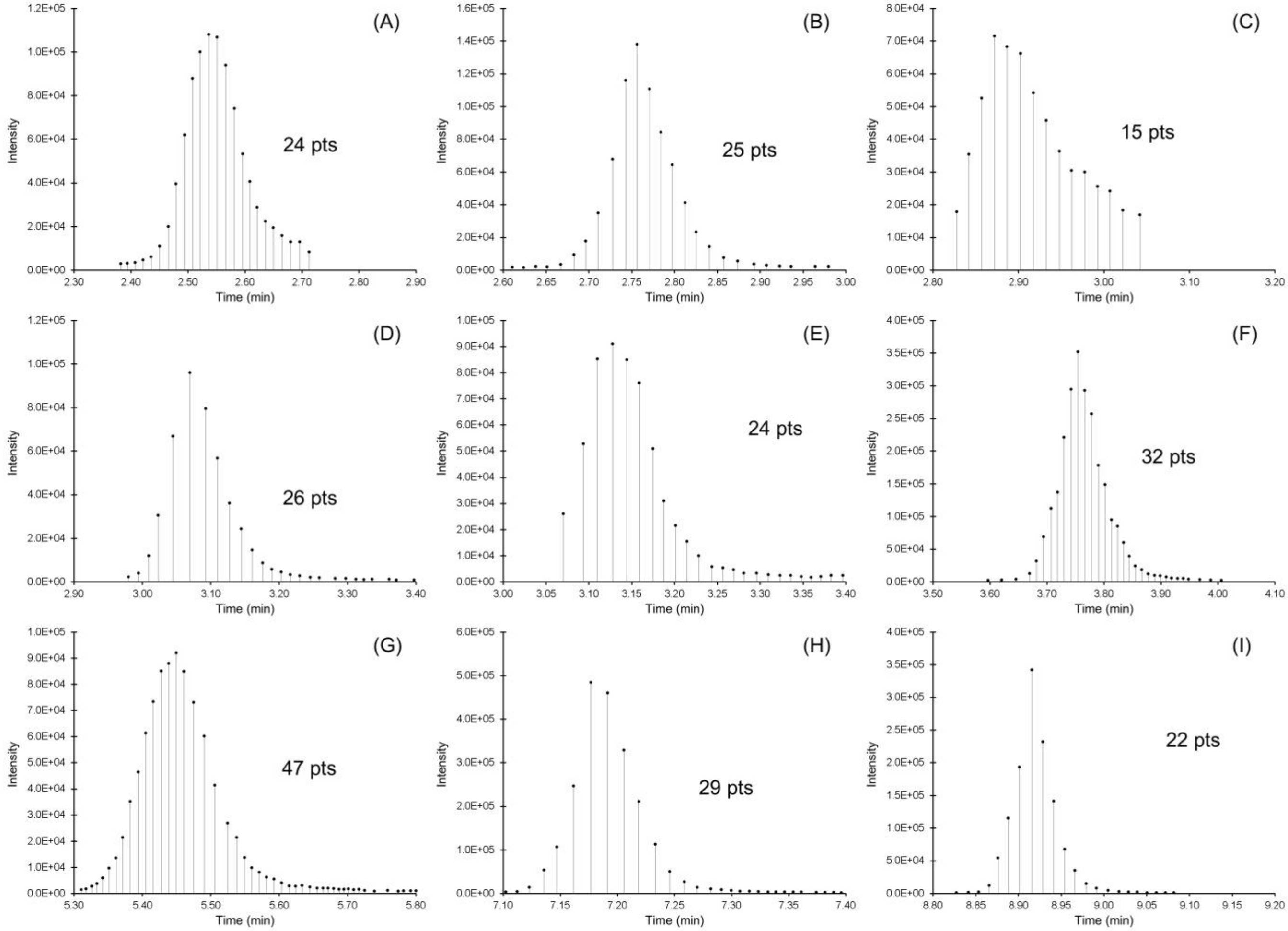

Figure S-24: Discrete CID data points of the $[M-2H]^{2-}$ ion for 40 ng of each ellagitannin acquired with a dianion HCD-CID screen at an AGC setting of 1e4 and Ion Trap scan rate of Rapid for CID spectra: (A) roburin B/C, (B) roburin A, (C) grandinin, (D) roburin D, (E) roburin E, (F) vescalagin, (G) castalagin, (H) acutissimin A, (I) epiacutissimin A.

Figure S-25: CID Data Points – IT Turbo, AGC 1e4

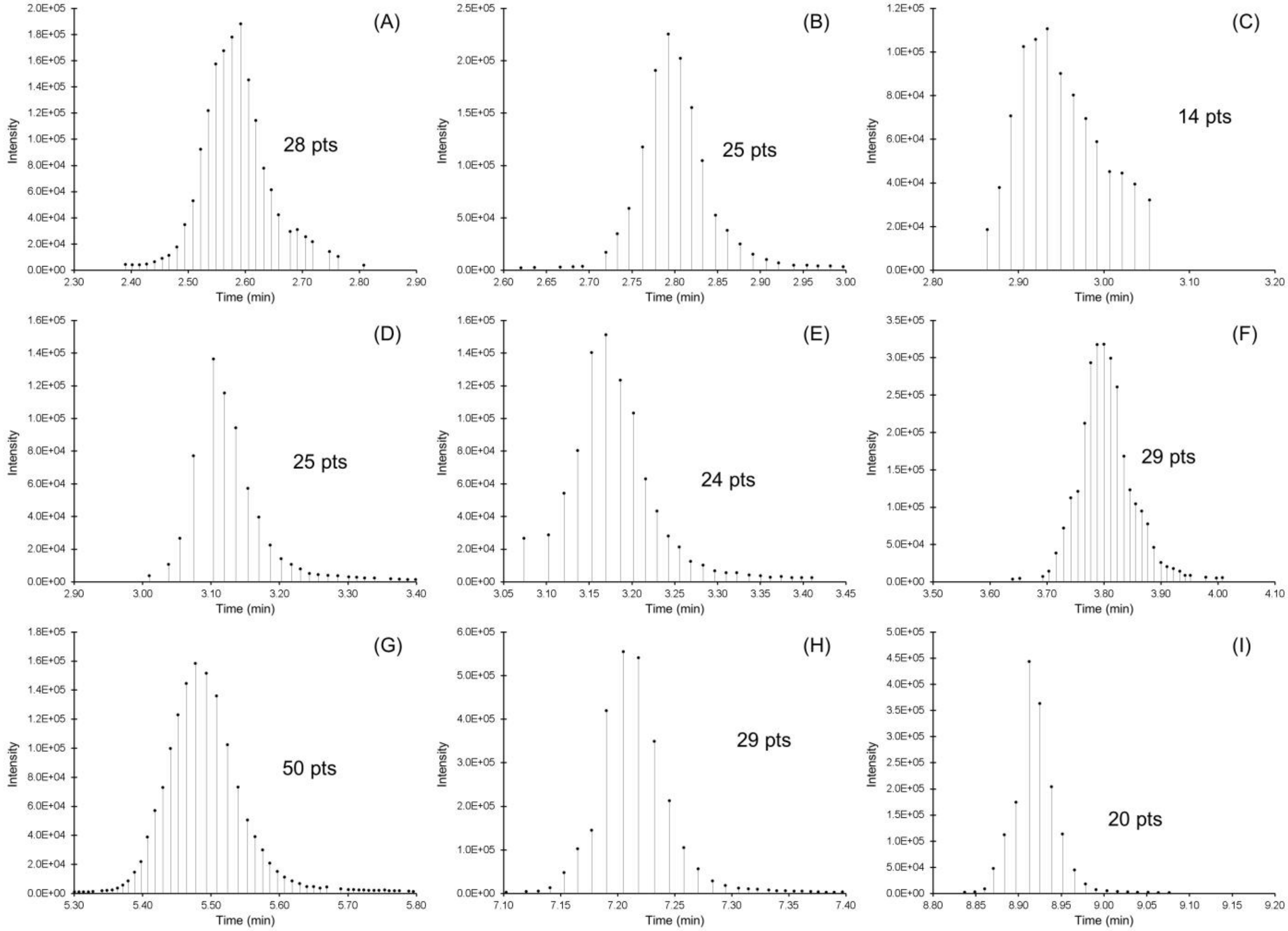

<u>Figure S-25</u>: Discrete CID data points of the $[M-2H]^{2-}$ ion for 40 ng of each ellagitannin acquired with a dianion HCD-CID screen at an AGC setting of 1e4 and Ion Trap scan rate of Turbo for CID spectra: (A) roburin B/C, (B) roburin A, (C) grandinin, (D) roburin D, (E) roburin E, (F) vescalagin, (G) castalagin, (H) acutissimin A, (I) epiacutissimin A.

Figure S-26: CID Spectra – Orbitrap vs Ion Trap

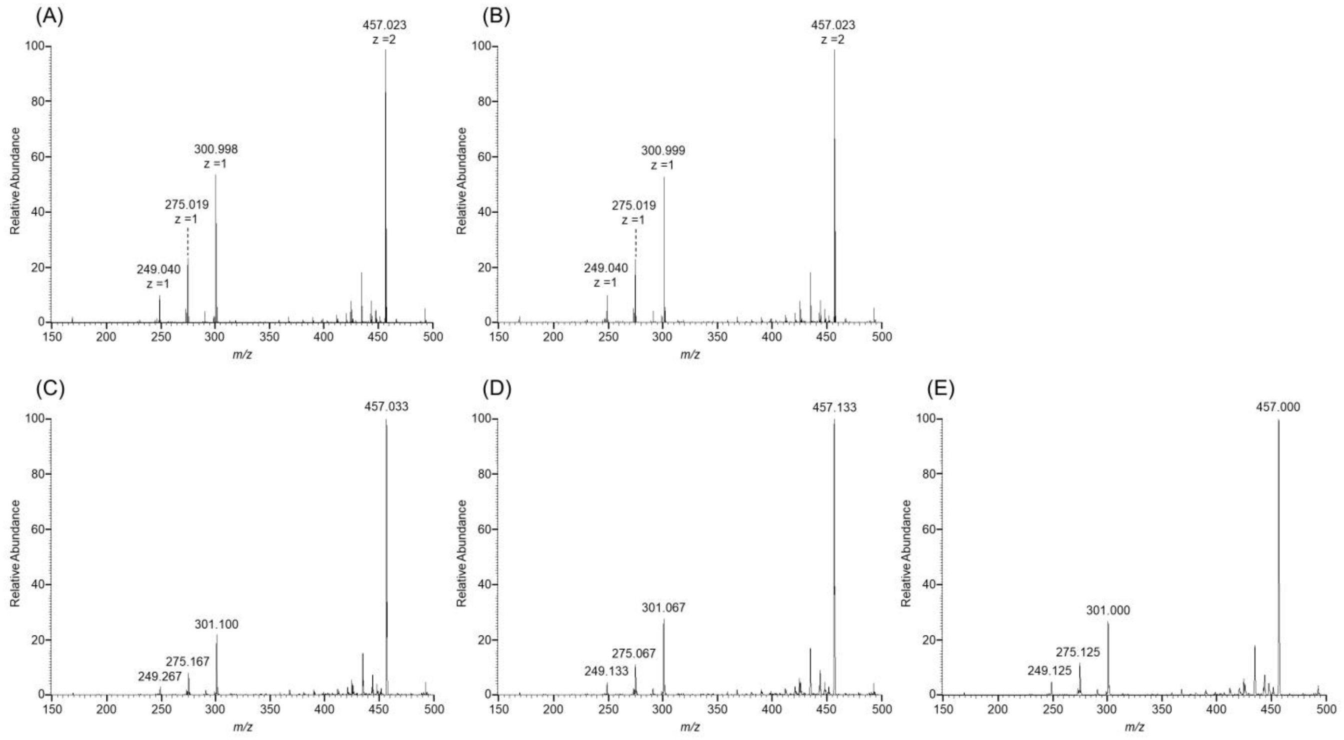

Figure S-26: CID spectra of castalagin acquired via a dianion HCD-CID screen with varied CID scan conditions of: (A) Orbitrap - 15K, 5e4 AGC, (B) Orbitrap - 15K, 1e4 AGC, (C) Ion Trap - Normal, 1e4 AGC, (D) Ion Trap - Rapid, 1e4 AGC, (E) Ion Trap - Turbo, 1e4 AGC.

Figure S-27: CID EICs – Standards, 40 ng to 1.25 ng

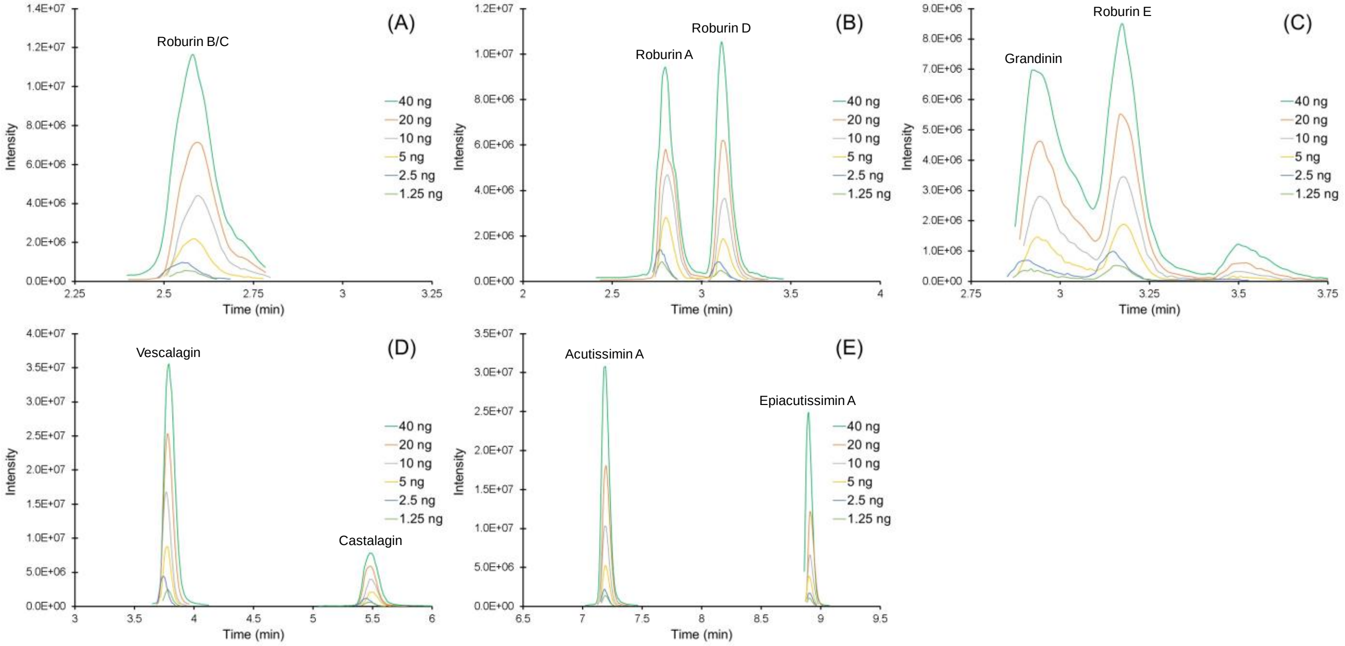

Figure S-27: CID EICs for dilutions of: (A) roburin B/C $[M\text{-}2H]^{2-}$, (B) roburin A $[M\text{-}2H]^{2-}$ & roburin D $[M\text{-}2H]^{2-}$, (C) grandinin $[M\text{-}2H]^{2-}$ & roburin E $[M\text{-}2H]^{2-}$, (D) vescalagin $[M\text{-}2H]^{2-}$ & castalagin $[M\text{-}2H]^{2-}$, (E) acutissimin A $[M\text{-}2H]^{2-}$ & epiacutissimin A $[M\text{-}2H]^{2-}$.

Figure S-28: CID Data Points – Standards, 5 ng

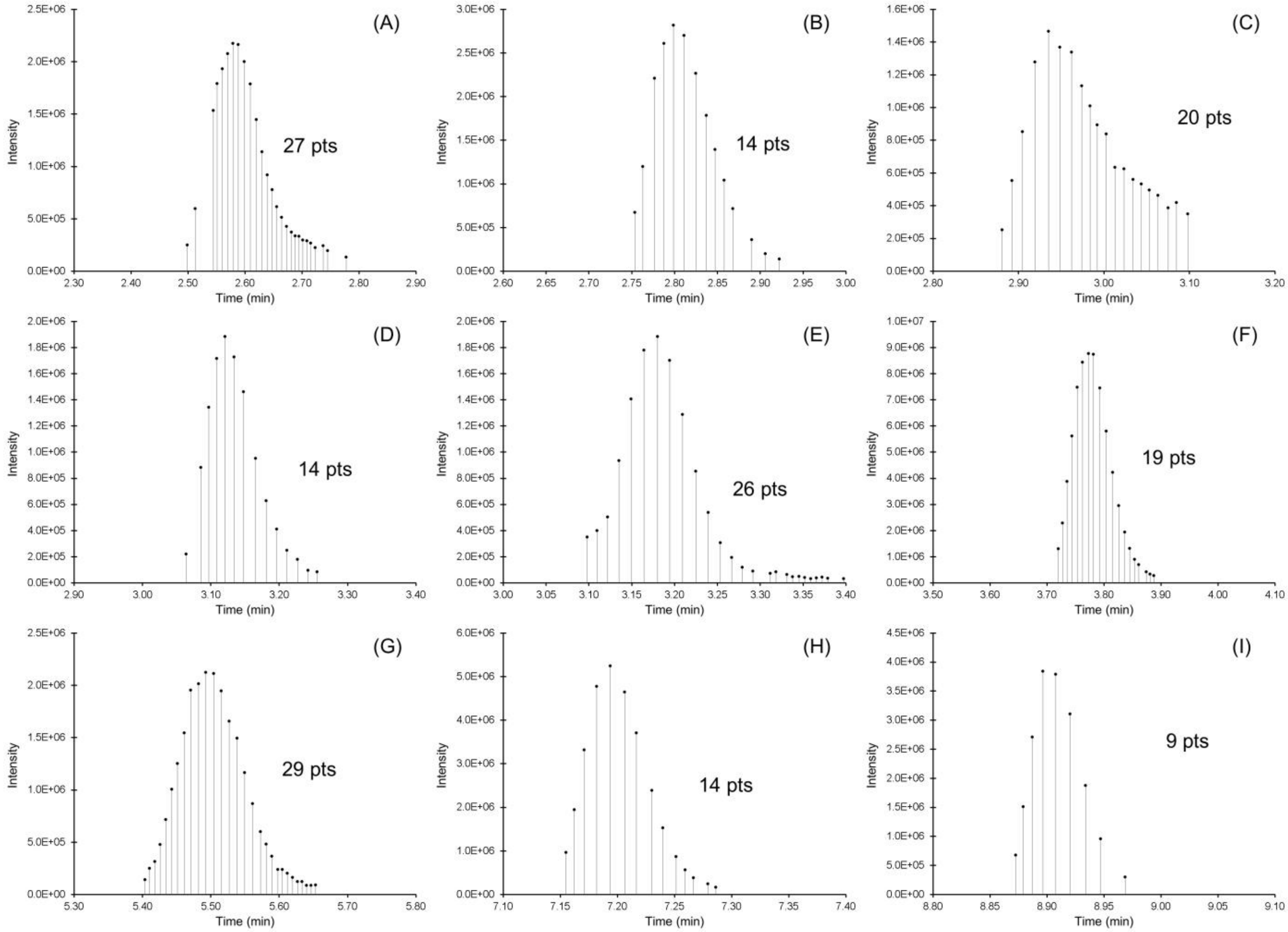

Figure S-28: Discrete CID data points for 5 ng of each ellagitannin $[M\text{-}2H]^{2-}$ acquired with a dianion HCD-CID screen: (A) roburin B/C, (B) roburin A, (C) grandinin, (D) roburin D, (E) roburin E, (F) vescalagin, (G) castalagin, (H) acutissimin A, (I) epiacutissimin A.

Figure S-29: CID Data Points – Standards, 2.5 ng

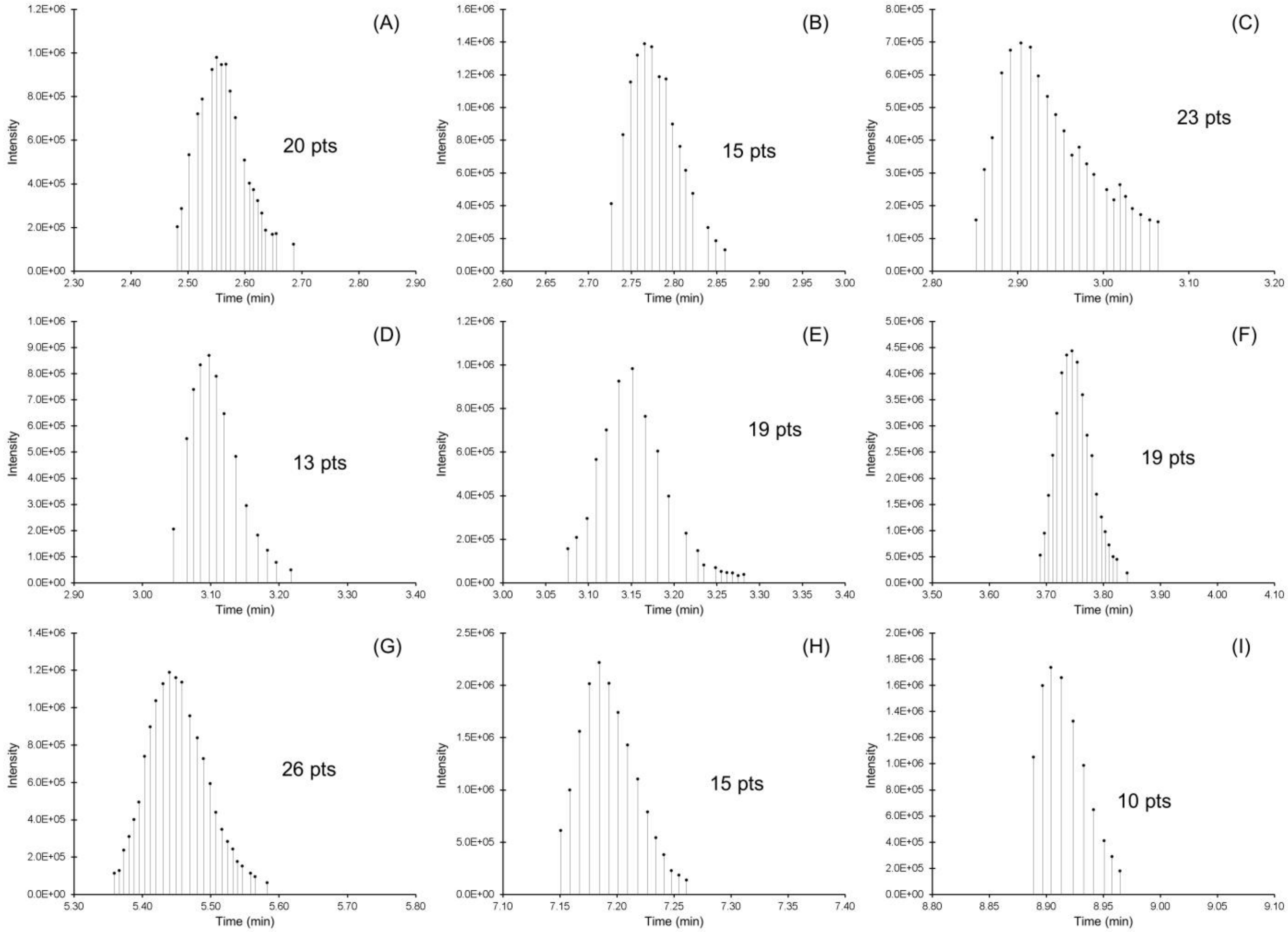

<u>Figure S-29</u>: Discrete CID data points for 2.5 ng of each ellagitannin $[M\text{-}2H]^{2-}$ acquired with a dianion HCD-CID screen: (A) roburin B/C, (B) roburin A, (C) grandinin, (D) roburin D, (E) roburin E, (F) vescalagin, (G) castalagin, (H) acutissimin A, (I) epiacutissimin A.

Figure S-30: CID Data Points – Standards, 1.25 ng

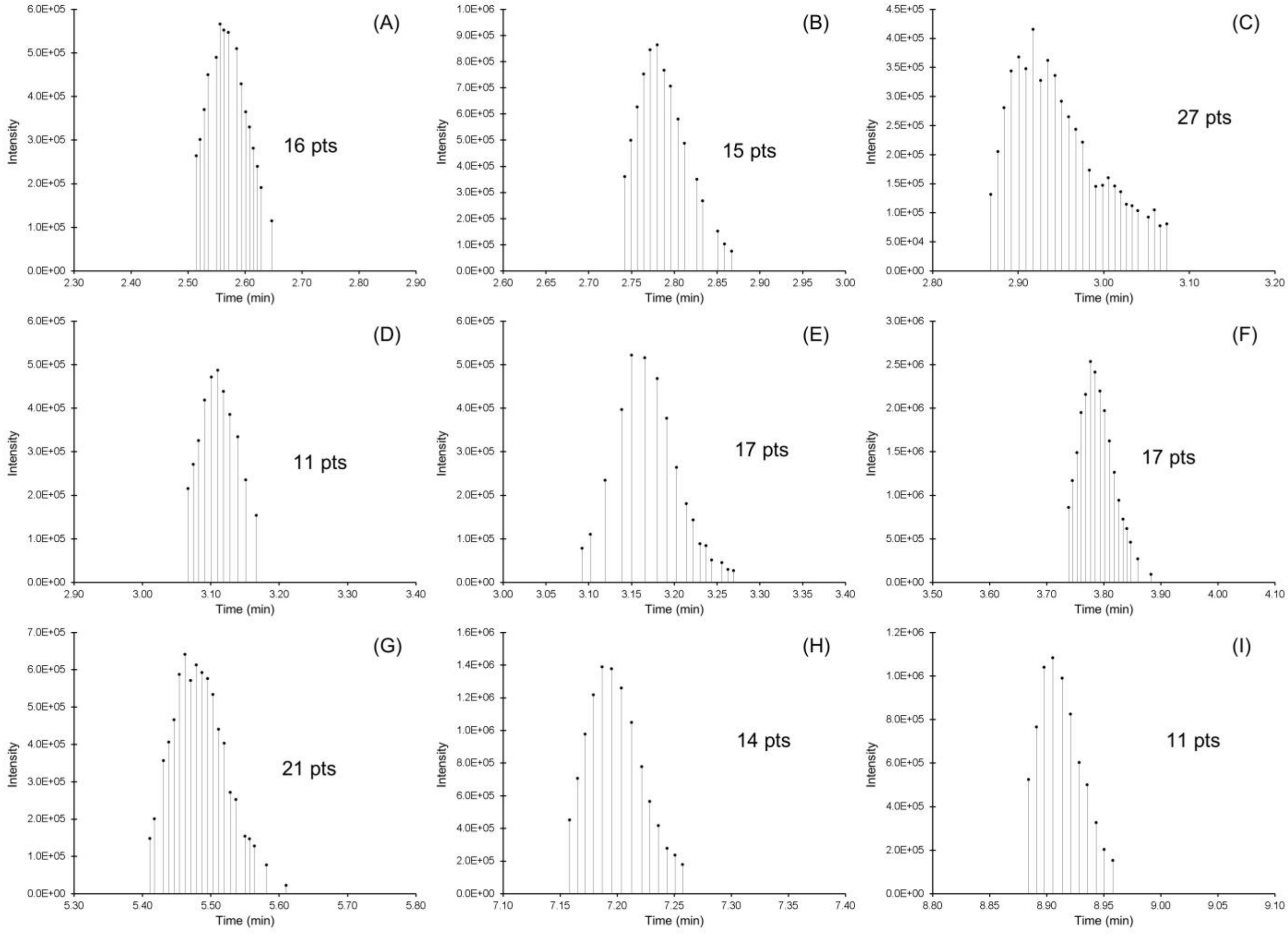

Figure S-30: Discrete CID data points for 1.25 ng of each ellagitannin $[M-2H]^{2-}$ acquired with a dianion HCD-CID screen: (A) roburin B/C, (B) roburin A, (C) grandinin, (D) roburin D, (E) roburin E, (F) vescalagin, (G) castalagin, (H) acutissimin A, (I) epiacutissimin A.

Figure S-31: CID Data Points – Single Isomeric Injections

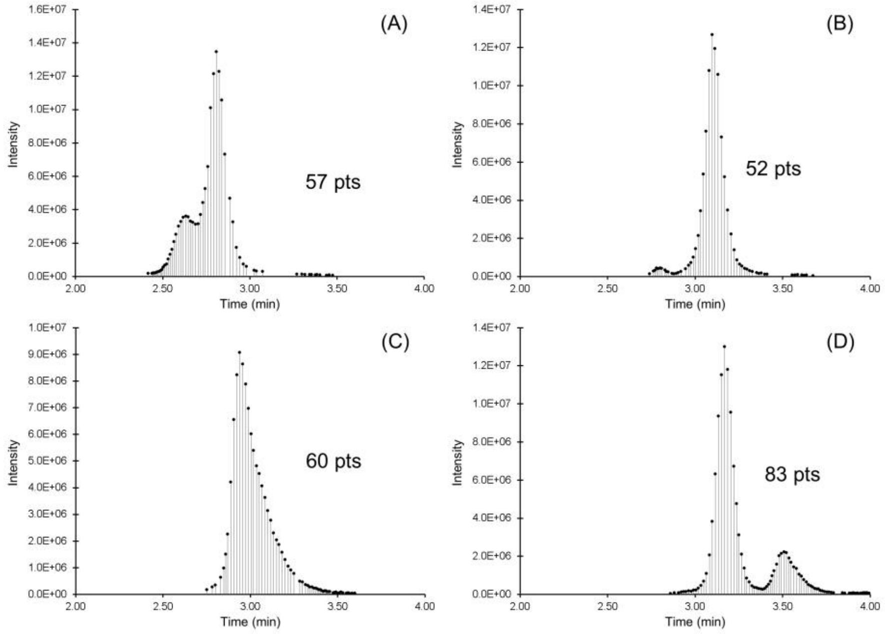

Figure S-31: Discrete CID data points for singular isomeric injections of 40 ng of the closest eluting isomers: (A) roburin A $[M-2H]^{2-}$, (B) roburin D $[M-2H]^{2-}$, (C) grandinin $[M-2H]^{2-}$, (D) roburin E $[M-2H]^{2-}$.

Figure S-32: Mixture Specificity, T-3RT

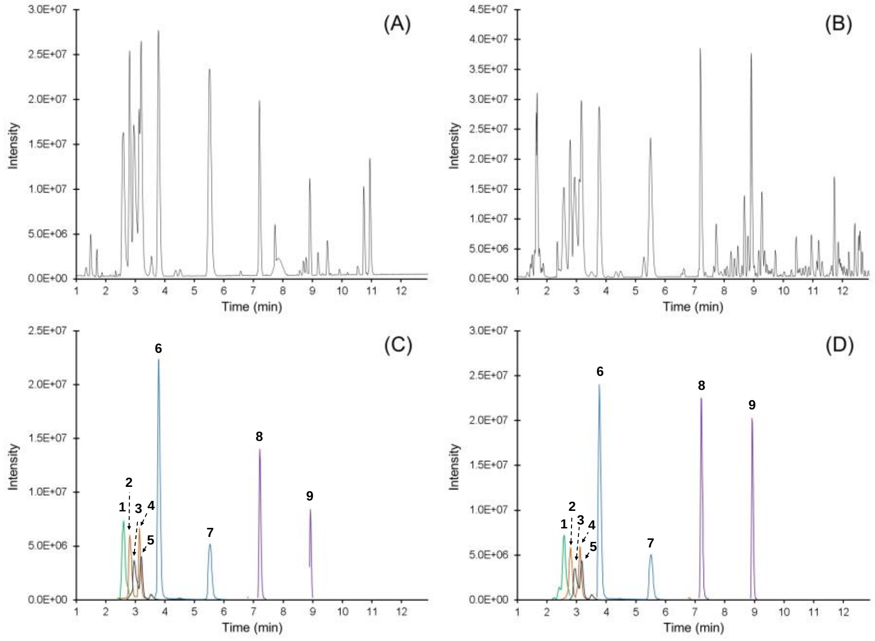

Figure S-32: Application of a dianion HCD-CID screen with T-3RT. MS chromatogram of: (A) 10 µg/mL of ellagitannin standards, (B) Mixture of 100 mg/mL of amaranth and 10 µg/mL of ellagitannin standards. CID EIC of each ellagitannin standard for: (C) 10 µg/mL ellagitannin standards, (D) 100 mg/mL of amaranth and 10 µg/mL of ellagitannin standards. Annotation of standards in (C) and (D): 1. roburin B/C $[M-2H]^{2-}$ green trace, 2. roburin A $[M-2H]^{2-}$ orange trace, 3. grandinin $[M-2H]^{2-}$ black trace, 4. roburin D $[M-2H]^{2-}$ orange trace, 5. roburin E $[M-2H]^{2-}$ black trace, 6. vescalagin $[M-2H]^{2-}$ blue trace, 7. castalagin $[M-2H]^{2-}$, blue trace, 8. acutissimin A $[M-2H]^{2-}$ purple trace, 9. epiacutissimin A $[M-2H]^{2-}$ purple trace.

Figure S-33: Mixture Specificity, T-2RT

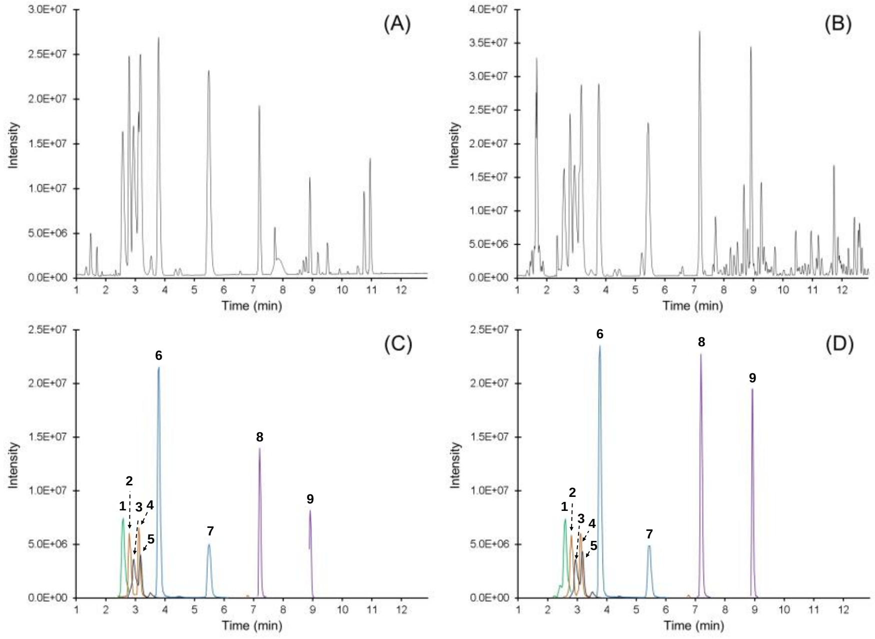

Figure S-33: Application of a dianion HCD-CID screen with T-2RT. MS chromatogram of: (A) 10 µg/mL of ellagitannin standards, (B) Mixture of 100 mg/mL of amaranth and 10 µg/mL of ellagitannin standards. CID EIC of each ellagitannin standard for: (C) 10 µg/mL ellagitannin standards, (D) 100 mg/mL of amaranth and 10 µg/mL of ellagitannin standards. Annotation of standards in (C) and (D): 1. roburin B/C $[M-2H]^{2-}$ green trace, 2. roburin A $[M-2H]^{2-}$ orange trace, 3. grandinin $[M-2H]^{2-}$ black trace, 4. roburin D $[M-2H]^{2-}$ orange trace, 5. roburin E $[M-2H]^{2-}$ black trace, 6. vescalagin $[M-2H]^{2-}$ blue trace, 7. castalagin $[M-2H]^{2-}$, blue trace, 8. acutissimin A $[M-2H]^{2-}$ purple trace, 9. epiacutissimin A $[M-2H]^{2-}$ purple trace.

Figure S-34: Mixture Specificity, T-1RT

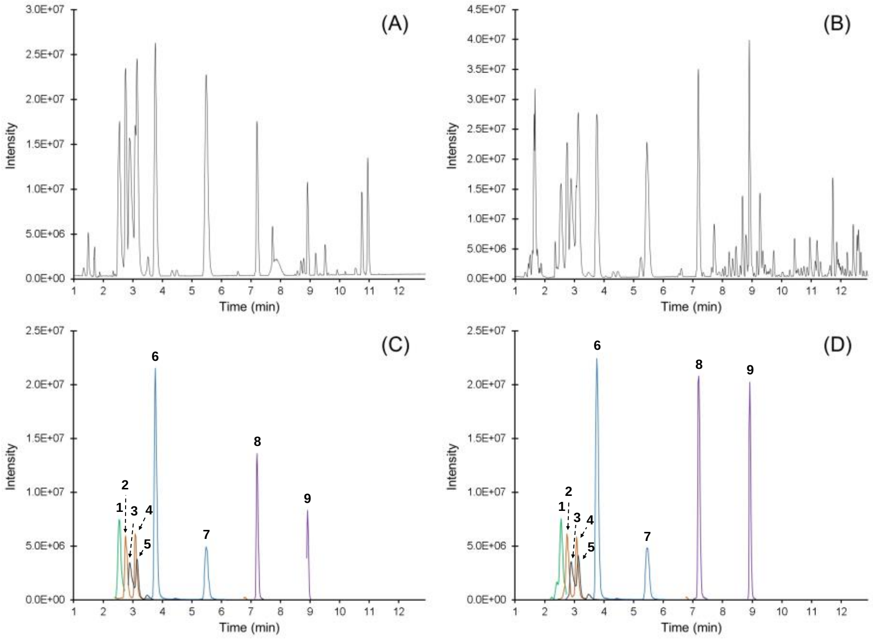

Figure S-34: Application of a dianion HCD-CID screen with T-1RT. MS chromatogram of: (A) 10 µg/mL of ellagitannin standards, (B) Mixture of 100 mg/mL of amaranth and 10 µg/mL of ellagitannin standards. CID EIC of each ellagitannin standard for: (C) 10 µg/mL ellagitannin standards, (D) 100 mg/mL of amaranth and 10 µg/mL of ellagitannin standards. Annotation of standards in (C) and (D): 1. roburin B/C $[M-2H]^{2-}$ green trace, 2. roburin A $[M-2H]^{2-}$ orange trace, 3. grandinin $[M-2H]^{2-}$ black trace, 4. roburin D $[M-2H]^{2-}$ orange trace, 5. roburin E $[M-2H]^{2-}$ black trace, 6. vescalagin $[M-2H]^{2-}$ blue trace, 7. castalagin $[M-2H]^{2-}$, blue trace, 8. acutissimin A $[M-2H]^{2-}$ purple trace, 9. epiacutissimin A $[M-2H]^{2-}$ purple trace.

Figure S-35: Mass Trigger Controls, T-3RT

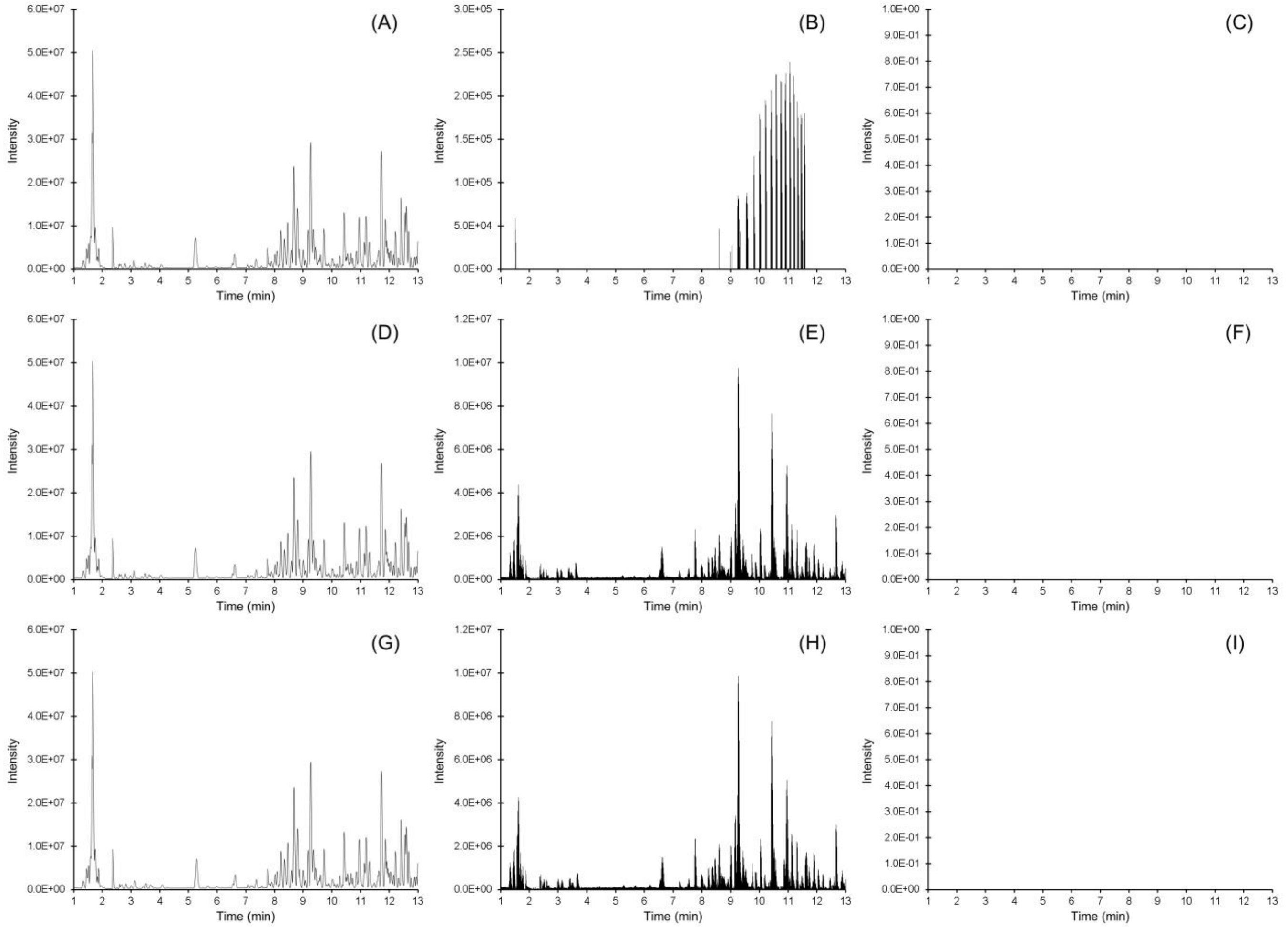

Figure S-35: Results of the application of different charge state filters to the HCD-CID screen with T-3RT in amaranth: (A) Dianion - MS chromatogram, (B) Dianion - HCD data points, (C) Dianion - CID data points, (D) Anion - MS chromatogram, (E) Anion - HCD data points, (F) Anion - CID data points, (G) Anion & Dianion - MS chromatogram, (H) Anion & Dianion - HCD data points, (I) Anion & Dianion - CID data points.

Figure S-36: Mass Trigger Controls, T-2RT

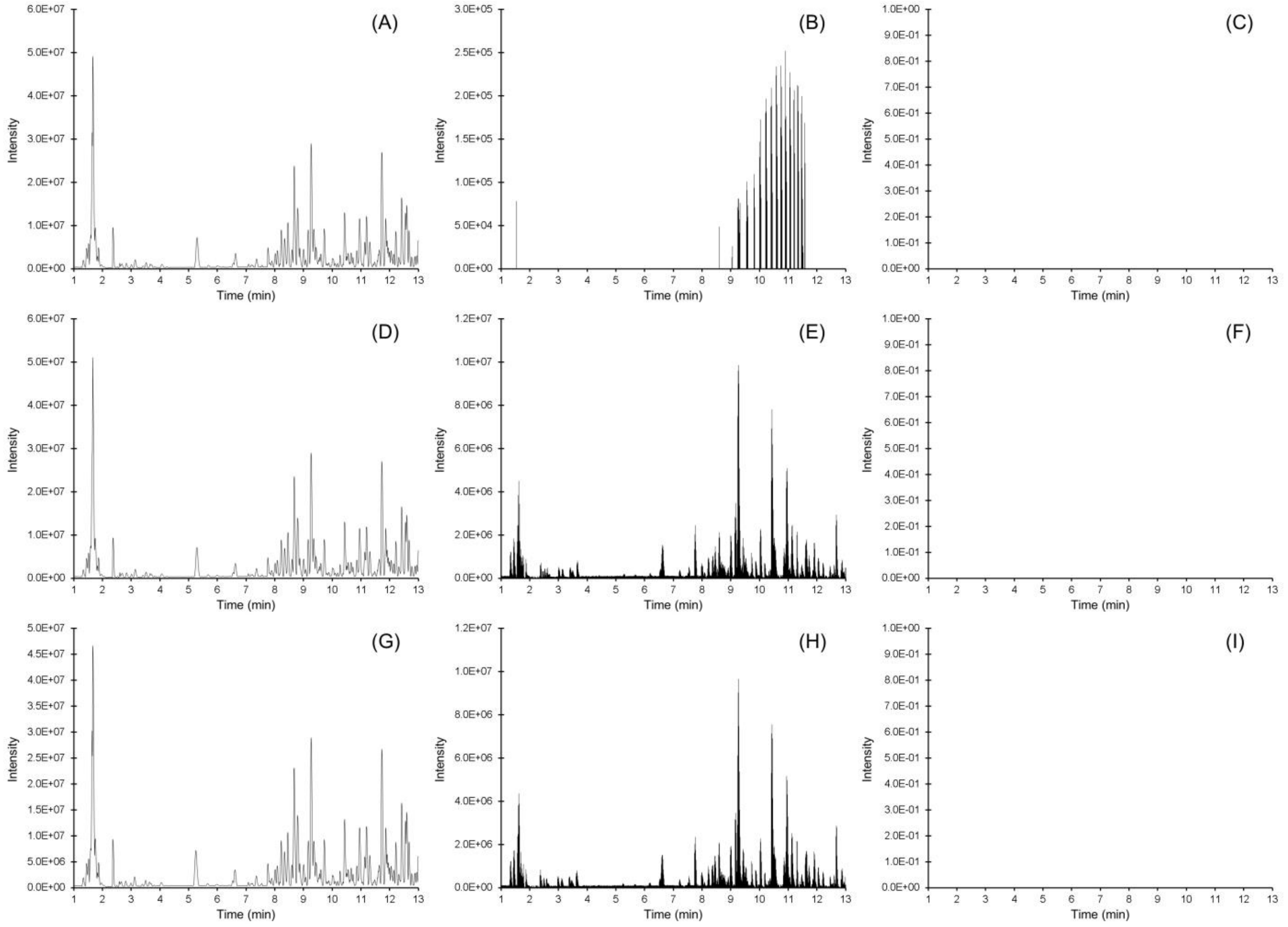

Figure S-36: Results of the application of different charge state filters to the HCD-CID screen with T-2RT in amaranth: (A) Dianion - MS chromatogram, (B) Dianion - HCD data points, (C) Dianion - CID data points, (D) Anion - MS chromatogram, (E) Anion - HCD data points, (F) Anion - CID data points, (G) Anion & Dianion - MS chromatogram, (H) Anion & Dianion - HCD data points, (I) Anion & Dianion - CID data points.

Figure S-37: Mass Trigger Controls, T-1RT

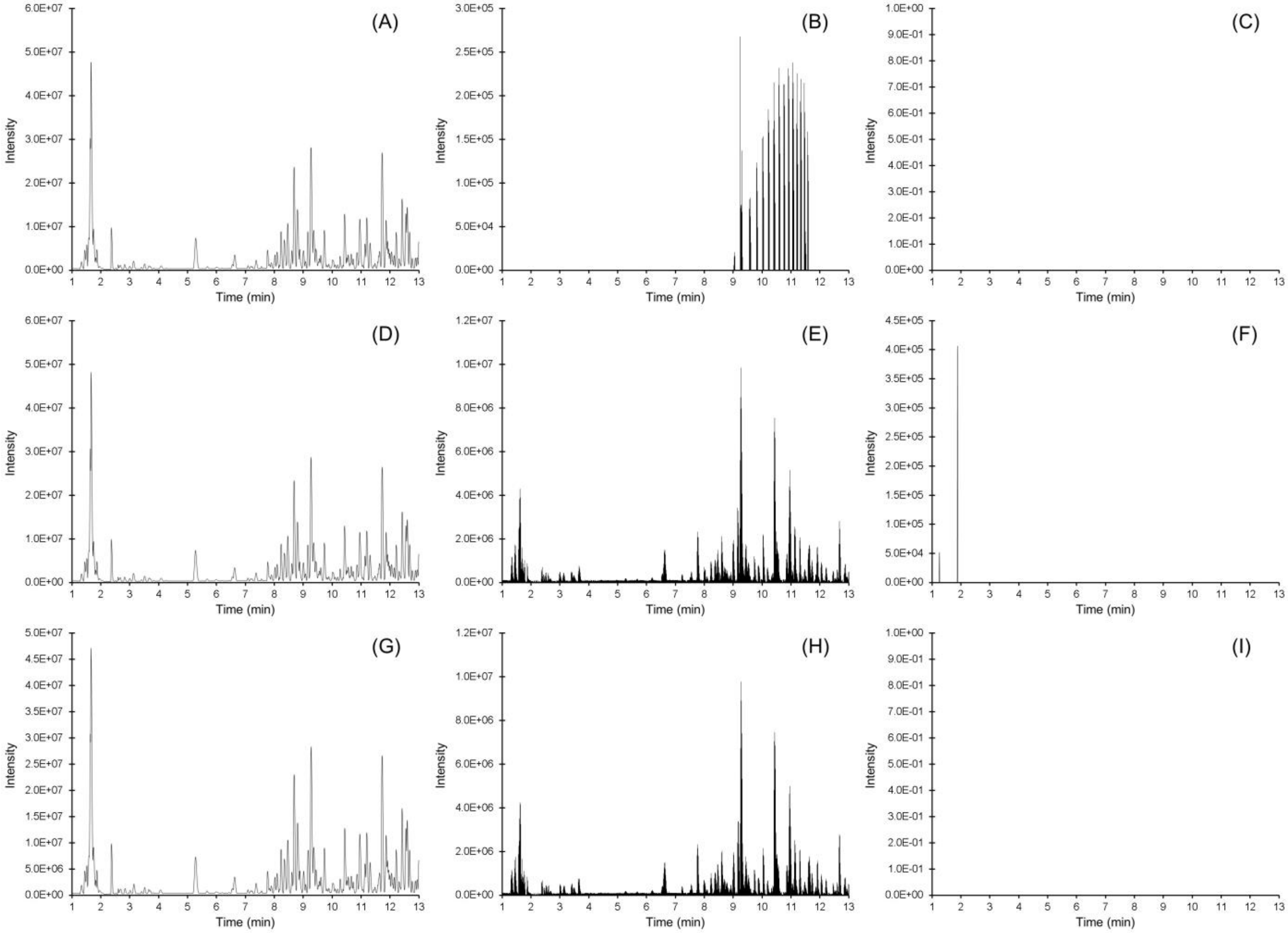

Figure S-37: Results of the application of different charge state filters to the HCD-CID screen with T-1RT in amaranth: (A) Dianion - MS chromatogram, (B) Dianion - HCD data points, (C) Dianion - CID data points, (D) Anion - MS chromatogram, (E) Anion - HCD data points, (F) Anion - CID data points, (G) Anion & Dianion - MS chromatogram, (H) Anion & Dianion - HCD data points, (I) Anion & Dianion - CID data points.

Figure S-38: HCD Product Ions - Potential Interferents

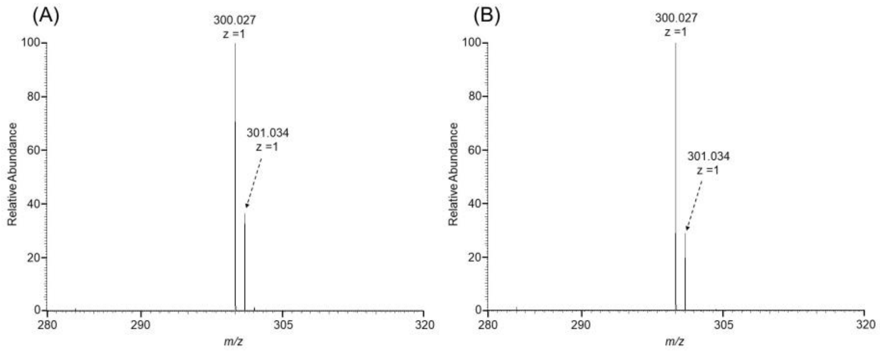

Figure S-38: Zoomed HCD fragmentation spectra for species present in amaranth that provide 300.027 and 301.034 product ions that could potentially interfere with the correct triggering of the ellagic acid 300.998 reporter ion without high resolution mass spectrometry: (A) rutin $[M-H]^-$, (B) isoquercitrin $[M-H]^-$.

Figure S-39: CID Data Points, 466 *m/z* – Screen Variants, T-3RT

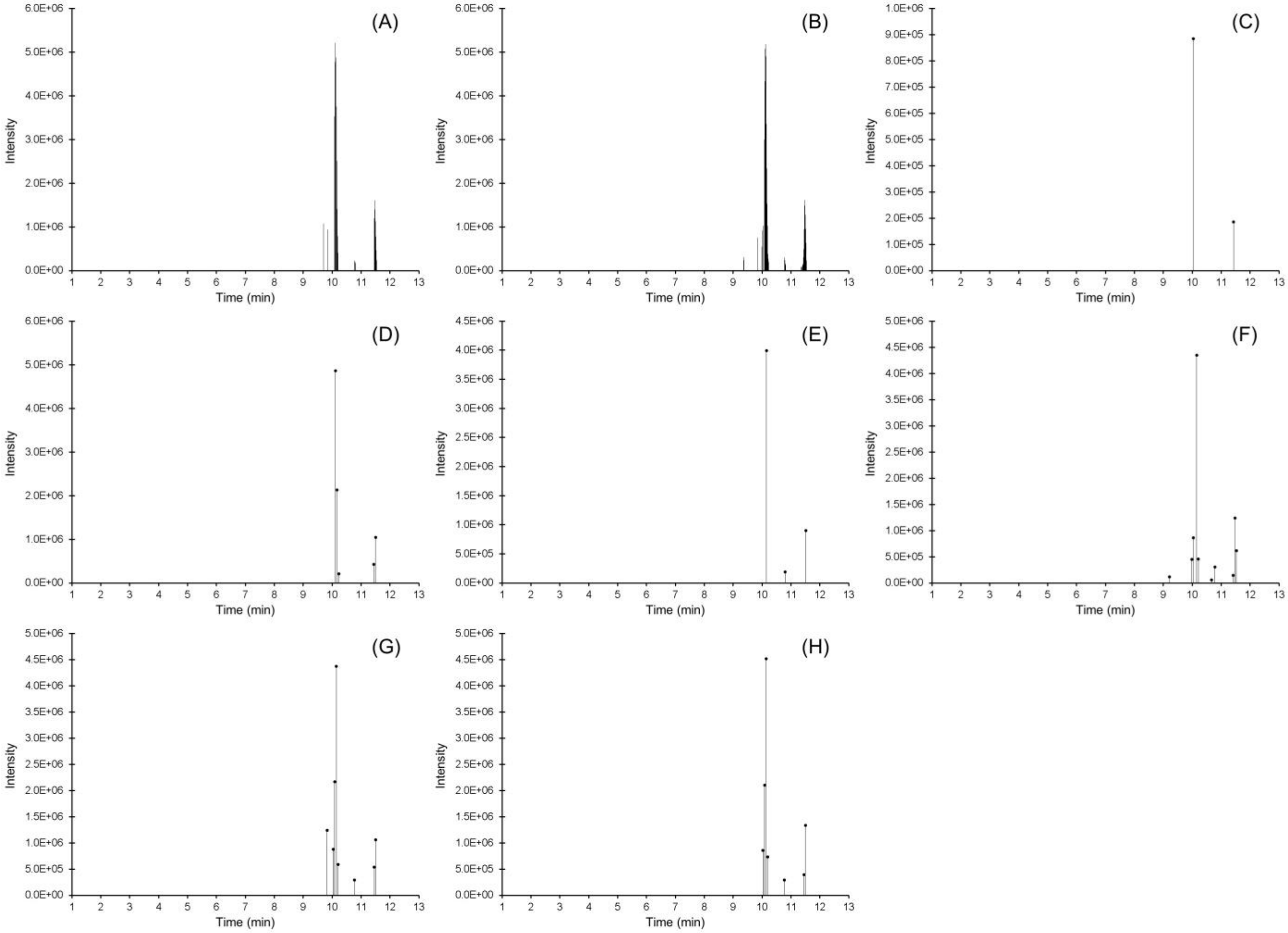

Figure S-39: Discrete CID data points for the 466 species present in strawberry observed under specific screen conditions as defined in Table 2 using T-3RT: (A) [I], (B) [III], (C) [IV], (D) [V], (E) [VI], (F) [VIII], (G) [IX], (H) [XI].

Figure S-40: CID Data Points, 466 *m/z* – Screen Variants, T-2RT

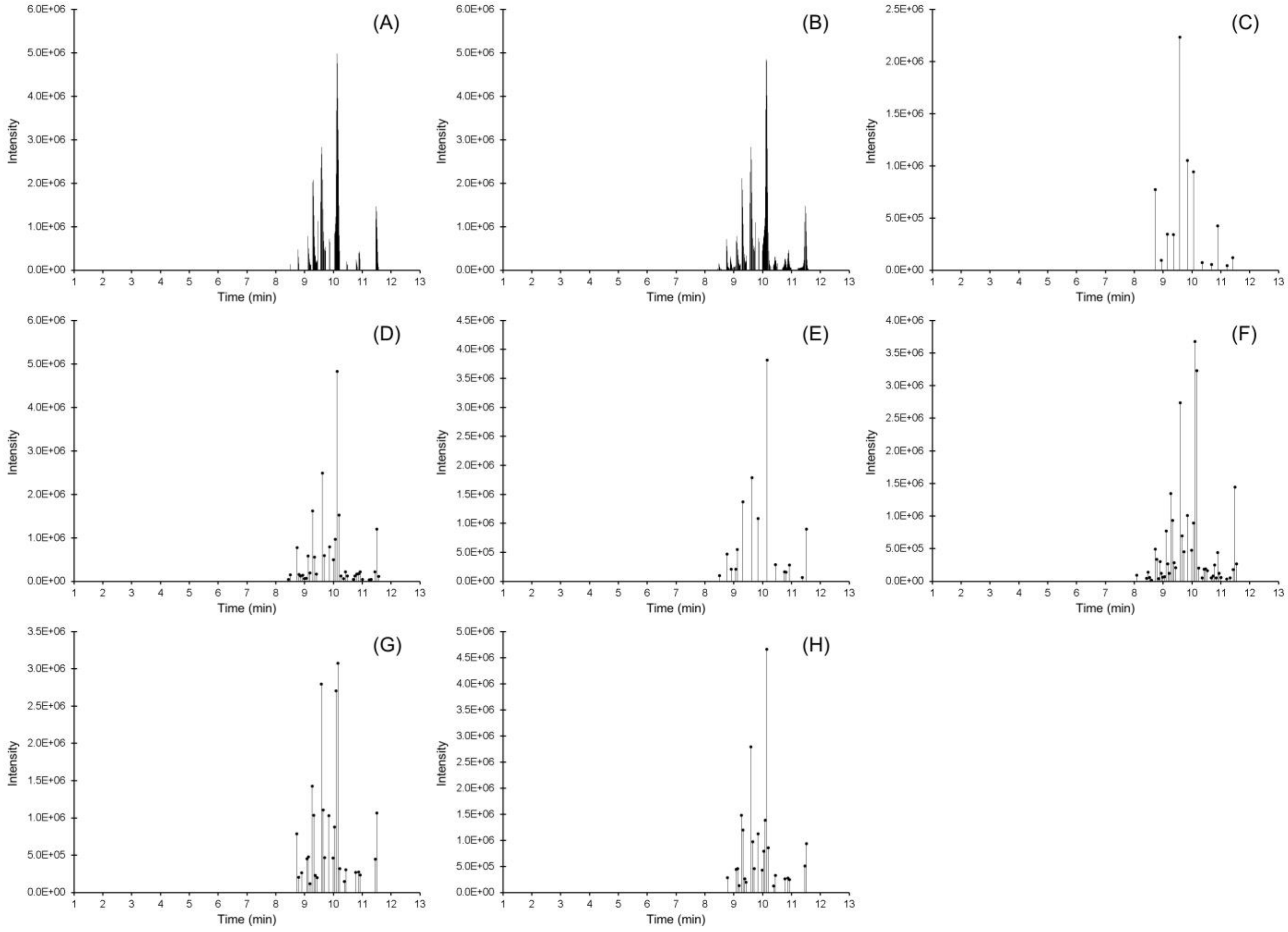

Figure S-40: Discrete CID data points for the 466 species present in strawberry observed under specific screen conditions as defined in Table 2 using T-2RT: (A) [I], (B) [III], (C) [IV], (D) [V], (E) [VI], (F) [VIII], (G) [IX], (H) [XI].

Figure S-41: Proposed Structures – Ions of Interest

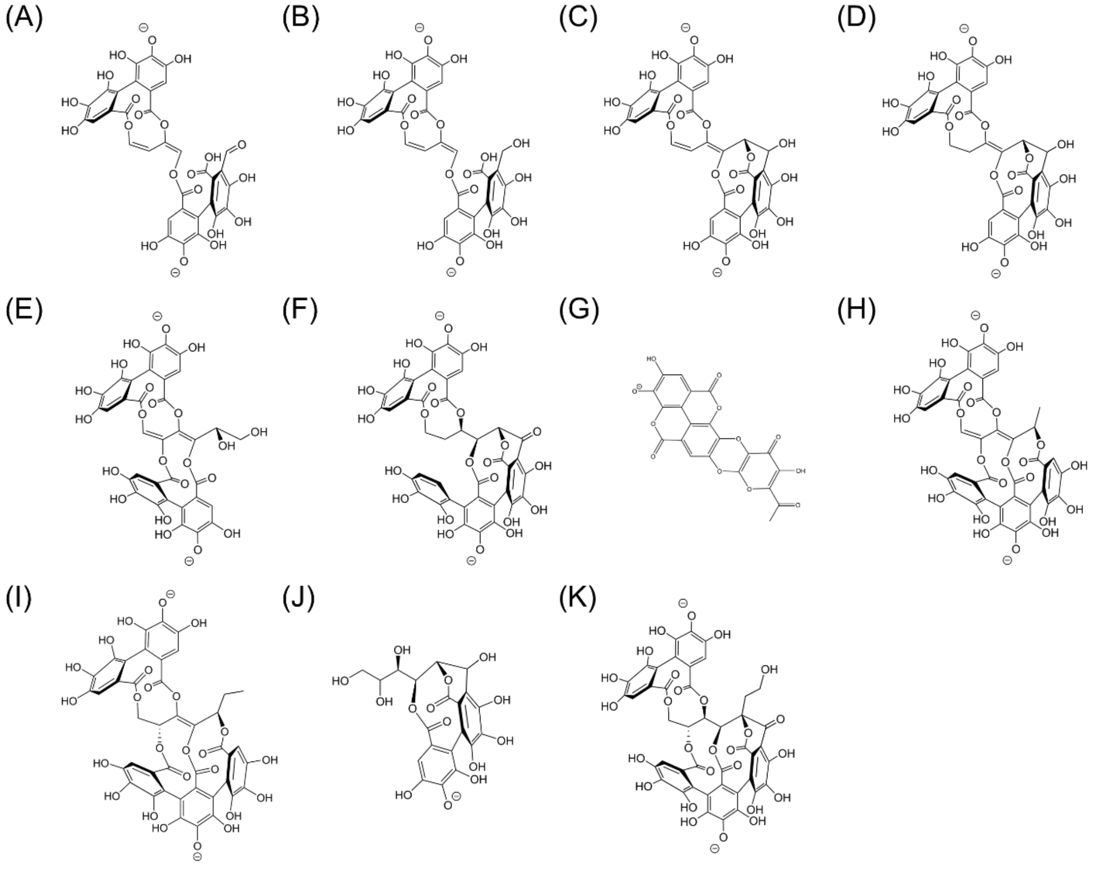

Figure S-41: Proposed even electron structures of significant ions of interest not used for classification of ellagitannins using the structure of standards as an initial point of reference with prioritization given to elemental compositions that were within +/-0.002 of observed values: (A) 375.017 obs., 375.018 calc., z = 2; (B) 376.024 obs., 376.025 calc., z = 2; (C) 381.017 obs., 381.018 calc., z = 2; (D) 382.025 obs., 381.025 calc., z = 2; (E) 390.022 obs., 390.023 calc., z = 2; (F) 444.033 obs., 444.033 calc., z = 2; (G) 450.993 obs., 450.994 calc., z = 1; (H) 457.023 obs., 457.023 calc., z = 2; (I) 465.038 obs., 465.039 calc., z = 2; (J) 481.061 obs., 481.062 calc., z = 1; (K) 487.035 obs., 487.034 calc., z = 2.

Supplementary Tables

Table S-1: Precursor CID, 457 Major Product Ion

MS/MS results from intact molecular ions with facile 457 formation.

Standard Compound	$[M-2H]^{2-}$ (*m/z*)	CID CE (%)	457.024 (rel.ab.)	300.999 (rel.ab.)	275.020 (rel.ab.)	249.040 (rel.ab.)
castalagin	466.029	25	36.9	100.0	49.4	21.4
vescalagin	466.029	25	100.0	6.7	10.0	5.5
acutissimin A	602.064	20	100.0	1.6	1.4	< 1.0
epiacutissimin A	602.064	15	100.0	1.2	< 1.0	< 1.0
Standard Compound	$[M-3H]^{3-}$ (*m/z*)	CID CE (%)	457.024 (rel.ab.)	300.999 (rel.ab.)	275.020 (rel.ab.)	249.040 (rel.ab.)
roburin A	615.704	20	100.0	5.8	2.3	2.0
roburin D	615.704	18	100.0	9.0	3.2	2.5
roburin B/C	659.718	16	57.5	1.1	1.3	< 1.0

Table S-2: Precursor CID, 487 Major Product Ion

MS/MS results from intact molecular ions with facile 487 formation.

Standard Compound	$[M-2H]^{2-}$ (*m/z*)	CID CE (%)	487.034 (rel.ab.)	465.038 (rel.ab.)	457.024 (rel.ab.)	300.999 (rel.ab.)	275.020 (rel.ab.)	249.040 (rel.ab.)
roburin E	532.050	20	100.0	6.5	4.8	7.2	4.2	18.4
grandinin	532.050	25	100.0	7.0	7.7	8.0	6.3	21.0

Table S-3: CID 457, MS^3

MS/MS results of 457 precursor isolated from MS/MS of intact molecular ion.

Standard Compound	300.999 (rel.ab.)	275.020 (rel.ab.)	249.040 (rel.ab.)
castalagin	79.4	100.0	49.8
vescalagin	60.2	100.0	45.2
acutissimin A	59.4	100.0	51.6
epiacutissimin A	59.5	100.0	50.1
roburin A	59.0	95.0	49.3
roburin D	54.8	86.8	44.3
roburin B/C	57.4	97.7	50.7

Table S-4: CID 487, MS^3

MS/MS results of 487 precursor isolated from MS/MS of intact molecular ion.

Standard Compound	465.038 (rel.ab.)	300.999 (rel.ab.)	275.020 (rel.ab.)	249.040 (rel.ab.)
roburin E	100.0	24.9	30.3	86.0
grandinin	100.0	31.9	37.9	99.8

Table S-5: CID 465, MS^4

MS/MS results of 465 precursor isolated from MS/MS of isolated 487.

Standard Compound	300.999 (rel.ab.)	275.020 (rel.ab.)	249.040 (rel.ab.)
roburin E	27.4	26.0	16.6
grandinin	24.6	23.5	14.1

Table S-6: ∑ (249, 275, 301) Intensities, Discrete HCD Series

Normalized sums of 249 / 275 / 301 intensities for discrete NCE values.

Standard Compound	0	5	10	15	20	25	30	35	40	50	60	70
castalagin	0.4	0.8	2.4	9.8	34.1	73.4	97.7	100	96.7	66.5	43.4	24.9
vescalagin	0.4	1	2.9	12.8	47.8	87.5	100	98.9	85	60.1	38.5	20.6
acutissimin A	0.1	0.3	1.1	4.8	17.6	41.2	75.5	95.8	100	87.6	67.6	44.4
epiacutissimin A	0.2	0.4	1.2	5.3	19.2	47.9	81.5	100	97.5	82.1	55.9	34.7
roburin A	0	0	0	3	24.1	57.4	84.4	100	96.9	87.7	59.7	41.5
roburin D	0	0	0.1	2.9	22.4	49.8	75.1	88.4	100	94.7	67.1	38.4
roburin B/C	0	0	0	2.1	17.4	45	70.9	88.6	100	87.6	61	35.8
grandinin	0.5	1.1	3.6	15.7	45.2	82.2	100	96.8	86.6	60.5	36.3	20.5
roburin E	0.4	1.2	6.1	30.2	71.9	100	95.6	85.8	68.9	40.8	20.7	9.4

Table S-7: ∑ (249, 275, 301) Intensities, Theoretical Stepped (+/-10) HCD Series

Theoretical normalized sums of 249 / 275 / 301 intensities for NCE values stepped by +/-10.

Standard Compound	10 +/- 10	20 +/-10	30 +/-10	40 +/-10	50 +/-10	60 +/-10
castalagin	14.1	51.4	87.6	100	79.2	51.7
vescalagin	20.8	61.5	95	100	74.9	48.6
acutissimin A	7.2	35.8	73.4	100	97	75.9
epiacutissimin A	7.9	39	75.9	100	90.2	66.2
roburin A	9	40.3	76.4	100	90.8	70.2
roburin D	8.3	36.2	73.2	100	97	74.2
roburin B/C	6.7	34.1	72.8	100	96.2	71.4
grandinin	19.9	60.2	93.8	100	74.2	47.5
roburin E	33.2	73.5	100	86.9	55.2	30
$\bar{x}$	14.12	48.00	83.12	98.54	83.86	59.52
σ	8.97	14.21	10.94	4.37	14.15	15.73

Table S-8: ∑ (249, 275, 301) Intensities, Stepped HCD Series

Normalized sums of 249 / 275 / 301 intensities for NCE values stepped by +/-10 and +/-5.

Standard Compound	25 +/- 10	30 +/-10	35 +/-10	40 +/-10	45 +/-10	25 +/- 5	30 +/-5	35 +/-5	40 +/-5	45 +/-5
castalagin	59.4	75.1	86.7	91	82.5	61.4	88.5	100	95.4	84.2
vescalagin	64.1	77.5	88.4	92.6	77.5	71.3	94.7	100	98.6	84.8
acutissimin A	46.3	64.2	77.3	89	91.1	45.7	72.7	100	96.2	97.5
epiacutissimin A	48.3	65.6	79.5	87.4	93.3	46.4	78.5	100	96.8	95.5
roburin A	53.1	71.6	89.7	81.9	90.4	50.5	81.2	100	99.3	95.2
roburin D	46.9	65.9	82.5	90.6	93.8	48.9	71.9	86	100	98.5
roburin B/C	57.8	76.1	92.3	94.3	85.5	62.7	89	100	99.9	90.6
grandinin	56.3	78.8	86.2	87.9	76.1	68.8	90	100	96.3	83.2
roburin E	57.9	70.8	81.9	82.1	83.1	63.1	79.9	94.2	100	77
$\bar{x}$	54.46	71.73	84.94	88.53	85.92	57.64	82.93	97.8	98.06	89.61
σ	6.2	5.51	4.96	4.29	6.62	9.85	8.02	4.82	1.87	7.59

Table S-9: Strawberry, Screen [I], T-3RT

Precursor ions present in strawberry leaves classified as ellagitannins under condition [I] using T-3RT.

No.	Scan No.	*m/z*	Z	Mass	RT (s)	Intensity
1	2560-4342	391.031	2	784.078	248-418	1.30E+06
2	3200-5536	453.039	2	908.094	308-530	7.48E+05
3	5321-7222	466.029	2	934.074	509-692	3.33E+06
4	6083-6651	467.036	2	936.089	583-637	3.62E+06
5	4590-4591	469.052	2	940.120	442-442	1.32E+05
6	3047-5434	475.034	2	952.084	294-520	6.14E+05
7	5300-5302	476.041	2	954.099	508-508	1.48E+05
8	1113-1525	481.064	1	482.072	110-149	4.53E+06
9	5208-6474	482.042	2	966.099	499-620	1.30E+06
10	2599-4289	491.029	2	984.073	252-414	3.15E+05
11	6961-7639	542.035	2	1086.086	667-733	1.47E+06
12	6561-7534	558.048	2	1118.111	628-723	4.84E+05
13	5319-5353	632.066	2	1266.148	509-513	8.74E+05
14	1753-5204	633.074	1	634.082	172-498	1.60E+06
15	1132-1140	637.067	1	638.075	112-113	7.00E+05
16	1603-2017	649.069	1	650.077	157-197	7.27E+05
17	4150-4772	663.084	1	664.092	401-459	3.59E+05
18	4746-4999	707.065	2	1416.146	457-479	1.73E+06
19	4849-4944	718.054	2	1438.125	466-474	1.01E+06
20	4754-4757	723.077	2	1448.170	458-458	1.73E+05
21	2295-4786	783.070	1	784.078	223-460	8.03E+05
22	5880-6060	783.070	2	1568.156	563-581	2.98E+06
23	4793-4795	785.085	1	786.093	461-461	2.37E+05
24	2552-2732	805.051	1	806.059	247-264	1.92E+05
25	2251-2426	815.095	1	816.103	220-235	3.23E+05
26	2813-4306	851.057	1	852.065	272-415	3.69E+05
27	4947-5827	858.068	2	1718.151	475-559	1.09E+06
28	6004-6031	874.080	2	1750.177	575-578	1.14E+06
29	6363-6600	934.074	2	1870.164	609-632	6.09E+06
30	6084-6150	935.080	1	936.088	583-589	1.09E+06
31	6371-6412	945.063	2	1892.141	610-614	1.40E+06
32	2981-5452	951.075	1	952.083	287-522	4.46E+05
33	5216-6478	965.091	1	966.099	499-620	6.11E+05

Table S-10: Strawberry, Screen [II], T-3RT

Precursor ions present in strawberry leaves classified as ellagitannins under condition [II] using T-3RT.

No.	Scan No.	*m/z*	Z	Mass	RT (s)	Intensity
1	1113-1518	481.064	1	482.072	110-149	4.65E+06
2	1131-1140	637.067	1	638.075	112-113	5.20E+05
3	1673-1988	649.068	1	650.076	164-195	7.68E+05
4	1734-5186	633.074	1	634.082	170-499	1.33E+06
5	2217-2414	815.095	1	816.103	218-236	2.97E+05
6	2258-4762	783.069	1	784.077	222-460	6.96E+05
7	2543-4267	805.051	1	806.059	248-413	1.95E+05
8	2837-4299	851.056	1	852.064	275-416	3.78E+05
9	2995-6119	951.075	1	952.083	290-588	4.13E+05
10	4136-4755	663.084	1	664.092	402-460	3.74E+05
11	4767-5419	785.085	1	786.093	461-522	2.71E+05
12	5185-6468	965.091	1	966.099	499-620	5.90E+05
13	5653-7212	933.065	1	934.073	544-692	2.00E+06
14	6053-6114	935.080	1	936.088	583-588	1.23E+06

Table S-11: Strawberry, Screen [III], T-3RT

Precursor ions present in strawberry leaves classified as ellagitannins under condition [III] using T-3RT.

No.	Scan No.	*m/z*	Z	Mass	RT (s)	Intensity
1	595-5386	391.031	2	784.077	165-681	5.92E+05
2	3086-3730	392.039	2	786.093	460-523	1.81E+05
3	988-1205	407.044	2	816.103	222-244	1.89E+05
4	2626-2663	425.025	2	852.065	413-416	1.42E+05
5	1757-4237	453.039	2	908.094	308-570	6.30E+05
6	3309-5529	466.029	2	934.073	481-694	1.54E+06
7	4364-4947	467.036	2	936.089	583-637	3.68E+06
8	2892-5372	469.052	2	940.120	440-680	3.23E+05
9	1551-4843	475.034	2	952.083	284-627	3.35E+05
10	3513-5074	476.041	2	954.098	503-649	1.43E+05
11	3460-4951	482.042	2	966.099	497-637	6.90E+05
12	1259-2675	491.028	2	984.073	250-417	2.30E+05
13	1007-3003	491.048	2	984.111	224-451	1.16E+05
14	4199-4242	498.055	2	998.126	567-570	2.87E+05
15	3855-4225	541.055	2	1084.126	535-569	2.07E+05
16	3349-5922	542.034	2	1086.085	485-755	8.40E+05
17	3426-3449	549.053	2	1100.122	494-496	1.18E+05
18	4842-5717	558.047	2	1118.111	627-723	3.71E+05
19	3413-3448	563.050	2	1128.117	492-496	1.19E+05
20	3574-3637	632.066	2	1266.148	509-515	6.67E+05
21	2551-3907	707.065	2	1416.145	405-540	1.00E+06
22	3050-3921	723.077	2	1448.170	456-541	1.61E+05
23	3456-4614	783.070	2	1568.155	497-606	9.82E+05
24	3467-3556	791.067	2	1584.149	498-507	1.29E+05
25	3234-5092	858.067	2	1718.151	474-651	5.52E+05
26	3779-4324	874.080	2	1750.176	528-579	7.99E+05
27	4163-4194	878.063	2	1758.141	563-566	4.37E+05
28	4520-4895	934.074	2	1870.163	597-632	6.09E+06
29	4482-4899	945.062	2	1892.140	594-632	9.73E+05

Table S-12: Strawberry, Screen [IV], T-3RT

Precursor ions present in strawberry leaves classified as ellagitannins under condition [IV] using T-3RT.

No.	Scan No.	*m/z*	Z	Mass	RT (s)	Intensity
1	3021-3762	392.039	2	786.093	508-588	1.51E+05
2	1795-3553	453.039	2	908.094	309-567	3.62E+05
3	2809-4671	466.029	2	934.074	481-685	1.27E+06
4	3798-4188	467.036	2	936.088	592-631	1.14E+06
5	1680-4127	475.034	2	952.084	285-625	2.78E+05
6	630-932	481.064	1	482.072	110-154	6.37E+06
7	2469-4181	482.042	2	966.099	435-631	4.52E+05
8	3368-3548	498.055	2	998.126	546-566	9.73E+04
9	3252-3589	541.055	2	1084.126	533-570	1.02E+05
10	648-828	549.050	1	550.058	112-142	1.52E+05
11	3612-4217	551.040	2	1104.096	572-634	6.26E+05
12	635-636	566.041	1	567.049	111-111	2.71E+05
13	4021-4927	594.076	2	1190.168	613-716	9.11E+04
14	3067-4014	616.090	2	1234.195	513-613	1.04E+05
15	1044-3827	633.073	1	634.081	170-594	2.16E+05
16	640-643	637.067	1	638.075	111-111	5.03E+05
17	646-1166	649.069	1	650.077	112-190	6.23E+05
18	2232-2618	663.084	1	664.092	398-455	1.14E+05
19	3840-3841	673.124	2	1348.263	596-596	6.55E+04
20	847-848	687.046	1	688.054	143-144	4.33E+05
21	2273-3214	707.064	2	1416.144	405-530	4.10E+05
22	1007-3973	783.070	1	784.078	164-609	5.30E+05
23	2922-3944	783.070	2	1568.156	494-606	7.49E+05
24	2657-3758	785.085	1	786.093	461-587	2.06E+05
25	2713-2945	791.067	2	1584.150	468-497	7.03E+04
26	2693-3640	799.064	1	800.072	465-575	9.14E+04
27	886-3168	801.080	1	802.088	147-524	9.59E+04
28	1479-2315	805.051	1	806.059	247-412	7.88E+04
29	1316-1459	815.095	1	816.103	219-244	1.50E+05
30	2750-4364	858.067	2	1718.150	473-651	5.65E+05
31	1616-2316	873.038	1	874.046	273-412	7.26E+04
32	3192-3633	874.080	2	1750.176	527-574	2.14E+05
33	4008-4532	917.164	1	918.172	612-669	8.38E+04
34	3854-4169	934.073	2	1870.162	597-630	3.00E+06
35	3708-3711	935.080	1	936.088	583-583	3.35E+05
36	3800-4240	937.088	1	938.096	592-637	8.92E+04

37	1685-4125	951.075	1	952.083	286-625	2.34E+05
38	647-855	963.133	1	964.141	112-144	2.43E+05
39	2950-4202	965.091	1	966.099	498-633	2.44E+05
40	1403-1405	983.103	1	984.111	234-234	5.32E+04
41	3624-4222	1018.076	2	2038.168	573-635	1.39E+05
42	3628-3631	1103.086	1	1104.094	574-574	1.89E+05
43	3995-3997	1149.163	2	2300.342	611-611	8.79E+04

Table S-13: Strawberry, Screen [V], T-3RT

Precursor ions present in strawberry leaves classified as ellagitannins under condition [V] using T-3RT.

No.	Scan No.	*m/z*	Z	Mass	RT (s)	Intensity
1	1323-5329	391.031	2	784.078	166-617	4.93E+05
2	3721-5018	392.039	2	786.093	462-588	1.66E+05
3	1761-1945	407.044	2	816.103	222-242	1.68E+05
4	2478-4807	453.039	2	908.094	309-568	5.54E+05
5	3919-6107	466.029	2	934.074	482-693	1.75E+06
6	4973-5514	467.036	2	936.088	583-635	2.56E+06
7	2301-5414	475.034	2	952.084	285-625	3.35E+05
8	4141-5657	476.041	2	954.099	504-649	1.49E+05
9	5374-5534	477.050	2	956.115	622-637	1.41E+05
10	809-1212	481.063	1	482.071	110-155	2.77E+06
11	3476-5524	482.042	2	966.099	436-636	5.31E+05
12	2027-3310	491.028	2	984.073	251-418	1.76E+05
13	4795-4838	498.055	2	998.126	567-571	1.51E+05
14	4539-4806	541.055	2	1084.126	543-568	1.64E+05
15	3946-6735	542.034	2	1086.085	485-756	7.05E+05
16	829-1122	549.050	1	550.058	112-145	2.39E+05
17	5419-6383	558.047	2	1118.111	626-721	1.99E+05
18	1328-1554	605.079	1	606.087	167-195	9.36E+04
19	1360-4105	633.074	1	634.082	171-500	8.98E+05
20	819-821	637.067	1	638.075	111-111	4.29E+05
21	825-1572	649.068	1	650.076	112-197	3.79E+05
22	3125-3691	663.084	1	664.092	398-459	2.82E+05
23	1104-1107	665.064	1	666.072	144-144	4.05E+05
24	5067-5070	675.121	2	1352.258	593-593	9.98E+04
25	1103-1106	687.046	1	688.054	144-144	4.36E+05
26	3196-4484	707.065	2	1416.145	405-537	7.81E+05
27	4422-4425	716.069	2	1434.154	531-532	9.70E+04
28	3668-4531	723.077	2	1448.170	456-542	1.27E+05
29	3940-5446	782.061	2	1566.139	484-629	1.70E+05
30	1310-5258	783.069	1	784.077	165-611	4.28E+05
31	4056-5161	783.070	2	1568.156	495-602	9.11E+05
32	3710-4363	785.084	1	786.092	461-526	1.51E+05
33	3757-4538	799.064	1	800.072	465-542	1.11E+05
34	1154-3990	801.080	1	802.088	148-489	1.00E+05
35	1993-3256	805.051	1	806.059	247-412	1.38E+05
36	1735-1963	815.095	1	816.103	219-244	2.36E+05

37	1753-2062	831.090	1	832.098	221-255	9.29E+04
38	2196-3308	851.057	1	852.065	272-417	3.56E+05
39	3838-5392	858.067	2	1718.151	473-623	5.34E+05
40	4745-4785	867.072	2	1736.161	562-566	7.52E+05
41	4398-4922	874.080	2	1750.176	529-578	4.71E+05
42	4974-5017	935.080	1	936.088	583-588	7.49E+05
43	5058-5502	937.087	1	938.095	592-634	1.18E+05
44	5076-5468	945.063	2	1892.141	594-631	5.93E+05
45	2311-5420	951.075	1	952.083	286-626	3.04E+05
46	824-1114	963.133	1	964.141	112-145	2.34E+05
47	4090-5344	965.091	1	966.099	499-619	2.24E+05
48	4876-4970	1103.087	1	1104.095	574-583	1.95E+05

Table S-14: Strawberry, Screen [VI], T-3RT

Precursor ions present in strawberry leaves classified as ellagitannins under condition [VI] using T-3RT.

No.	Scan No.	*m/z*	Z	Mass	RT (s)	Intensity
1	2431-3409	392.039	2	786.093	462-590	1.89E+05
2	1174-1274	407.044	2	816.103	226-244	1.99E+05
3	1623-3251	453.039	2	908.094	312-571	7.83E+05
4	2578-4192	466.029	2	934.074	484-690	2.85E+06
5	3374-3790	467.036	2	936.089	586-635	5.55E+06
6	2308-4117	469.052	2	940.120	443-678	3.23E+05
7	1511-3722	475.034	2	952.083	289-627	4.68E+05
8	543-721	481.064	1	482.072	112-144	1.61E+07
9	2268-3769	482.042	2	966.099	438-633	8.31E+05
10	1171-2316	491.048	2	984.112	226-444	9.81E+04
11	3231-3232	498.055	2	998.126	569-569	2.88E+05
12	2953-3227	541.055	2	1084.126	535-569	1.99E+05
13	546-713	549.051	1	550.059	112-144	2.44E+05
14	3711-4418	558.047	2	1118.111	626-724	3.08E+05
15	2769-2771	632.066	2	1266.148	512-512	6.11E+05
16	910-2685	633.074	1	634.082	175-499	2.12E+06
17	558-1032	649.068	1	650.076	113-196	5.92E+05
18	2058-2417	663.084	1	664.092	402-459	3.30E+05
19	2096-2986	707.065	2	1416.145	408-539	9.00E+05
20	2416-2478	718.054	2	1438.125	459-468	4.61E+05
21	2415-3017	723.077	2	1448.170	459-543	1.50E+05
22	870-2331	783.069	1	784.077	168-447	5.66E+05
23	2430-2858	785.084	1	786.092	462-524	2.55E+05
24	2482-3315	799.064	1	800.072	468-578	1.03E+05
25	992-2614	801.079	1	802.087	188-490	6.77E+04
26	1162-1231	815.095	1	816.103	225-236	4.31E+05
27	1442-2132	851.056	1	852.064	276-416	3.65E+05
28	2532-3707	858.067	2	1718.150	477-625	8.45E+05
29	3375-3379	935.080	1	936.088	586-587	1.66E+06
30	3797-3800	937.089	1	938.097	636-636	7.06E+04
31	1516-3723	951.075	1	952.083	290-627	4.20E+05
32	1188-3600	956.052	2	1914.119	229-612	2.44E+05
33	719-722	963.133	1	964.141	144-145	1.18E+05
34	2693-3772	965.091	1	966.099	501-633	3.26E+05

Table S-15: Strawberry, Screen [VII], T-3RT

Precursor ions present in strawberry leaves classified as ellagitannins under condition [VII] using T-3RT.

No.	Scan No.	*m/z*	Z	Mass	RT (s)	Intensity
1	807-1194	481.064	1	482.072	110-154	4.21E+06
2	832-1118	549.050	1	550.058	113-146	1.52E+05
3	812-813	566.041	1	567.049	111-111	2.79E+05
4	1356-3837	633.074	1	634.082	172-501	7.49E+05
5	818-820	637.067	1	638.075	112-112	5.02E+05
6	823-1567	649.068	1	650.076	112-199	4.10E+05
7	2977-3473	663.084	1	664.092	399-460	2.51E+05
8	1098-1099	687.046	1	688.054	144-144	4.40E+05
9	1306-4901	783.069	1	784.077	166-609	4.34E+05
10	3492-4683	785.085	1	786.093	462-588	1.92E+05
11	3528-4571	799.064	1	800.072	465-576	1.08E+05
12	1155-4078	801.080	1	802.088	149-526	9.05E+04
13	1706-1904	815.095	1	816.103	219-243	2.75E+05
14	2126-3138	851.056	1	852.064	272-419	3.11E+05
15	4933-5497	917.163	1	918.171	612-670	9.66E+04
16	4634-4669	935.080	1	936.088	583-587	9.60E+05
17	4718-5142	937.087	1	938.095	592-635	1.09E+05
18	2228-5058	951.074	1	952.082	287-626	3.50E+05
19	827-1106	963.133	1	964.141	112-144	2.51E+05
20	3820-4993	965.091	1	966.099	499-619	2.19E+05
21	4548-4972	987.072	1	988.080	573-617	1.15E+05
22	4552-4662	1103.086	1	1104.094	574-586	1.33E+05

Table S-16: Strawberry, Screen [VIII], T-3RT

Precursor ions present in strawberry leaves classified as ellagitannins under condition [VIII] using T-3RT.

No.	Scan No.	*m/z*	Z	Mass	RT (s)	Intensity
1	1857-2903	392.039	2	786.093	460-615	1.25E+05
2	1181-2528	453.039	2	908.094	310-570	5.28E+05
3	1987-3387	466.029	2	934.074	481-694	1.47E+06
4	2644-3064	467.036	2	936.089	583-637	2.87E+06
5	1076-2982	475.034	2	952.083	286-626	3.33E+05
6	2101-3137	476.041	2	954.098	504-649	1.49E+05
7	2948-3067	477.049	2	956.115	621-637	1.35E+05
8	1728-3073	482.041	2	966.099	436-638	5.55E+05
9	2160-2161	483.051	2	968.118	515-515	5.07E+04
10	800-1803	491.048	2	984.112	224-449	8.53E+04
11	2367-2523	498.055	2	998.126	546-570	1.92E+05
12	2294-2525	541.055	2	1084.126	534-570	1.54E+05
13	2052-2053	549.053	2	1100.122	494-494	6.86E+04
14	2964-3493	558.047	2	1118.110	623-721	1.91E+05
15	1580-2319	707.064	2	1416.145	406-539	9.63E+05
16	1843-2333	723.077	2	1448.171	457-541	8.20E+04
17	2055-2814	783.069	2	1568.155	495-605	6.72E+05
18	1937-3145	858.067	2	1718.150	473-651	4.46E+05
19	2763-3014	934.073	2	1870.161	597-630	3.78E+06
20	2730-3018	945.062	2	1892.140	593-630	4.81E+05

Table S-17: Strawberry, Screen [IX], T-3RT

Precursor ions present in strawberry leaves classified as ellagitannins under condition [IX] using T-3RT.

No.	Scan No.	*m/z*	Z	Mass	RT (s)	Intensity
1	1446-2533	453.039	2	908.094	310-530	7.44E+05
2	2498-3615	466.029	2	934.074	524-690	2.62E+06
3	2904-3291	467.036	2	936.089	583-635	3.11E+06
4	1434-2460	475.034	2	952.084	308-519	5.84E+05
5	512-715	481.063	1	482.071	110-146	3.64E+06
6	2355-3297	482.042	2	966.099	499-636	7.58E+05
7	2787-2788	498.055	2	998.126	567-567	2.59E+05
8	2691-2694	541.055	2	1084.126	554-554	2.80E+05
9	3244-3780	558.047	2	1118.111	628-722	4.13E+05
10	521-522	566.041	1	567.049	111-111	2.84E+05
11	2409-2429	632.066	2	1266.148	509-513	4.57E+05
12	1468-2353	633.074	1	634.082	314-499	1.54E+06
13	525-528	637.067	1	638.075	111-111	5.05E+05
14	534-963	649.068	1	650.076	112-197	5.37E+05
15	1804-2108	663.084	1	664.092	399-457	2.88E+05
16	700-703	687.046	1	688.054	144-144	4.48E+05
17	2067-2229	707.064	2	1416.145	450-477	1.50E+06
18	1181-2121	783.069	1	784.077	245-460	1.16E+06
19	2570-2896	783.070	2	1568.155	535-582	1.51E+06
20	2127-2483	785.085	1	786.093	461-522	2.63E+05
21	1062-1133	815.095	1	816.103	221-235	3.34E+05
22	1306-1892	851.056	1	852.064	274-416	3.48E+05
23	2209-2759	858.067	2	1718.150	474-562	7.63E+05
24	2906-2933	935.080	1	936.088	583-587	8.38E+05
25	2980-2981	937.087	1	938.095	593-593	3.18E+05
26	2989-3148	945.062	2	1892.141	594-613	8.07E+05
27	1439-2492	951.075	1	952.083	309-523	4.86E+05
28	527-704	963.133	1	964.141	111-144	2.93E+05
29	2361-3189	965.091	1	966.099	500-619	5.18E+05
30	3108-3202	1025.084	2	2052.183	608-621	3.28E+05

Table S-18: Strawberry, Screen [X], T-3RT

Precursor ions present in strawberry leaves classified as ellagitannins under condition [X] using T-3RT.

No.	Scan No.	*m/z*	Z	Mass	RT (s)	Intensity
1	514-714	481.063	1	482.071	110-145	4.10E+06
2	1553-2271	633.074	1	634.082	348-499	1.68E+06
3	523-526	637.067	1	638.075	111-111	3.50E+05
4	533-953	649.069	1	650.077	112-195	5.98E+05
5	1768-2049	663.084	1	664.092	400-457	2.85E+05
6	700-702	687.046	1	688.054	143-143	4.15E+05
7	1160-2066	783.069	1	784.077	244-461	1.16E+06
8	2386-2387	785.085	1	786.093	522-522	2.86E+05
9	1044-1110	815.095	1	816.103	220-234	3.41E+05
10	1287-1847	851.056	1	852.064	273-416	3.97E+05
11	2748-2767	935.080	1	936.088	583-587	9.22E+05
12	2802-2803	937.087	1	938.095	593-593	2.76E+05
13	1417-2392	951.075	1	952.083	309-522	4.59E+05
14	529-530	963.133	1	964.141	111-111	2.81E+05
15	2279-2983	965.091	1	966.099	500-619	4.52E+05

Table S-19: Strawberry, Screen [XI], T-3RT

Precursor ions present in strawberry leaves classified as ellagitannins under condition [XI] using T-3RT.

No.	Scan No.	*m/z*	Z	Mass	RT (s)	Intensity
1	1057-1880	453.039	2	908.094	310-529	7.41E+05
2	1853-2613	466.029	2	934.074	523-692	2.49E+06
3	2134-2388	467.036	2	936.088	583-634	3.13E+06
4	1048-1852	475.034	2	952.084	307-523	6.82E+05
5	1754-2392	482.041	2	966.099	498-635	7.87E+05
6	2048-2049	498.055	2	998.126	567-567	2.78E+05
7	1983-1984	541.055	2	1084.126	553-554	2.73E+05
8	2357-2713	558.047	2	1118.111	627-721	3.92E+05
9	1565-1673	707.065	2	1416.145	450-477	1.86E+06
10	1903-2112	783.069	2	1568.155	534-578	1.67E+06
11	1659-2026	858.067	2	1718.150	474-562	8.28E+05
12	2193-2290	945.062	2	1892.140	594-612	8.35E+05

Table S-20: Strawberry, Screen [I], T-2RT

Precursor ions present in strawberry leaves classified as ellagitannins under condition [I] using T-2RT.

No.	Scan No.	*m/z*	Z	Mass	RT (s)	Intensity
1	5109-5181	316.033	2	634.082	481-487	2.19E+06
2	2581-4420	391.031	2	784.077	249-419	6.71E+05
3	2317-2347	407.043	2	816.103	225-227	1.70E+05
4	3263-5648	453.039	2	908.093	312-531	4.88E+05
5	4743-4744	464.030	2	930.075	449-449	2.12E+05
6	5421-7381	466.029	2	934.073	510-693	1.98E+06
7	6079-6081	466.529	2	935.075	572-572	9.26E+05
8	6210-6813	467.036	2	936.088	584-638	3.09E+06
9	4674-4685	469.052	2	940.119	443-444	1.43E+05
10	3098-5544	475.034	2	952.083	296-521	4.08E+05
11	5414-5415	476.041	2	954.098	509-509	1.40E+05
12	6120-6503	477.019	2	956.054	576-610	1.09E+06
13	6859-7489	477.104	1	478.112	642-704	5.83E+05
14	6227-6275	478.027	2	958.070	586-590	9.19E+05
15	6022-7288	479.250	1	480.258	567-684	1.92E+06
16	1117-1538	481.063	1	482.071	110-150	4.31E+06
17	5317-6629	482.041	2	966.099	500-621	8.34E+05
18	3248-4724	486.024	2	974.065	310-447	2.54E+05
19	2638-4380	491.028	2	984.072	254-416	1.70E+05
20	6202-6216	529.044	2	1060.105	583-585	4.81E+05
21	7129-7802	542.034	2	1086.085	668-734	7.60E+05
22	1460-1461	549.050	1	550.058	143-143	5.19E+05
23	6193-6799	551.040	2	1104.095	583-637	1.35E+06
24	7086-8066	551.214	1	552.222	664-760	9.79E+05
25	7309-7330	553.025	2	1108.066	687-688	4.51E+05
26	6714-7692	558.047	2	1118.110	629-723	3.00E+05
27	6206-6218	562.030	2	1126.076	584-585	3.89E+05
28	5508-5547	598.078	2	1198.173	518-522	6.31E+05
29	6268-6334	617.032	2	1236.081	589-595	4.20E+06
30	6280-6300	628.022	2	1258.060	590-592	1.53E+06
31	4369-4734	631.058	2	1264.131	415-448	2.83E+05
32	4136-5727	631.058	1	632.066	394-539	1.75E+06
33	5435-5467	632.066	2	1266.147	511-514	5.64E+05
34	1780-5313	633.074	1	634.082	174-499	1.28E+06
35	1136-1137	637.067	1	638.075	112-112	5.22E+05
36	1616-2040	649.068	1	650.076	158-199	3.94E+05

37	4268-5162	655.055	1	656.063	406-486	5.29E+05
38	4223-4865	663.084	1	664.092	402-460	3.13E+05
39	7247-7298	692.030	2	1386.075	680-685	3.64E+05
40	4848-5092	707.064	2	1416.145	459-479	1.79E+06
41	4943-5045	718.054	2	1438.124	467-475	5.70E+05
42	4839-4858	723.077	2	1448.169	458-460	1.74E+05
43	5020-5029	729.045	2	1460.106	473-474	2.78E+05
44	2311-4881	783.069	1	784.077	224-462	7.81E+05
45	5986-6182	783.069	2	1568.154	564-582	1.86E+06
46	4888-4890	785.084	1	786.092	462-462	2.32E+05
47	3542-3640	789.050	2	1580.116	338-347	2.20E+05
48	3517-3846	798.055	2	1598.127	336-367	2.54E+05
49	2579-2624	805.050	1	806.058	249-253	1.48E+05
50	2274-2445	815.095	1	816.103	221-236	3.40E+05
51	2852-4378	851.056	1	852.064	274-416	2.64E+05
52	5050-5931	858.067	2	1718.150	476-559	8.67E+05
53	5973-6023	867.072	2	1736.160	563-567	1.31E+06
54	5775-5776	869.057	2	1740.130	544-544	3.97E+05
55	2889-2924	873.038	1	874.046	277-280	1.06E+05
56	6131-6154	874.079	2	1750.175	577-579	8.52E+05
57	6006-6017	878.062	2	1758.141	565-566	2.48E+05
58	5796-7356	933.064	1	934.072	546-691	1.24E+06
59	6506-6755	934.073	2	1870.162	610-632	4.40E+06
60	6223-6273	935.079	1	936.087	585-589	1.20E+06
61	6518-6561	945.061	2	1892.139	611-615	1.09E+06
62	7698-7741	949.059	1	950.067	724-728	3.20E+05
63	4967-5011	949.059	2	1900.135	469-472	2.72E+05
64	3028-5564	951.074	1	952.082	289-523	3.33E+05
65	5327-6631	965.090	1	966.098	500-621	6.01E+05
66	6644-7117	1009.069	2	2020.155	623-667	8.23E+05
67	6627-6638	1025.083	2	2052.181	621-622	2.32E+05

Table S-21: Strawberry, Screen [II], T-2RT

Precursor ions present in strawberry leaves classified as ellagitannins under condition [II] using T-2RT.

No.	Scan No.	*m/z*	Z	Mass	RT (s)	Intensity
1	6773-7403	477.104	1	478.112	642-704	6.54E+05
2	1124-1539	481.063	1	482.071	110-150	4.39E+06
3	1459-1462	549.050	1	550.058	142-143	1.76E+05
4	6990-7976	551.214	1	552.222	663-760	8.63E+05
5	6008-6038	611.256	1	612.264	571-574	1.43E+06
6	4118-5667	631.058	1	632.066	395-539	1.37E+06
7	1780-5267	633.074	1	634.082	173-500	1.01E+06
8	1143-1152	637.067	1	638.075	112-113	4.85E+05
9	1622-2056	649.068	1	650.076	158-199	3.81E+05
10	4248-5114	655.055	1	656.063	406-486	4.76E+05
11	4194-4836	663.084	1	664.092	402-461	2.92E+05
12	2027-2043	671.050	1	672.058	197-198	9.31E+04
13	2313-4843	783.069	1	784.077	225-462	7.41E+05
14	4848-5500	785.084	1	786.092	462-522	2.45E+05
15	2584-2953	805.050	1	806.058	249-283	1.31E+05
16	2270-2456	815.095	1	816.103	221-237	3.18E+05
17	2327-2339	831.090	1	832.098	226-227	1.12E+05
18	2852-4371	851.056	1	852.064	274-417	2.72E+05
19	2876-2936	873.038	1	874.046	276-281	9.70E+04
20	5729-7296	933.064	1	934.072	545-693	9.20E+05
21	6149-6217	935.079	1	936.087	584-589	1.02E+06
22	7602-7651	949.059	1	950.067	724-728	2.64E+05
23	3015-6190	951.074	1	952.082	289-587	3.40E+05
24	5266-6559	965.090	1	966.098	500-621	5.33E+05
25	7059-7060	1085.075	1	1086.083	669-669	2.59E+05
26	6150-6161	1103.086	1	1104.094	584-585	1.19E+05

Table S-22: Strawberry, Screen [III], T-2RT

Precursor ions present in strawberry leaves classified as ellagitannins under condition [III] using T-2RT.

No.	Scan No.	*m/z*	Z	Mass	RT (s)	Intensity
1	2721-3684	316.033	2	634.082	402-487	1.57E+06
2	588-5879	391.031	2	784.077	165-682	3.14E+05
3	3361-4087	392.038	2	786.093	460-524	1.51E+05
4	980-1212	407.044	2	816.103	221-243	1.25E+05
5	2861-2901	425.024	2	852.065	414-417	8.63E+04
6	1527-2893	436.015	2	874.046	275-417	6.36E+04
7	2907-2944	437.026	2	876.067	418-421	6.93E+04
8	6396-6414	452.049	2	906.115	744-746	2.66E+05
9	1857-4658	453.039	2	908.093	309-572	3.50E+05
10	3415-3427	462.044	2	926.105	464-466	6.94E+04
11	3185-4066	464.030	2	930.075	445-522	1.95E+05
12	5636-6306	465.020	2	932.057	660-729	3.06E+05
13	3614-6025	466.029	2	934.073	482-694	9.71E+05
14	4786-5389	467.036	2	936.088	585-637	3.17E+06
15	6417-6421	467.054	2	936.125	747-748	7.49E+04
16	5407-5487	468.044	2	938.104	639-646	1.46E+05
17	3146-5858	469.052	2	940.120	441-680	2.71E+05
18	3830-6299	474.026	2	950.068	501-729	2.97E+05
19	1617-5278	475.034	2	952.083	285-627	1.96E+05
20	6102-6104	475.052	2	952.119	706-707	6.62E+04
21	3845-5530	476.041	2	954.098	503-650	1.08E+05
22	4206-5999	477.019	2	956.054	534-692	3.99E+05
23	5402-5420	477.049	2	956.115	638-640	1.32E+05
24	4788-5392	478.027	2	958.070	585-637	5.83E+05
25	4961-5622	481.034	2	964.083	600-658	1.88E+05
26	3790-5408	482.041	2	966.099	498-639	4.35E+05
27	1698-4078	486.024	2	974.065	292-523	1.57E+05
28	1279-2918	491.028	2	984.072	250-419	1.30E+05
29	1001-3265	491.048	2	984.111	223-451	7.21E+04
30	5152-5204	493.033	2	988.081	617-621	1.86E+05
31	1850-3106	497.015	2	996.047	309-438	8.67E+04
32	4602-4645	498.055	2	998.126	568-571	2.90E+05
33	5020-5969	511.013	2	1024.042	605-689	1.10E+05
34	4805-5359	512.021	2	1026.057	587-635	2.59E+05
35	5572-5588	522.004	2	1046.025	654-655	1.17E+05
36	4766-5714	529.044	2	1060.105	583-667	2.27E+05

37	1883-3124	531.009	2	1064.034	312-439	1.03E+05
38	4237-4628	541.055	2	1084.126	536-570	1.89E+05
39	3666-6467	542.034	2	1086.084	486-755	4.30E+05
40	3751-3781	549.052	2	1100.121	494-497	7.37E+04
41	4678-5421	551.040	2	1104.095	574-640	6.66E+05
42	5914-6169	553.025	2	1108.066	685-714	2.31E+05
43	1191-1827	556.060	2	1114.136	241-307	6.91E+04
44	5279-6245	558.047	2	1118.110	627-724	2.09E+05
45	4767-4782	562.030	2	1126.077	583-585	2.88E+05
46	3733-3780	563.050	2	1128.116	493-497	7.08E+04
47	5737-6170	564.016	2	1130.048	669-714	1.17E+05
48	6222-6234	569.038	2	1140.092	722-723	7.50E+04
49	5522-6187	594.076	2	1190.168	649-716	5.45E+04
50	6158-6166	598.010	2	1198.035	713-714	7.47E+04
51	4004-4068	598.078	2	1198.173	517-522	5.59E+05
52	3980-4232	616.089	2	1234.195	515-536	1.09E+05
53	4827-4976	617.032	2	1236.081	588-601	2.10E+06
54	4838-4893	628.022	2	1258.060	589-594	1.10E+06
55	640-1873	629.042	2	1260.100	178-311	6.77E+05
56	2855-4151	631.058	2	1264.131	413-529	2.18E+05
57	3926-3993	632.066	2	1266.147	510-516	4.25E+05
58	3939-3975	643.056	2	1288.129	511-514	1.32E+05
59	5814-6331	692.029	2	1386.075	676-732	2.12E+05
60	4989-5011	701.035	2	1404.086	603-605	2.21E+05
61	2773-4285	707.064	2	1416.144	406-540	7.04E+05
62	4195-4199	716.069	2	1434.154	533-533	1.18E+05
63	3339-3578	718.054	2	1438.124	458-478	3.62E+05
64	3318-4330	723.077	2	1448.169	456-544	1.10E+05
65	3422-3546	729.044	2	1460.105	465-476	1.70E+05
66	3448-3525	740.036	2	1482.089	467-474	1.17E+05
67	3654-3698	782.061	2	1566.138	485-489	1.09E+05
68	3764-5030	783.069	2	1568.155	495-606	6.11E+05
69	877-2262	789.050	2	1580.116	210-352	1.65E+05
70	3796-3906	791.066	2	1584.148	498-508	7.12E+04
71	1533-4690	794.059	2	1590.135	276-575	3.18E+05
72	2086-2563	798.055	2	1598.127	333-387	1.80E+05
73	3530-5552	858.067	2	1718.150	474-652	3.60E+05
74	4751-4762	859.075	2	1720.165	581-583	1.84E+05
75	4546-4604	867.072	2	1736.160	563-568	9.60E+05
76	3678-4518	869.057	2	1740.130	487-560	1.76E+05
77	4158-4742	874.080	2	1750.176	530-580	5.54E+05
78	4562-4595	878.062	2	1758.141	564-567	3.06E+05
79	4324-4328	880.048	2	1762.112	544-544	1.15E+05

80	5025-5900	933.064	2	1868.144	606-683	6.42E+05
81	4934-5340	934.073	2	1870.162	598-633	2.74E+06
82	3740-3817	941.061	2	1884.139	493-500	8.54E+04
83	4898-5344	945.062	2	1892.140	595-633	5.56E+05
84	2479-3593	949.059	2	1900.133	380-480	1.52E+05
85	1041-5135	956.051	2	1914.117	226-615	2.06E+05
86	2828-2860	957.055	2	1916.126	411-414	8.55E+04
87	2491-2558	958.063	2	1918.143	381-387	7.11E+04
88	2954-3191	963.056	2	1928.129	422-445	1.46E+05
89	2952-3178	972.061	2	1946.138	422-444	1.55E+05
90	3432-3436	996.135	2	1994.286	466-466	6.70E+04
91	3437-3438	1007.125	2	2016.266	466-466	6.05E+04
92	5217-6251	1009.069	2	2020.155	622-724	4.03E+05
93	4700-4784	1018.075	2	2038.166	576-585	5.26E+05
94	5232-5698	1020.060	2	2042.137	623-665	1.71E+05
95	5062-5243	1025.083	2	2052.181	609-624	2.23E+05
96	5677-5680	1031.051	2	2064.117	664-664	7.04E+04
97	5103-5115	1138.172	2	2278.360	612-613	2.26E+05

Table S-23: Strawberry, Screen [IV], T-2RT

Precursor ions present in strawberry leaves classified as ellagitannins under condition [IV] using T-2RT.

No.	Scan No.	*m/z*	Z	Mass	RT (s)	Intensity
1	4072-4250	303.015	1	304.023	600-618	1.21E+05
2	1017-4860	391.031	2	784.077	164-681	2.74E+05
3	3133-3957	392.038	2	786.093	508-590	1.39E+05
4	1339-1415	407.043	2	816.103	221-233	5.42E+04
5	2380-2382	425.024	2	852.064	413-413	7.81E+04
6	1655-2385	436.015	2	874.046	276-414	5.05E+04
7	2414-2415	437.025	2	876.067	417-417	5.06E+04
8	5418-5420	452.049	2	906.115	745-745	3.39E+05
9	1823-3734	453.039	2	908.093	309-569	2.56E+05
10	3404-4367	453.104	1	454.112	536-629	1.45E+06
11	3140-3143	460.028	2	922.073	509-510	5.39E+04
12	2763-2764	462.044	2	926.105	463-464	5.82E+04
13	2602-3228	464.029	2	930.075	444-519	6.86E+04
14	2907-4890	466.029	2	934.073	481-685	7.28E+05
15	3769-3773	466.529	2	935.075	572-572	9.76E+05
16	3982-4401	467.035	2	936.087	592-633	9.59E+05
17	5436-5438	467.054	2	936.125	747-747	6.15E+04
18	4465-4469	468.044	2	938.103	638-639	1.46E+05
19	2580-4911	469.052	2	940.120	440-687	1.20E+05
20	2392-2393	470.009	2	942.034	414-415	5.82E+04
21	3109-5205	474.026	2	950.068	505-720	1.34E+05
22	1712-4335	475.033	2	952.083	286-626	1.47E+05
23	3098-4561	476.041	2	954.098	504-649	9.83E+04
24	3289-4912	477.019	2	956.055	524-687	1.43E+05
25	4904-4907	477.020	1	478.028	686-686	5.58E+04
26	4283-4455	477.050	2	956.115	621-638	1.10E+05
27	4492-5333	477.104	1	478.112	641-735	2.91E+05
28	4099-4102	477.521	1	478.529	603-603	1.01E+05
29	3916-4409	478.027	2	958.070	586-633	7.40E+05
30	3689-5036	479.250	1	480.258	565-701	2.55E+05
31	3681-4648	481.034	2	964.083	564-658	1.82E+05
32	628-929	481.064	1	482.072	110-153	6.53E+06
33	2541-4393	482.041	2	966.099	436-632	3.52E+05
34	3191-3192	483.051	2	968.118	514-515	5.41E+04
35	1734-3238	486.024	2	974.065	290-520	8.74E+04
36	4117-4120	490.039	1	491.047	604-605	1.32E+05

37	3654-4471	490.040	2	982.095	561-639	1.47E+05
38	1517-2428	491.028	2	984.071	251-419	5.88E+04
39	1350-2582	491.048	2	984.111	223-441	5.67E+04
40	2685-3371	491.177	1	492.185	453-532	1.64E+05
41	4125-4128	493.032	1	494.040	605-605	1.05E+05
42	3253-4267	493.033	2	988.081	521-619	1.27E+05
43	1820-3239	497.016	2	996.047	309-520	8.26E+04
44	3514-3714	498.055	2	998.126	547-567	8.13E+04
45	3281-3282	509.058	1	510.066	524-524	5.07E+04
46	3493-4925	511.013	2	1024.042	545-688	8.95E+04
47	3892-4421	512.021	2	1026.057	584-634	8.32E+04
48	3500-3819	522.004	2	1046.025	545-576	8.23E+04
49	4372-5460	523.203	1	524.211	630-750	1.31E+05
50	4253-4257	527.027	2	1056.070	618-618	8.63E+04
51	3812-4712	529.044	2	1060.105	576-664	1.17E+05
52	1832-3242	531.009	2	1064.034	310-520	7.33E+04
53	3888-3889	540.035	2	1082.086	583-584	6.80E+04
54	3392-3727	541.055	2	1084.126	535-568	8.33E+04
55	5531-5532	541.287	1	542.295	758-758	6.15E+04
56	2612-3247	542.001	2	1086.017	445-520	7.76E+04
57	2932-5485	542.034	2	1086.084	485-752	1.34E+05
58	3169-3172	548.063	2	1098.142	512-512	5.54E+04
59	645-827	549.050	1	550.058	112-141	1.17E+05
60	3021-3024	549.053	2	1100.122	494-494	8.25E+04
61	3778-4430	551.040	2	1104.095	573-635	4.24E+05
62	4893-5267	553.025	2	1108.066	685-727	1.45E+05
63	4200-4201	554.044	2	1110.104	613-613	1.14E+05
64	3504-4936	555.998	2	1114.012	546-689	7.07E+04
65	1469-1708	556.060	2	1114.136	242-286	6.33E+04
66	4298-5210	558.047	2	1118.110	623-721	9.48E+04
67	3799-4443	562.030	2	1126.077	575-636	1.15E+05
68	3006-3007	563.050	2	1128.117	493-493	5.55E+04
69	4741-5146	564.016	2	1130.047	667-714	8.10E+04
70	4354-5219	569.038	2	1140.092	628-722	9.03E+04
71	3878-3879	573.022	2	1148.059	582-582	5.71E+04
72	649-650	579.031	1	580.039	112-112	1.58E+05
73	4211-5167	594.076	2	1190.168	614-716	6.71E+04
74	3201-3204	598.079	2	1198.173	516-516	1.14E+05
75	1019-1188	605.078	1	606.086	164-193	5.56E+04
76	3894-3897	607.016	2	1216.047	584-584	5.13E+04
77	3181-3352	616.090	2	1234.196	513-531	5.40E+04
78	3867-4377	617.032	2	1236.080	581-630	6.76E+05
79	4340-4342	625.029	2	1252.073	627-627	5.88E+04

80	3940-3943	628.022	2	1258.060	588-588	9.23E+04
81	2379-3839	631.058	2	1264.131	413-578	2.11E+05
82	2246-3729	631.058	1	632.066	392-568	1.01E+06
83	3137-3341	632.065	2	1266.145	509-530	6.28E+04
84	1048-3040	633.073	1	634.081	169-497	1.29E+05
85	636-638	637.067	1	638.075	111-111	4.82E+05
86	3148-3151	643.057	2	1288.129	510-510	9.35E+04
87	2537-2539	647.056	2	1296.128	435-435	5.47E+04
88	644-1166	649.068	1	650.076	112-189	2.96E+05
89	2312-3994	655.055	1	656.063	403-593	9.59E+04
90	2285-2696	663.083	1	664.091	398-455	7.38E+04
91	851-855	665.064	1	666.072	144-144	3.31E+05
92	4425-4429	666.068	2	1334.152	635-635	7.77E+04
93	1018-1181	671.050	1	672.058	164-192	7.70E+04
94	4026-4028	673.123	2	1348.263	596-596	9.62E+04
95	3991-3995	675.121	2	1352.257	593-593	8.93E+04
96	832-834	681.058	1	682.066	142-142	1.02E+05
97	3993-3996	686.112	2	1374.240	593-593	7.83E+04
98	848-849	687.046	1	688.054	143-143	4.33E+05
99	4827-5248	692.030	2	1386.075	676-725	1.18E+05
100	4032-4318	701.035	2	1404.086	596-625	9.05E+04
101	2325-3359	707.064	2	1416.145	405-531	5.47E+05
102	4894-4896	708.043	2	1418.102	685-685	7.20E+04
103	3358-3360	716.068	2	1434.153	531-531	5.82E+04
104	2708-2811	718.054	2	1438.124	457-470	1.71E+05
105	2330-2908	723.043	1	724.051	405-481	1.34E+05
106	2702-3461	723.077	2	1448.170	456-542	6.72E+04
107	2770-2793	729.043	2	1460.102	464-467	1.26E+05
108	2783-2784	740.037	2	1482.090	466-466	6.44E+04
109	3921-3922	756.109	2	1514.233	586-587	5.81E+04
110	2925-4359	782.062	2	1566.139	484-629	1.02E+05
111	1006-4170	783.069	1	784.077	163-610	4.46E+05
112	3029-4090	783.069	2	1568.155	495-602	8.54E+05
113	2739-3254	785.084	1	786.092	461-521	1.89E+05
114	2804-3051	791.067	2	1584.149	469-498	6.04E+04
115	2387-3809	794.060	2	1590.135	414-576	3.44E+05
116	1942-2188	798.055	2	1598.126	333-382	1.59E+05
117	2775-3824	799.064	1	800.072	465-577	5.97E+04
118	882-3301	801.079	1	802.087	147-526	5.42E+04
119	1490-2374	805.050	1	806.058	246-412	6.48E+04
120	3792-3794	805.051	2	1612.117	574-574	1.10E+05
121	1320-1470	815.095	1	816.103	217-242	1.26E+05
122	1336-1502	831.090	1	832.098	220-248	5.40E+04

123	1347-1348	837.076	1	838.084	222-222	6.31E+04
124	1626-2359	851.056	1	852.064	271-411	7.00E+04
125	2842-4584	858.067	2	1718.150	473-652	1.81E+05
126	3843-3846	859.075	2	1720.166	579-579	1.31E+05
127	3663-3666	867.072	2	1736.159	562-562	2.68E+05
128	3682-3685	868.074	2	1738.165	564-564	7.93E+05
129	2857-3580	869.057	2	1740.130	474-554	6.62E+04
130	1637-2376	873.037	1	874.045	273-412	5.22E+04
131	3327-3810	874.080	2	1750.175	528-576	2.46E+05
132	3670-3671	878.063	2	1758.141	563-563	1.00E+05
133	3463-3592	880.049	2	1762.114	542-555	8.06E+04
134	3817-3821	885.071	2	1772.159	576-577	1.63E+05
135	3673-3675	889.053	2	1780.123	563-563	7.42E+04
136	3469-3471	891.039	2	1784.094	542-542	7.67E+04
137	3828-3829	896.062	2	1794.139	577-577	5.25E+04
138	4754-4756	917.163	1	918.171	669-669	6.96E+04
139	3534-4828	933.064	2	1868.144	549-676	4.19E+05
140	3099-4919	933.064	1	934.072	504-687	4.25E+05
141	4042-4375	934.071	2	1870.159	597-630	1.78E+06
142	3891-3895	935.079	1	936.087	584-584	2.27E+05
143	3985-4449	937.088	1	938.096	592-637	8.73E+04
144	3014-3015	941.062	2	1884.140	493-494	5.02E+04
145	3640-4349	942.069	2	1886.154	560-628	5.61E+04
146	3813-3815	944.055	2	1890.125	576-576	9.13E+04
147	4009-4387	945.062	2	1892.140	594-631	1.76E+05
148	4002-4004	945.564	2	1893.145	594-594	8.87E+04
149	2168-2889	949.059	2	1900.133	379-479	5.92E+04
150	4672-5203	949.060	1	950.068	660-720	8.10E+04
151	1710-4328	951.074	1	952.082	286-626	1.99E+05
152	3495-4933	955.046	1	956.054	545-689	1.02E+05
153	1361-4185	956.052	2	1914.119	224-612	2.97E+05
154	3057-3059	956.066	2	1914.147	499-499	5.60E+04
155	2362-2464	957.055	2	1916.126	411-425	7.94E+04
156	2173-2174	958.064	2	1918.143	380-380	5.05E+04
157	4994-5411	963.075	1	964.083	695-744	6.98E+04
158	640-857	963.133	1	964.141	111-144	2.40E+05
159	3056-4249	965.091	1	966.099	499-618	2.27E+05
160	4190-4225	967.040	2	1936.096	612-616	9.65E+04
161	1849-3248	973.056	1	974.064	313-520	6.12E+04
162	3586-4474	981.086	1	982.094	554-639	9.66E+04
163	3803-4238	987.072	1	988.080	575-617	9.97E+04
164	2780-2781	996.135	2	1994.285	465-465	5.66E+04
165	3905-3906	1003.067	1	1004.075	585-585	5.19E+04

166	2774-2777	1007.124	2	2016.265	465-465	5.25E+04
167	4286-5244	1009.070	2	2020.156	622-724	9.10E+04
168	3791-4437	1018.075	2	2038.167	574-636	9.05E+04
169	4291-4687	1020.061	2	2042.138	622-662	6.55E+04
170	4146-4951	1025.084	2	2052.184	607-690	1.29E+05
171	3808-3811	1029.066	2	2060.147	575-576	1.35E+05
172	4300-4713	1031.052	2	2064.119	623-664	5.91E+04
173	4734-4906	1085.075	1	1086.083	667-686	7.66E+04
174	3798-3801	1103.086	1	1104.094	575-575	1.87E+05
175	4187-4191	1138.172	2	2278.361	612-612	2.53E+05
176	4189-4192	1149.162	2	2300.340	612-612	1.48E+05
177	4169-4171	1158.104	2	2318.224	610-610	5.17E+04

Table S-24: Strawberry, Screen [V], T-2RT

Precursor ions present in strawberry leaves classified as ellagitannins under condition [V] using T-2RT.

No.	Scan No.	*m/z*	Z	Mass	RT (s)	Intensity
1	5391-5588	303.015	1	304.023	601-618	1.91E+05
2	3254-4120	316.033	2	634.082	401-486	1.28E+06
3	1300-5587	391.031	2	784.077	165-618	3.18E+05
4	3867-5259	392.039	2	786.093	461-590	1.50E+05
5	1752-1954	407.043	2	816.103	221-242	1.14E+05
6	3370-3413	425.024	2	852.065	412-416	6.04E+04
7	2252-3385	436.015	2	874.046	275-414	5.32E+04
8	3425-3428	437.026	2	876.067	417-418	5.57E+04
9	6923-6926	452.049	2	906.115	745-745	2.94E+05
10	2529-5036	453.039	2	908.093	309-570	3.10E+05
11	3898-3899	462.044	2	926.105	464-464	6.89E+04
12	3679-4474	464.029	2	930.075	444-519	1.18E+05
13	6077-6760	465.020	2	932.057	663-728	2.49E+05
14	4082-6404	466.029	2	934.073	481-693	1.06E+06
15	5219-5792	467.036	2	936.089	586-636	3.01E+06
16	5820-5892	468.044	2	938.104	639-646	1.41E+05
17	3649-6339	469.052	2	940.120	441-688	1.97E+05
18	3388-3389	470.009	2	942.034	414-414	5.73E+04
19	2325-5685	475.034	2	952.083	285-627	1.98E+05
20	4315-4356	476.041	2	954.098	504-508	1.16E+05
21	4534-6381	477.019	2	956.055	525-691	2.84E+05
22	5627-5821	477.050	2	956.116	622-639	1.07E+05
23	5448-5450	477.521	1	478.529	606-606	2.13E+05
24	5205-5764	478.027	2	958.070	585-634	5.20E+05
25	4994-6488	479.250	1	480.258	566-702	5.43E+05
26	5383-5937	481.034	2	964.083	601-650	2.40E+05
27	788-1149	481.063	1	482.071	110-150	3.40E+06
28	3602-5793	482.041	2	966.099	436-637	3.25E+05
29	2370-4487	486.024	2	974.065	290-521	1.48E+05
30	4952-5839	490.039	2	982.095	563-641	1.83E+05
31	2035-3407	491.028	2	984.072	251-416	1.15E+05
32	1772-3745	491.047	2	984.111	223-450	6.83E+04
33	3781-3784	491.177	1	492.185	453-453	1.79E+05
34	5432-5435	492.172	1	493.180	605-605	1.27E+05
35	5071-5611	493.033	2	988.081	573-620	1.32E+05
36	2514-4482	497.015	2	996.047	308-520	7.25E+04

37	5012-5015	498.055	2	998.126	568-568	2.67E+05
38	4768-6398	511.013	2	1024.042	546-693	1.32E+05
39	5207-5778	512.021	2	1026.057	585-635	1.38E+05
40	5590-5592	527.027	2	1056.070	618-618	8.05E+04
41	5178-6137	529.044	2	1060.105	583-669	1.51E+05
42	2537-4490	531.009	2	1064.034	310-521	8.50E+04
43	4856-5017	541.055	2	1084.126	554-569	1.10E+05
44	3702-3705	542.000	2	1086.016	446-446	6.36E+04
45	4117-7001	542.034	2	1086.084	485-752	3.69E+05
46	810-1108	549.050	1	550.058	112-145	1.49E+05
47	4214-4217	549.053	2	1100.121	495-495	9.25E+04
48	5077-5829	551.040	2	1104.095	574-640	3.50E+05
49	6315-6610	553.025	2	1108.066	685-714	2.00E+05
50	5527-5529	554.045	2	1110.105	613-613	1.20E+05
51	1965-2505	556.060	2	1114.136	243-307	6.66E+04
52	5657-6691	558.047	2	1118.110	624-722	1.31E+05
53	5179-5224	562.030	2	1126.077	583-587	1.59E+05
54	4201-4240	563.050	2	1128.116	494-498	7.61E+04
55	6136-6618	564.016	2	1130.047	668-715	9.85E+04
56	5905-6710	569.038	2	1140.093	647-724	6.39E+04
57	1065-3561	571.029	1	572.037	142-432	7.91E+04
58	5187-5190	573.021	2	1148.059	584-584	1.14E+05
59	4447-4497	598.079	2	1198.173	517-521	2.58E+05
60	1292-1528	605.078	1	606.086	164-193	6.31E+04
61	4421-4614	616.090	2	1234.195	514-532	9.82E+04
62	5193-5741	617.033	2	1236.081	584-632	1.25E+06
63	5251-5300	628.022	2	1258.061	589-593	3.31E+05
64	1419-2575	629.042	2	1260.100	179-314	6.37E+05
65	3375-4816	631.058	2	1264.131	413-550	2.23E+05
66	3166-5072	631.058	1	632.066	392-573	6.38E+05
67	1903-4528	631.224	1	632.232	237-524	3.99E+05
68	4380-4613	632.066	2	1266.147	511-532	2.02E+05
69	1334-4283	633.074	1	634.082	169-501	5.90E+05
70	802-804	637.067	1	638.075	111-111	4.91E+05
71	3678-3682	642.048	2	1286.112	443-444	6.42E+04
72	4387-4390	643.057	2	1288.129	511-512	9.34E+04
73	809-1547	649.068	1	650.076	112-195	2.29E+05
74	3280-5306	655.055	1	656.063	403-594	3.28E+05
75	1408-3844	663.084	1	664.092	177-459	1.47E+05
76	1086-1089	665.064	1	666.072	143-144	3.03E+05
77	1291-1544	671.050	1	672.058	164-195	7.70E+04
78	5307-5309	675.121	2	1352.257	594-594	2.15E+05
79	1074-1075	681.059	1	682.067	142-142	1.42E+05

80	5308-5310	686.111	2	1374.239	594-594	1.85E+05
81	1085-1088	687.046	1	688.054	143-144	3.54E+05
82	6230-6777	692.030	2	1386.075	677-730	1.78E+05
83	5398-5401	701.036	2	1404.087	602-602	1.03E+05
84	3301-4677	707.064	2	1416.144	405-538	6.30E+05
85	6316-6318	708.043	2	1418.102	685-686	6.70E+04
86	4635-4637	716.069	2	1434.155	534-534	7.69E+04
87	3819-4022	718.054	2	1438.124	457-476	2.86E+05
88	3303-4108	723.043	1	724.051	405-485	1.03E+05
89	3818-4727	723.077	2	1448.170	456-542	8.66E+04
90	3909-4030	729.044	2	1460.105	465-476	1.39E+05
91	3919-4023	740.037	2	1482.089	466-476	6.69E+04
92	5233-5234	756.108	2	1514.232	588-588	8.17E+04
93	4112-5720	782.061	2	1566.139	485-630	1.11E+05
94	1275-5514	783.069	1	784.077	163-612	3.61E+05
95	4251-5422	783.069	2	1568.155	498-604	5.04E+05
96	3861-4544	785.084	1	786.092	461-526	1.93E+05
97	1652-2832	789.050	2	1580.116	209-350	1.48E+05
98	3955-4339	791.067	2	1584.149	469-507	6.17E+04
99	3381-5127	794.060	2	1590.136	413-578	2.57E+05
100	2704-3087	798.055	2	1598.127	332-384	1.88E+05
101	3911-4697	799.064	1	800.072	465-540	1.01E+05
102	1127-4056	801.079	1	802.087	147-479	5.63E+04
103	1992-3421	805.050	1	806.058	246-417	8.82E+04
104	5087-5133	805.051	2	1612.118	575-579	2.40E+05
105	1721-1953	815.095	1	816.103	218-242	2.57E+05
106	1748-2043	831.090	1	832.098	221-252	7.13E+04
107	1767-1768	837.076	1	838.084	223-223	5.88E+04
108	2208-3435	851.056	1	852.064	270-418	2.00E+05
109	3997-5656	858.067	2	1718.150	473-624	3.56E+05
110	5149-5153	859.075	2	1720.165	580-580	2.06E+05
111	4960-5007	867.072	2	1736.160	563-568	5.16E+05
112	4015-4894	869.057	2	1740.131	475-558	1.23E+05
113	2221-3414	873.038	1	874.046	272-417	7.23E+04
114	4587-5144	874.080	2	1750.176	530-580	2.63E+05
115	4967-4971	878.062	2	1758.141	564-564	2.62E+05
116	4733-4888	880.048	2	1762.112	543-557	1.03E+05
117	5116-5119	885.071	2	1772.159	577-577	1.78E+05
118	4974-4978	889.054	2	1780.123	565-565	1.24E+05
119	5160-6221	933.064	2	1868.145	581-677	4.43E+05
120	4321-6390	933.064	1	934.072	505-692	5.01E+05
121	5348-5725	934.073	2	1870.161	598-631	2.24E+06
122	5197-5248	935.079	1	936.087	584-589	5.49E+05

123	5298-5786	937.087	1	938.095	593-636	1.43E+05
124	4207-4248	941.061	2	1884.139	494-498	7.93E+04
125	5736-5740	942.070	2	1886.156	631-632	3.59E+04
126	5314-5734	945.062	2	1892.140	595-631	3.62E+05
127	3045-4054	949.059	2	1900.134	379-479	1.29E+05
128	6057-6754	949.059	1	950.067	661-728	1.75E+05
129	2334-5277	951.074	1	952.082	286-591	2.21E+05
130	4769-5463	955.047	1	956.055	546-607	1.29E+05
131	1799-5552	956.051	2	1914.118	226-615	1.71E+05
132	3336-3518	957.055	2	1916.127	409-428	6.01E+04
133	805-806	963.133	1	964.141	111-112	2.91E+05
134	1791-2072	965.053	2	1932.122	225-255	1.29E+05
135	4259-5604	965.091	1	966.099	499-620	1.89E+05
136	5536-5538	967.042	2	1936.100	614-614	1.13E+05
137	3467-3674	972.061	2	1946.138	422-443	1.17E+05
138	2557-3249	973.056	1	974.064	312-400	6.02E+04
139	4880-5840	981.086	1	982.094	556-641	1.49E+05
140	5581-5584	987.072	1	988.080	618-618	1.01E+05
141	3920-3922	996.136	2	1994.287	466-466	5.60E+04
142	3910-3913	1007.125	2	2016.266	465-466	6.10E+04
143	5630-6382	1009.070	2	2020.156	622-691	2.57E+05
144	5085-5585	1018.075	2	2038.166	575-618	2.18E+05
145	5637-6112	1020.061	2	2042.138	623-666	9.25E+04
146	5464-5666	1025.084	2	2052.184	608-625	1.19E+05
147	5111-5112	1029.065	2	2060.146	577-577	1.42E+05
148	5652-6092	1031.053	2	2064.121	624-664	8.32E+04
149	6129-6332	1085.075	1	1086.083	668-687	8.72E+04
150	5101-5192	1103.086	1	1104.094	576-584	1.28E+05
151	5518-5521	1138.172	2	2278.359	612-612	2.78E+05
152	5520-5522	1149.163	2	2300.342	612-612	1.34E+05
153	5502-5505	1158.105	2	2318.225	611-611	1.83E+05

Table S-25: Strawberry, Screen [VI], T-2RT

Precursor ions present in strawberry leaves classified as ellagitannins under condition [VI] using T-2RT.

No.	Scan No.	*m/z*	Z	Mass	RT (s)	Intensity
1	3705-3707	303.015	1	304.023	602-602	1.38E+05
2	2176-2710	316.033	2	634.082	404-483	2.43E+06
3	1227-4366	391.031	2	784.077	224-683	3.52E+05
4	3009-3594	392.039	2	786.093	523-590	1.66E+05
5	1226-1326	407.043	2	816.103	224-242	1.37E+05
6	2252-2255	425.024	2	852.065	416-417	8.28E+04
7	2253-2256	436.015	2	874.047	417-417	5.51E+04
8	2280-2282	437.025	2	876.067	420-420	6.46E+04
9	4811-4812	452.049	2	906.115	746-746	1.75E+05
10	1704-3432	453.039	2	908.094	313-572	5.35E+05
11	3136-3959	453.104	1	454.112	537-631	1.47E+06
12	2581-2582	462.044	2	926.105	464-464	5.95E+04
13	2460-2464	464.030	2	930.075	447-447	2.43E+05
14	2718-4421	466.029	2	934.074	484-690	1.67E+06
15	3561-3998	467.036	2	936.089	587-635	3.96E+06
16	2444-2445	469.052	1	470.060	444-445	6.11E+04
17	2434-4338	469.052	2	940.120	443-679	2.66E+05
18	1582-3932	475.034	2	952.083	289-627	2.90E+05
19	2857-2859	476.041	2	954.098	504-504	1.07E+05
20	3228-4423	477.019	2	956.054	548-691	5.97E+05
21	3879-4033	477.050	2	956.115	621-639	9.88E+04
22	3737-3739	477.521	1	478.529	605-606	2.06E+05
23	3563-4005	478.027	2	958.070	587-636	6.04E+05
24	3401-4498	479.250	1	480.258	569-701	9.99E+05
25	3688-4103	481.034	2	964.084	600-648	2.74E+05
26	601-772	481.064	1	482.072	113-144	1.29E+07
27	2385-4025	482.041	2	966.099	437-638	5.70E+05
28	1703-2989	486.024	2	974.065	313-521	2.34E+05
29	3362-4027	490.039	2	982.095	563-638	2.90E+05
30	1396-2248	491.028	2	984.073	255-416	1.56E+05
31	1220-2438	491.048	2	984.111	223-444	6.60E+04
32	3728-3730	492.172	1	493.180	604-605	9.81E+04
33	3088-3862	493.033	2	988.081	532-619	1.72E+05
34	1698-2991	497.015	2	996.047	313-522	8.45E+04
35	3405-3408	498.055	2	998.126	569-570	3.07E+05
36	3219-4424	511.013	2	1024.042	547-691	1.47E+05

37	4372-4373	511.165	1	512.173	683-683	6.69E+04
38	3564-4012	512.021	2	1026.058	587-637	1.73E+05
39	3467-4146	522.004	2	1046.025	576-654	1.10E+05
40	3867-3868	527.027	2	1056.070	619-619	5.92E+04
41	3537-4250	529.044	2	1060.105	584-666	2.32E+05
42	1695-2997	531.009	2	1064.034	312-522	8.76E+04
43	3117-3399	541.055	2	1084.126	535-569	1.46E+05
44	2471-2474	542.000	2	1086.017	448-448	6.01E+04
45	2735-4866	542.034	2	1086.085	487-754	6.35E+05
46	4093-4095	543.042	2	1088.100	647-647	5.23E+04
47	611-765	549.050	1	550.058	114-143	1.29E+05
48	2800-2803	549.052	2	1100.121	496-496	7.78E+04
49	3470-4020	551.040	2	1104.095	576-637	9.14E+05
50	4404-4407	553.025	2	1108.066	688-688	3.20E+05
51	3819-3822	554.045	2	1110.105	614-614	6.12E+04
52	3225-3226	555.998	2	1114.012	547-547	5.70E+04
53	1593-1594	556.060	2	1114.135	291-292	8.17E+04
54	3912-4647	558.047	2	1118.110	625-723	1.95E+05
55	3472-3542	562.030	2	1126.076	576-584	2.24E+05
56	2795-2798	563.050	2	1128.116	495-496	8.79E+04
57	4276-4400	564.016	2	1130.048	670-688	1.01E+05
58	583-586	566.041	1	567.049	111-112	1.41E+05
59	3539-3543	573.021	2	1148.059	584-585	1.28E+05
60	588-592	579.031	1	580.039	112-112	1.36E+05
61	3834-3835	594.076	2	1190.167	615-615	5.84E+04
62	2965-2969	598.078	2	1198.173	519-519	6.18E+05
63	921-1071	605.078	1	606.086	167-193	8.01E+04
64	4157-4259	611.068	2	1224.152	655-668	9.00E+04
65	2945-3097	616.089	2	1234.195	516-533	9.47E+04
66	3514-3972	617.033	2	1236.081	581-632	1.56E+06
67	2323-3256	631.058	2	1264.131	427-551	3.29E+05
68	2123-3422	631.058	1	632.066	396-571	1.53E+06
69	2919-3083	632.066	2	1266.147	512-532	4.49E+05
70	950-2824	633.074	1	634.082	173-499	1.23E+06
71	606-607	637.067	1	638.075	113-114	1.90E+05
72	2920-2922	643.057	2	1288.130	512-513	1.11E+05
73	609-1074	649.068	1	650.076	114-194	3.39E+05
74	2206-3637	655.055	1	656.063	409-595	4.58E+05
75	2164-2543	663.084	1	664.092	403-458	3.43E+05
76	4364-4678	692.030	2	1386.076	682-727	2.59E+05
77	3663-3926	701.035	2	1404.086	597-627	1.05E+05
78	2202-3155	707.064	2	1416.145	408-539	6.21E+05
79	4392-4393	708.043	2	1418.101	687-687	8.31E+04

80	3096-3098	716.069	2	1434.154	533-533	7.03E+04
81	2538-2607	718.054	2	1438.124	458-467	3.32E+05
82	2207-2714	723.043	1	724.051	409-484	1.85E+05
83	2545-3185	723.077	2	1448.169	459-543	1.11E+05
84	2609-2611	729.044	2	1460.105	467-468	1.57E+05
85	2610-2650	740.037	2	1482.090	467-474	8.36E+04
86	2726-3957	782.061	2	1566.138	486-631	9.67E+04
87	910-2557	783.069	1	784.077	165-461	5.22E+05
88	2810-3464	783.069	2	1568.155	497-575	7.01E+05
89	2561-3011	785.085	1	786.093	461-523	2.54E+05
90	1157-1865	789.050	2	1580.116	211-345	1.62E+05
91	2626-2887	791.067	2	1584.149	470-508	6.12E+04
92	2257-3465	794.060	2	1590.135	417-575	2.85E+05
93	1837-2050	798.056	2	1598.127	341-382	2.65E+05
94	2614-3166	799.064	1	800.072	468-540	8.91E+04
95	1032-3036	801.079	1	802.087	186-526	5.56E+04
96	1372-1476	805.050	1	806.058	250-269	1.05E+05
97	3462-3466	805.051	2	1612.118	575-576	1.73E+05
98	1214-1282	815.095	1	816.103	222-234	4.08E+05
99	3624-3625	825.117	2	1652.250	593-594	5.56E+04
100	1217-1393	831.090	1	832.098	223-254	7.15E+04
101	1222-1223	837.076	1	838.084	223-224	6.18E+04
102	1515-2246	851.056	1	852.064	277-416	3.26E+05
103	2666-3913	858.067	2	1718.150	476-626	4.82E+05
104	3506-3507	859.075	2	1720.165	580-580	1.82E+05
105	3374-3377	867.072	2	1736.160	565-565	1.18E+06
106	2667-3303	869.057	2	1740.130	476-557	1.43E+05
107	1516-2254	873.038	1	874.046	277-417	9.55E+04
108	3070-3492	874.080	2	1750.176	530-578	4.00E+05
109	3375-3378	878.062	2	1758.140	565-565	2.13E+05
110	3197-3307	880.048	2	1762.112	544-557	7.48E+04
111	3493-3495	885.072	2	1772.160	578-578	1.22E+05
112	4280-4281	917.164	1	918.172	670-671	5.87E+04
113	2871-4422	933.064	1	934.072	506-690	7.73E+05
114	4042-4325	933.064	2	1868.145	640-677	1.54E+05
115	3655-3978	934.073	2	1870.163	597-633	3.07E+06
116	3562-3566	935.080	1	936.088	587-587	1.40E+06
117	3991-4016	937.088	1	938.096	635-637	8.79E+04
118	2794-2797	941.061	2	1884.138	495-495	1.00E+05
119	3648-3980	945.062	2	1892.140	596-633	5.10E+05
120	2058-2621	949.059	2	1900.133	384-469	1.98E+05
121	4196-4675	949.060	1	950.068	659-727	2.42E+05
122	1589-3933	951.074	1	952.082	291-628	2.66E+05

123	3220-4425	955.046	1	956.054	547-691	1.20E+05
124	1237-3805	956.051	2	1914.119	226-612	1.71E+05
125	2231-2232	957.056	2	1916.128	414-414	6.07E+04
126	2059-2061	958.064	2	1918.143	384-384	7.23E+04
127	596-599	963.133	1	964.141	113-113	1.16E+05
128	2832-3982	965.090	1	966.098	500-633	2.28E+05
129	3802-3813	967.041	2	1936.098	612-613	9.37E+04
130	2313-2402	972.061	2	1946.138	425-439	1.42E+05
131	1712-2999	973.056	1	974.064	315-522	6.34E+04
132	3290-4034	981.086	1	982.094	556-639	1.01E+05
133	3451-3863	987.073	1	988.081	574-619	1.29E+05
134	3902-4440	1009.070	2	2020.155	624-692	4.74E+05
135	3481-3866	1018.075	2	2038.166	577-619	3.82E+05
136	3903-4230	1020.061	2	2042.138	624-664	1.59E+05
137	3773-3888	1025.083	2	2052.183	609-622	2.09E+05
138	3905-4232	1031.051	2	2064.118	625-665	5.04E+04
139	4269-4408	1085.075	1	1086.083	669-689	1.68E+05
140	3471-3545	1103.086	1	1104.094	576-585	1.05E+05
141	3817-3820	1138.171	2	2278.359	613-614	1.08E+05
142	3818-3821	1149.163	2	2300.342	613-614	6.65E+04
143	3792-3794	1158.101	2	2318.219	611-611	1.08E+05

Table S-26: Strawberry, Screen [VII], T-2RT

Precursor ions present in strawberry leaves classified as ellagitannins under condition [VII] using T-2RT.

No.	Scan No.	*m/z*	Z	Mass	RT (s)	Intensity
1	4940-5111	303.015	1	304.023	601-618	9.34E+04
2	1744947	452.923	1	453.931	1-776	1.85E+05
3	3353-3354	453.540	1	454.548	434-434	5.82E+04
4	3437-3438	469.052	1	470.060	444-445	6.40E+04
5	4589-5936	479.250	1	480.258	565-702	6.64E+05
6	798-1166	481.064	1	482.072	110-150	4.14E+06
7	2316-2318	486.024	1	487.032	293-293	5.01E+04
8	4980-4981	490.039	1	491.047	605-605	1.46E+05
9	3521-4255	491.177	1	492.185	453-533	1.70E+05
10	4984-4987	492.172	1	493.180	605-605	1.24E+05
11	4496-4497	497.260	1	498.268	556-556	5.07E+04
12	6504-6507	541.287	1	542.295	759-759	7.85E+04
13	816-1120	549.050	1	550.058	112-146	1.41E+05
14	4265-4423	551.202	1	552.210	534-549	9.46E+04
15	3448-3449	575.994	1	577.002	446-446	5.12E+04
16	1328-1551	605.078	1	606.086	167-195	7.97E+04
17	4642-4684	611.256	1	612.264	570-574	5.21E+05
18	2991-4668	631.058	1	632.066	392-573	6.86E+05
19	1356-3946	633.074	1	634.082	170-501	5.87E+05
20	808-810	637.067	1	638.075	111-111	4.79E+05
21	814-1564	649.068	1	650.076	112-197	2.14E+05
22	3093-4901	655.056	1	656.064	403-597	2.83E+05
23	2328-3567	663.084	1	664.092	294-459	1.63E+05
24	1098-1101	665.064	1	666.072	144-144	3.33E+05
25	1322-1558	671.050	1	672.058	167-196	8.08E+04
26	1097-1100	687.046	1	688.054	144-144	4.12E+05
27	3109-3789	723.043	1	724.051	405-485	1.35E+05
28	5087-5089	782.061	1	783.069	615-616	7.06E+04
29	1305-5036	783.069	1	784.077	165-610	3.49E+05
30	3585-4173	785.084	1	786.092	461-525	1.69E+05
31	3626-4698	799.064	1	800.072	465-575	6.48E+04
32	1148-3739	801.079	1	802.087	148-478	5.80E+04
33	1960-3216	805.050	1	806.058	247-417	8.64E+04
34	1714-1920	815.095	1	816.103	218-242	2.60E+05
35	1739-2025	831.090	1	832.098	221-255	7.41E+04
36	1753-1784	837.077	1	838.085	223-226	5.77E+04

37	2158-3211	851.056	1	852.064	271-417	2.00E+05
38	2167-3202	873.038	1	874.046	272-416	7.74E+04
39	5627-5628	917.163	1	918.171	670-670	8.49E+04
40	3980-5835	933.064	1	934.072	505-691	5.21E+05
41	4774-4815	935.080	1	936.088	584-588	8.19E+05
42	4862-5278	937.087	1	938.095	593-635	1.25E+05
43	3747-6208	949.059	1	950.067	479-729	1.73E+05
44	2264-5190	951.075	1	952.083	285-626	2.25E+05
45	4389-5812	955.046	1	956.054	545-689	1.09E+05
46	4863-4866	957.062	1	958.070	593-593	9.64E+04
47	815-1108	963.133	1	964.141	112-144	2.23E+05
48	3925-5260	965.091	1	966.099	499-633	1.55E+05
49	2436-4129	973.057	1	974.065	310-521	6.27E+04
50	4482-5597	981.086	1	982.094	555-667	8.67E+04
51	1855-1858	983.101	1	984.109	235-235	5.26E+04
52	4682-5137	987.073	1	988.081	574-620	7.32E+04
53	4791-4793	1003.065	1	1004.073	585-586	5.42E+04
54	5605-5821	1085.075	1	1086.083	667-690	1.06E+05
55	4688-4802	1103.086	1	1104.094	574-587	8.60E+04

Table S-27: Strawberry, Screen [VIII], T-2RT

Precursor ions present in strawberry leaves classified as ellagitannins under condition [VIII] using T-2RT.

No.	Scan No.	*m/z*	Z	Mass	RT (s)	Intensity
1	1625-2169	316.033	2	634.082	401-487	8.86E+05
2	574-3694	391.031	2	784.077	166-680	2.63E+05
3	1976-3232	392.038	2	786.093	460-615	9.55E+04
4	790-895	407.044	2	816.103	221-241	1.15E+05
5	1687-1714	425.024	2	852.065	412-416	6.75E+04
6	1062-1702	436.015	2	874.046	278-414	5.63E+04
7	1723-1724	437.025	2	876.067	417-418	5.09E+04
8	4003-4004	452.050	2	906.115	744-744	2.76E+05
9	1211-2792	453.039	2	908.093	310-572	2.99E+05
10	2003-2004	462.044	2	926.105	464-464	6.25E+04
11	1874-2397	464.030	2	930.075	444-522	1.47E+05
12	3574-3935	465.021	2	932.057	660-726	2.69E+05
13	2134-3776	466.029	2	934.073	481-692	9.18E+05
14	2928-3434	467.036	2	936.088	584-638	2.14E+06
15	4015-4016	467.055	2	936.125	747-747	5.99E+04
16	3436-3495	468.044	2	938.104	638-647	1.25E+05
17	1853-3732	469.052	2	940.120	441-686	1.73E+05
18	1695-1696	470.009	2	942.034	413-413	5.57E+04
19	2683-3933	474.026	2	950.068	559-726	2.28E+05
20	1098-3326	475.034	2	952.083	286-626	2.02E+05
21	3837-3838	475.052	2	952.120	706-707	7.51E+04
22	2256-3506	476.041	2	954.098	504-649	9.37E+04
23	2422-3763	477.019	2	956.055	525-690	2.82E+05
24	3275-3426	477.050	2	956.115	621-637	1.05E+05
25	2935-3416	478.027	2	958.070	585-636	4.37E+05
26	3088-3560	481.034	2	964.083	600-657	2.42E+05
27	1816-3409	482.041	2	966.099	435-635	3.23E+05
28	2339-2341	483.051	2	968.118	515-515	5.22E+04
29	1120-2408	486.024	2	974.065	289-523	1.44E+05
30	2699-3624	490.039	2	982.095	561-668	1.87E+05
31	939-1728	491.028	2	984.072	250-418	1.15E+05
32	800-1917	491.048	2	984.112	223-449	5.98E+04
33	2385-3261	493.033	2	988.081	521-619	1.14E+05
34	1207-2372	497.016	2	996.047	309-519	8.27E+04
35	2594-2778	498.055	2	998.126	547-570	1.29E+05
36	2571-3766	511.013	2	1024.042	545-691	1.05E+05

37	2929-3420	512.021	2	1026.058	584-636	1.33E+05
38	2577-3540	522.004	2	1046.025	545-654	9.33E+04
39	3243-3246	527.027	2	1056.070	616-617	7.34E+04
40	2813-3618	529.044	2	1060.105	574-667	1.29E+05
41	1219-2380	531.009	2	1064.034	311-520	8.41E+04
42	2500-2760	541.055	2	1084.126	535-568	1.12E+05
43	1887-2378	542.000	2	1086.017	445-520	7.24E+04
44	2159-4043	542.034	2	1086.085	485-754	3.93E+05
45	3483-3484	543.042	2	1088.100	645-646	5.16E+04
46	2208-2210	549.053	2	1100.122	494-494	6.10E+04
47	2319-2321	550.060	2	1102.136	513-513	5.70E+04
48	2799-3433	551.040	2	1104.095	572-638	3.22E+05
49	2168-3930	553.025	2	1108.066	487-726	1.82E+05
50	3192-3195	554.044	2	1110.104	612-612	1.01E+05
51	2585-3749	555.998	2	1114.012	546-688	8.00E+04
52	900-1136	556.060	2	1114.135	242-293	6.50E+04
53	3297-3917	558.047	2	1118.110	623-724	1.13E+05
54	2812-3417	562.030	2	1126.076	574-636	1.19E+05
55	2201-2221	563.050	2	1128.116	493-497	6.28E+04
56	3340-3906	569.039	2	1140.094	628-722	9.64E+04
57	2910-2946	573.022	2	1148.059	582-586	8.88E+04
58	3216-3883	594.076	2	1190.168	614-716	5.95E+04
59	3627-3871	598.010	2	1198.035	668-714	7.06E+04
60	2347-2375	598.079	2	1198.173	516-520	4.27E+05
61	2327-2496	616.090	2	1234.195	514-534	8.70E+04
62	2900-3359	617.033	2	1236.081	581-630	1.07E+06
63	3329-3331	625.029	2	1252.074	626-626	5.82E+04
64	2974-3047	628.022	2	1258.061	588-595	5.38E+05
65	626-1221	629.042	2	1260.101	182-311	6.65E+05
66	1690-2826	631.058	2	1264.131	413-575	1.77E+05
67	2296-2463	632.066	2	1266.147	510-531	1.72E+05
68	1807-1810	636.067	2	1274.150	434-435	5.39E+04
69	2989-3035	639.014	2	1280.043	589-594	2.50E+05
70	2305-2336	643.057	2	1288.129	511-515	8.82E+04
71	1817-1820	647.056	2	1296.128	436-436	5.39E+04
72	3391-3392	666.068	2	1334.151	633-633	5.08E+04
73	3056-3059	673.123	2	1348.262	596-596	6.82E+04
74	3023-3027	675.121	2	1352.258	593-593	1.03E+05
75	3024-3028	686.112	2	1374.240	593-593	1.01E+05
76	3673-3958	692.030	2	1386.075	676-731	1.87E+05
77	3061-3410	701.035	2	1404.086	596-635	7.21E+04
78	1650-2530	707.064	2	1416.145	405-540	6.66E+05
79	3720-3747	708.043	2	1418.101	685-688	6.25E+04

80	2473-2474	716.069	2	1434.153	532-532	7.70E+04
81	1928-2114	718.054	2	1438.125	451-478	2.28E+05
82	1959-2543	723.077	2	1448.170	457-541	7.46E+04
83	2008-2094	729.044	2	1460.104	464-476	1.36E+05
84	2022-2087	740.037	2	1482.090	466-475	7.03E+04
85	2954-2957	756.107	2	1514.231	586-587	5.78E+04
86	2153-3346	782.061	2	1566.139	485-628	8.85E+04
87	2209-3119	783.069	2	1568.154	494-605	4.12E+05
88	742-1422	789.050	2	1580.116	210-357	1.56E+05
89	2040-2272	791.067	2	1584.149	468-506	5.32E+04
90	1059-2895	794.060	2	1590.136	277-581	1.45E+05
91	1323-1525	798.055	2	1598.127	333-381	2.06E+05
92	2803-2857	805.051	2	1612.118	573-577	9.41E+04
93	3012-3015	825.116	2	1652.249	592-592	5.49E+04
94	2074-3517	858.067	2	1718.150	473-650	3.03E+05
95	2870-2913	859.075	2	1720.165	578-582	8.70E+04
96	2314-2772	867.072	2	1736.160	512-569	4.22E+05
97	2086-2675	869.057	2	1740.131	475-558	1.14E+05
98	2445-2871	874.080	2	1750.175	529-579	2.30E+05
99	2712-2744	878.062	2	1758.141	562-566	1.33E+05
100	2548-2650	880.048	2	1762.113	542-555	6.34E+04
101	2831-2880	885.071	2	1772.157	575-579	6.67E+04
102	2719-2721	889.054	2	1780.124	563-563	8.73E+04
103	2845-2846	896.062	2	1794.140	576-576	4.42E+04
104	2729-2730	900.044	2	1802.105	564-564	5.56E+04
105	2767-3799	933.064	2	1868.144	569-696	4.51E+05
106	3064-3361	934.073	2	1870.161	597-630	2.50E+06
107	2205-2225	941.061	2	1884.138	494-497	7.47E+04
108	2689-3388	942.069	2	1886.153	560-633	5.64E+04
109	2832-2835	944.056	2	1890.128	575-576	7.26E+04
110	3037-3369	945.062	2	1892.140	594-631	3.49E+05
111	3026-3029	945.565	2	1893.146	593-593	4.35E+04
112	2952-2956	946.070	2	1894.156	586-586	7.57E+04
113	1515-2113	949.059	2	1900.134	379-478	1.28E+05
114	3196-3213	953.044	2	1908.103	612-614	6.78E+04
115	813-3375	956.052	2	1914.119	225-631	1.62E+05
116	1678-1749	957.055	2	1916.126	411-422	7.68E+04
117	1522-1541	958.064	2	1918.143	381-384	9.77E+04
118	2041-2043	960.051	2	1922.118	468-469	5.03E+04
119	807-1673	965.053	2	1932.123	224-410	1.31E+05
120	3181-3185	967.042	2	1936.100	611-611	1.00E+05
121	1744-1860	972.061	2	1946.138	422-442	1.19E+05
122	2016-2017	996.137	2	1994.290	465-465	5.68E+04

123	3280-3926	1009.070	2	2020.155	621-725	2.26E+05
124	2807-3411	1018.075	2	2038.166	573-636	1.40E+05
125	3286-3606	1020.061	2	2042.138	622-665	9.80E+04
126	3137-3760	1025.084	2	2052.184	607-690	1.03E+05
127	2814-2864	1029.065	2	2060.147	574-578	7.08E+04
128	3290-3591	1031.052	2	2064.120	622-663	5.21E+04
129	3281-3283	1036.075	2	2074.165	621-621	4.74E+04
130	3189-3193	1138.172	2	2278.360	612-612	2.08E+05
131	3191-3194	1149.164	2	2300.344	612-612	1.16E+05
132	3163-3166	1158.103	2	2318.223	609-609	5.36E+04
133	3204-3205	1160.157	2	2322.330	613-613	6.52E+04
134	3173-3176	1169.094	2	2340.204	610-610	7.42E+04

Table S-28: Strawberry, Screen [IX], T-2RT

Precursor ions present in strawberry leaves classified as ellagitannins under condition [IX] using T-2RT.

No.	Scan No.	*m/z*	Z	Mass	RT (s)	Intensity
1	2278-2298	316.033	2	634.082	480-484	1.96E+06
2	1296-1929	391.031	2	784.077	271-418	8.14E+05
3	4036-4037	452.050	2	906.115	744-744	2.91E+05
4	1452-2583	453.039	2	908.093	311-530	4.48E+05
5	2630-3380	453.104	1	454.112	536-632	1.39E+06
6	2070-2515	464.030	2	930.075	446-520	2.68E+05
7	2541-3742	466.029	2	934.073	523-690	1.55E+06
8	2993-3400	467.036	2	936.088	583-635	2.50E+06
9	1438-2538	475.034	2	952.083	308-523	4.69E+05
10	2692-3730	477.019	2	956.054	545-688	5.74E+05
11	3193-3195	477.521	1	478.529	606-606	2.75E+05
12	3003-3385	478.027	2	958.070	584-633	5.94E+05
13	2847-3699	479.250	1	480.258	566-683	1.04E+06
14	3230-3233	481.034	2	964.083	611-611	3.12E+05
15	504-708	481.063	1	482.071	110-145	4.12E+06
16	2390-3405	482.041	2	966.099	498-635	4.63E+05
17	1451-2514	486.024	2	974.065	311-520	2.78E+05
18	3276-3278	493.032	2	988.081	617-617	2.71E+05
19	2856-2857	498.055	2	998.126	567-567	2.57E+05
20	2917-2918	511.013	2	1024.042	574-574	2.53E+05
21	3004-3007	512.021	2	1026.058	584-585	2.71E+05
22	2985-2986	529.044	2	1060.105	582-582	3.13E+05
23	2755-2756	541.055	2	1084.126	554-554	3.17E+05
24	3592-3964	542.034	2	1086.085	667-732	6.34E+05
25	676-677	549.050	1	550.058	142-142	3.79E+05
26	2904-3406	551.039	2	1104.095	573-636	5.64E+05
27	3711-3865	553.025	2	1108.066	686-713	2.70E+05
28	3348-3915	558.047	2	1118.110	627-722	2.79E+05
29	2988-2989	562.030	2	1126.076	582-583	3.53E+05
30	519-521	566.041	1	567.049	111-111	2.87E+05
31	2488-2513	598.078	2	1198.173	517-520	3.71E+05
32	3027-3128	617.033	2	1236.081	587-598	2.46E+06
33	3036-3066	628.022	2	1258.061	589-592	8.09E+05
34	1905-2063	631.058	2	1264.131	414-445	5.79E+05
35	1789-2869	631.058	1	632.066	393-568	1.12E+06
36	2450-2472	632.066	2	1266.147	510-514	3.36E+05

37	1595-2318	633.074	1	634.082	348-487	1.41E+06
38	517-520	637.067	1	638.075	111-111	4.12E+05
39	531-962	649.068	1	650.076	112-197	3.47E+05
40	1863-3084	655.056	1	656.064	406-594	4.29E+05
41	1826-2138	663.084	1	664.092	400-457	2.75E+05
42	697-699	665.064	1	666.072	144-144	2.96E+05
43	3079-3080	675.121	2	1352.257	593-593	2.56E+05
44	695-698	687.046	1	688.054	144-144	3.66E+05
45	3674-3739	692.030	2	1386.075	680-690	3.50E+05
46	2097-2250	707.064	2	1416.144	450-475	1.84E+06
47	2182-2245	718.054	2	1438.124	465-474	4.55E+05
48	2230-2231	729.045	2	1460.106	472-472	2.56E+05
49	1187-2153	783.069	1	784.077	247-460	1.23E+06
50	2622-2966	783.069	2	1568.154	535-579	9.57E+05
51	2521-2524	785.085	1	786.093	521-522	2.60E+05
52	2905-2942	794.060	2	1590.136	573-576	3.86E+05
53	1554-1579	798.055	2	1598.127	337-344	3.22E+05
54	1064-1135	815.095	1	816.103	222-236	3.39E+05
55	1310-1919	851.056	1	852.064	274-416	2.87E+05
56	2239-2831	858.067	2	1718.150	474-564	4.96E+05
57	2819-2843	867.072	2	1736.160	562-565	7.49E+05
58	2666-2769	869.057	2	1740.130	541-555	3.05E+05
59	2922-2957	874.080	2	1750.175	574-578	4.21E+05
60	2826-2828	878.062	2	1758.140	563-563	2.91E+05
61	3192-3194	933.064	2	1868.144	606-606	1.51E+06
62	2684-3748	933.064	1	934.072	544-691	8.08E+05
63	3114-3366	934.073	2	1870.161	597-630	2.64E+06
64	2995-3024	935.079	1	936.087	584-587	8.19E+05
65	3074-3076	937.087	1	938.095	593-593	3.42E+05
66	3083-3252	945.062	2	1892.140	594-614	6.23E+05
67	3605-3929	949.059	1	950.067	669-725	2.68E+05
68	2201-2202	949.059	2	1900.135	468-468	2.60E+05
69	1444-2533	951.074	1	952.082	309-522	3.83E+05
70	3231-3234	956.052	2	1914.120	611-611	3.10E+05
71	525-526	963.133	1	964.141	111-112	2.71E+05
72	2397-3291	965.091	1	966.099	500-619	3.89E+05
73	3306-3578	1009.070	2	2020.155	621-665	5.49E+05
74	2914-2948	1018.075	2	2038.166	573-577	4.16E+05
75	3316-3317	1020.060	2	2042.136	623-623	2.78E+05
76	3302-3303	1025.083	2	2052.182	621-621	2.59E+05
77	3600-3601	1085.075	1	1086.083	668-668	2.70E+05

Table S-29: Strawberry, Screen [X], T-2RT

Precursor ions present in strawberry leaves classified as ellagitannins under condition [X] using T-2RT.

No.	Scan No.	*m/z*	Z	Mass	RT (s)	Intensity
1	514-716	481.063	1	482.071	110-146	4.14E+06
2	686-688	549.050	1	550.058	142-142	5.90E+05
3	524-527	566.041	1	567.049	111-111	3.13E+05
4	1753-2678	631.058	1	632.066	395-569	1.41E+06
5	1571-2283	633.074	1	634.082	350-499	1.32E+06
6	523-526	637.067	1	638.075	111-111	3.33E+05
7	534-971	649.068	1	650.076	112-198	3.47E+05
8	1813-2822	655.056	1	656.064	407-594	5.46E+05
9	1782-2060	663.084	1	664.092	401-457	2.76E+05
10	700-703	665.064	1	666.072	144-144	3.46E+05
11	699-702	687.046	1	688.054	143-144	4.12E+05
12	2185-2186	723.043	1	724.051	482-482	2.59E+05
13	1177-2074	783.069	1	784.077	247-461	1.20E+06
14	2397-2400	785.085	1	786.093	522-522	3.04E+05
15	1061-1126	815.095	1	816.103	223-237	3.42E+05
16	1303-1862	851.056	1	852.064	275-417	2.89E+05
17	2532-3390	933.064	1	934.072	544-691	8.15E+05
18	2764-2782	935.080	1	936.088	584-587	8.31E+05
19	2814-2816	937.087	1	938.095	592-593	2.73E+05
20	3560-3561	949.059	1	950.067	724-724	2.55E+05
21	1435-2407	951.074	1	952.082	311-523	3.86E+05
22	530-704	963.133	1	964.141	111-144	2.89E+05
23	2289-2994	965.090	1	966.098	500-619	3.94E+05
24	3264-3265	1085.075	1	1086.083	668-668	2.55E+05

Table S-30: Strawberry, Screen [XI], T-2RT

Precursor ions present in strawberry leaves classified as ellagitannins under condition [XI] using T-2RT.

No.	Scan No.	*m/z*	Z	Mass	RT (s)	Intensity
1	1719-1747	316.033	2	634.082	480-487	1.41E+06
2	925-1460	391.031	2	784.077	271-418	8.10E+05
3	2914-2915	452.050	2	906.115	744-744	2.92E+05
4	1065-1925	453.039	2	908.093	311-530	4.63E+05
5	1572-1880	464.030	2	930.075	446-520	2.69E+05
6	1898-2719	466.029	2	934.074	523-690	1.69E+06
7	2214-2497	467.036	2	936.088	584-635	2.44E+06
8	1054-1894	475.034	2	952.083	308-523	4.48E+05
9	1994-2723	477.019	2	956.054	545-691	5.15E+05
10	2217-2488	478.027	2	958.070	584-633	5.49E+05
11	2365-2368	481.034	2	964.084	609-610	3.02E+05
12	1791-2500	482.041	2	966.099	498-636	4.82E+05
13	1066-1883	486.024	2	974.065	311-521	2.67E+05
14	2409-2410	493.033	2	988.081	617-617	2.57E+05
15	2109-2110	498.055	2	998.125	568-568	3.19E+05
16	2151-2153	511.013	2	1024.042	575-575	2.54E+05
17	2221-2223	512.021	2	1026.058	585-585	2.68E+05
18	2204-2206	529.044	2	1060.105	582-583	3.79E+05
19	2037-2038	541.055	2	1084.126	554-554	2.99E+05
20	2621-2872	542.034	2	1086.085	667-732	6.30E+05
21	2136-2503	551.039	2	1104.095	573-636	5.94E+05
22	2693-2801	553.025	2	1108.066	686-713	2.52E+05
23	2463-2833	558.047	2	1118.110	627-722	2.84E+05
24	2205-2207	562.030	2	1126.077	583-583	3.12E+05
25	1860-1878	598.079	2	1198.173	517-520	3.73E+05
26	2238-2304	617.033	2	1236.081	588-598	2.03E+06
27	2243-2269	628.022	2	1258.061	588-592	5.16E+05
28	1451-1696	631.058	2	1264.132	415-475	5.44E+05
29	1834-1849	632.066	2	1266.147	510-514	3.71E+05
30	2670-2716	692.030	2	1386.075	680-690	3.35E+05
31	1589-1711	707.064	2	1416.145	450-478	1.38E+06
32	1644-1693	718.054	2	1438.125	465-475	4.13E+05
33	1679-1680	729.045	2	1460.107	472-472	2.76E+05
34	1948-2185	783.069	2	1568.155	535-579	1.01E+06
35	2137-2167	794.060	2	1590.135	573-576	3.95E+05
36	1168-1193	798.055	2	1598.127	338-345	3.18E+05

37	1687-2088	858.067	2	1718.150	474-564	5.06E+05
38	2188-2189	859.074	2	1720.165	579-580	2.54E+05
39	2080-2102	867.072	2	1736.160	562-566	6.72E+05
40	1975-2043	869.057	2	1740.130	542-555	2.94E+05
41	2150-2178	874.079	2	1750.175	574-578	4.78E+05
42	2085-2087	878.062	2	1758.140	563-563	2.63E+05
43	2342-2343	933.064	2	1868.144	606-606	1.49E+06
44	2296-2475	934.073	2	1870.163	597-630	3.28E+06
45	2279-2390	945.062	2	1892.140	594-613	5.30E+05
46	1661-1662	949.059	2	1900.135	468-468	3.13E+05
47	2375-2376	956.052	2	1914.121	610-611	4.22E+05
48	2434-2612	1009.070	2	2020.155	622-665	5.10E+05
49	2142-2172	1018.075	2	2038.166	573-577	3.76E+05
50	2443-2444	1020.060	2	2042.136	623-624	2.71E+05
51	2358-2431	1025.083	2	2052.183	608-621	2.61E+05
52	2385-2386	1138.172	2	2278.360	612-612	2.44E+05

Table S-31: Strawberry, Summary of Screens [I-XI], T-3RT

Precursor ions present in strawberry leaves classified as ellagitannins under conditions [I-XI] using T-3RT.

T-3RT	I.	II.	III.	IV.	V.	VI.	VII.	VIII.	IX.	X	XI.
Anions	13	14	0	**23**	**23**	16	22	0	16	15	0
< 5e5 Intensity	6	6	0	19	**20**	11	18	0	9	10	0
> 5e5 Intensity	7	**8**	0	4	3	5	4	0	7	5	0
< 900 Da	10	10	0	15	**17**	11	14	0	11	10	0
< 1100 Da	13	14	0	**22**	**22**	16	21	0	16	15	0
>1100 Da	0	0	0	1	1	0	1	0	0	0	0
Dianions	20	0	**29**	20	25	18	0	20	14	0	12
< 5e5 Intensity	5	0	**16**	14	15	11	0	13	5	0	3
> 5e5 Intensity	**15**	0	13	6	10	7	0	7	9	0	9
< 900 Da	1	0	**4**	1	3	2	0	1	0	0	0
< 1100 Da	10	0	**16**	8	14	11	0	12	7	0	7
>1100 Da	10	0	**13**	12	11	7	0	8	7	0	5
Total	33	14	29	43	**48**	34	22	20	30	15	12
< 5e5 Intensity	11	6	16	33	**35**	22	18	13	14	10	3
> 5e5 Intensity	**22**	8	13	10	13	12	4	7	16	5	9
Unique Mass	29	14	29	38	**42**	29	22	20	27	15	12

*I. (Intensity), II. (Anion), III. (Dianion), IV. (DE 12s), V. (DE 3s), VI. (DE 3s, Apex), VII. (-1, DE 3s), VIII. (-2, DE 3s), IX. (DE 3s, 5e5), X. (-1, DE 3s, 5e5), XI. (-2, DE 3s, 5e5)

Table S-32: Referenced Annotated Ellagitannins

Concatenation of previously reported species classified as ellagitannins in strawberry leaves, strawberry fruit, and non-strawberry plant sources and associated references.

Plant Source	Ref.	E No.	Ellagitannin	MW	Mass (Da)	CID Fragment (*m/z*)	HCD NCE 20 Fragments (*m/z*)
Strawberry Leaves	64	E1	2,3-(S)-HHDP-glucose		482.070	301.00	
Strawberry Leaves	64	E3	galloyl-HHDP-glucose a		634.081	300.92	
Strawberry Leaves	64	E4	galloyl-HHDP-glucose b		634.081	300.92	
Strawberry Leaves	64	E5	galloyl-HHDP-glucose c		634.081	300.92	
Strawberry Leaves	64	E2	pedunculagin		784.076	301.04	
Strawberry Leaves	64	E6	tellimagrandin I		786.092	300.97	
Strawberry Leaves	64	E7	dimeric ellagitannin 1416 Da		1416.141	300.97	
Strawberry Leaves	64	E8	dimeric ellagitannin 1718 Da a		1718.147	300.96	
Strawberry Leaves	64	E9	dimeric ellagitannin 1718 Da b		1718.147	300.96	
Strawberry Leaves	64	E10	dimeric ellagitannin 1718 Da c		1718.147	300.96	
Strawberry Leaves	64	E17	dimeric ellagitannin 1870 Da		1870.158	300.99	
Strawberry Leaves	64	E18	agrimoniin		1870.158	300.99	
Strawberry Leaves	73	E24	Agrimoniin	1871			
Strawberry Leaves	64	E11	dimeric ellagitannin 2020 Da a		2020.153	300.99	
Strawberry Leaves	64	E12	dimeric ellagitannin 2020 Da b		2020.153	300.99	
Strawberry Leaves	64	E13	dimeric ellagitannin 2020 Da c		2020.153	300.99	
Strawberry Leaves	64	E14	dimeric ellagitannin 2020 Da d		2020.153	300.99	
Strawberry Leaves	64	E15	dimeric ellagitannin 2038 Da a		2038.164	300.96	
Strawberry Leaves	64	E16	dimeric ellagitannin 2038 Da b		2038.164	300.96	
Strawberry Leaves	64	E19	trimeric ellagitannin 2502 Da		2502.223	301.00	
Strawberry Leaves	64	E20	trimeric ellagitannin 2804 Da		2804.229	300.99	
Strawberry Fruit	62	E3	Galloyl pentose		302.065		169.013; 149.995; 125.023
Strawberry Fruit	72	E5	Galloyl glucose		332.111	313; 289; 287; 271; 235; 203; 169; 165; 127	
Strawberry Fruit	62	E16	Digalloyl hexose		484.087		331.067; 313.057
Strawberry Fruit	62	E4	Galloyl-HHDP-hexose		634.081		633.073; 481.062; 463.051; 331.067; 300.999; 275.020
Strawberry Fruit	62	E5	Galloyl-HHDP-hexose		634.081		633.073; 481.062; 463.051; 331.067; 300.999; 275.020
Strawberry Fruit	62	E8	Galloyl-HHDP-hexose		634.081		633.073; 481.062; 463.051; 331.067; 300.999; 275.020
Strawberry Fruit	72	E6	Trigalloyl glucose		636.096	465	
Strawberry Fruit	62	E17	Trigalloyl hexose		636.099		465.067; 313.056; 483.077;
Strawberry Fruit	72	E1	Bis-HHDP-glucose		784.075	481; 301; 275	
Strawberry Fruit	62	E7	Digalloyl-HHDP-hexose		786.092		633.074; 615.063; 463.053; 483.078; 313.057; 300.999
Strawberry Fruit	62	E13	Digalloyl-HHDP-hexose		786.092		633.074; 615.063; 463.053; 483.078; 313.057; 300.999

Strawberry Fruit	62	E27	Tetragalloyl hexose		788.111		787.096; 635.088; 617.078; 465.068; 300.999; 169.013
Strawberry Fruit	62	E9	Galloyl-HHDP-hexose-HHDP (castalagin, vescalagin)		934.073		915.051; 631.058; 479.046; 461.038; 300.999
Strawberry Fruit	62	E10	Galloyl-HHDP-hexose-HHDP (castalagin, vescalagin)		934.073		915.051; 631.058; 479.046; 461.038; 300.999
Strawberry Fruit	62	E12	Galloyl-HHDP-hexose-HHDP (castalagin, vescalagin)		934.073		915.051; 631.058; 479.046; 461.038; 300.999
Strawberry Fruit	62	E19	Galloyl-HHDP-hexose-HHDP (castalagin, vescalagin)		934.074		915.051; 631.058; 479.046; 461.038; 300.999
Strawberry Fruit	72	E7	Galloyl bis-hexahydroxydiphenoyl (HHDP)-glucose		936.085	633; 301	
Strawberry Fruit	62	E20	Galloyl-diHHDP-hexose (casuarictin, potentillin)		936.086		935.081; 783.069; 765.057; 633.073; 463.052; 300.999
Strawberry Fruit	62	E21	Galloyl-diHHDP-hexose (casuarictin, potentillin)		936.086		935.081; 783.069; 765.057; 633.073; 463.052; 300.999
Strawberry Fruit	62	E26	Galloyl-diHHDP-hexose (casuarictin, potentillin)		936.086		935.081; 783.069; 765.057; 633.073; 463.052; 300.999
Strawberry Fruit	62	E22	Trigalloyl-HHDP hexose		938.103		937.095, 785.079, 767.074, 635.198; 465.067, 300.999
Strawberry Fruit	62	E23	Trigalloyl-HHDP hexose		938.103		937.095, 785.079, 767.074, 635.198; 465.067, 300.999
Strawberry Fruit	62	E29	Pentagalloyl hexose		940.126		939.112; 787.098; 769.089; 617.080; 599.070; 447.059; 300.999; 169.014
Strawberry Fruit	72	E2	Unknown Ellagitannin		948.051	929; 901; 883; 875	
Strawberry Fruit	62	E1	Galloyl-HHDP-DHHDP-hexose (geraniin)		952.083		907.085; 781.054; 605.079; 479; 425.025; 298; 273.004;
Strawberry Fruit	62	E2	Galloyl-HHDP-DHHDP-hexose (geraniin)		952.083		907.085; 781.054; 605.079; 479; 425.025; 298; 273.004;
Strawberry Fruit	62	E6	Galloyl-HHDP-DHHDP-hexose (geraniin)		952.083		907.085; 781.054; 605.079; 479; 425.025; 298; 273.004;
Strawberry Fruit	62	E11	Galloyl-HHDP-DHHDP-hexose (geraniin)		952.083		907.085; 781.054; 605.079; 479; 425.025; 298; 273.004;
Strawberry Fruit	62	E14	Galloyl-HHDP-DHHDP-hexose (geraniin)		952.083		907.085; 781.054; 605.079; 479; 425.025; 298; 273.004;
Strawberry Fruit	62	E15	Galloyl-HHDP-DHHDP-hexose (geraniin)		952.083		907.085; 781.054; 605.079; 479; 425.025; 298; 273.004;
Strawberry Fruit	62	E18	Galloyl-HHDP-DHHDP-hexose (geraniin)		952.083		907.085; 781.054; 605.079; 479; 425.025; 298; 273.004;
Strawberry Fruit	62	E24	Sanguiin h10		1568.150		1415.141; 1265.137; 1103.059; 1085.076; 633.074; 935.081; 783.069; 481.061
Strawberry Fruit	62	E25	Sanguiin h10		1568.150		1415.141; 1265.137; 1103.059; 1085.076; 633.074; 935.081; 783.069; 481.061
Strawberry Fruit	62	E28	Galloyl-diHHDP-hexose dimer		1870.146		1869.143; 1567.152; 1265.140; 1085.076; 935.082; 783.068; 633.074; 613.049; 481.064; 301.000
Strawberry Fruit	72	E3	Unknown Ellagitannin		1918.139	1557; 1224; 1099; 1096; 986; 967	
Strawberry Fruit	72	E4	Unknown Ellagitannin		1934.135	1859; 948; 947; 942; 929; 921; 920	
Not Strawberry	73	E21	Isostrictinin	634			
Not Strawberry	74	E6	Isostrictinin		634.08		
Not Strawberry	74	E9	Corilagin		634.08		
Not Strawberry	74	E10	Chebulanin		652.09		
Not Strawberry	73	E20	Pedunculagin	784			
Not Strawberry	74	E16	Pedunculagin		784.08		
Not Strawberry	32	E15	pedunculagin	784.5		301	
Not Strawberry	73	E22	Tellimagrandin I	786			
Not Strawberry	74	E18	Tellimagrandin I		786.09		
Not Strawberry	32	E1	tellimagrandin I	786.6		301	
Not Strawberry	73	E1	Vescalagin	934			
Not Strawberry	73	E4	Castalagin	934			
Not Strawberry	74	E3	Vescalagin		934.07		
Not Strawberry	74	E4	Castalagin		934.07		
Not Strawberry	32	E8	vescalagin	934.6		301	

Not Strawberry	32	E9	castalagin	934.6		301	
Not Strawberry	73	E5	Stachyurin	936			
Not Strawberry	73	E9	Casuarinin	936			
Not Strawberry	73	E19	Casuarictin	936			
Not Strawberry	74	E5	Stachyurin		936.09		
Not Strawberry	74	E8	Casuarinin		936.09		
Not Strawberry	74	E12	Casuarictin		936.09		
Not Strawberry	74	E15	PGG		940.12		
Not Strawberry	73	E3	Methyl derivative of vescalagin	948			
Not Strawberry	73	E7	Methyl derivative of stachyurin	950			
Not Strawberry	74	E11	Geraniin		952.08		
Not Strawberry	32	E18	geraniin	952.6		301	
Not Strawberry	74	E13	Chebulagic acid		954.10		
Not Strawberry	32	E16	chebulagic acid	954.7		301	
Not Strawberry	74	E14	Chebulinic acid		956.11		
Not Strawberry	73	E8	Pterocarinin A	1068			
Not Strawberry	74	E17	Punicalagin		1084.07		
Not Strawberry	32	E17	punicalagin	1084.7		301	
Not Strawberry	73	E10	Vescavaloninic acid	1102			
Not Strawberry	73	E11	Castavaloninic acid	1102			
Not Strawberry	74	E1	Vescavaloninic acid		1102.08		
Not Strawberry	74	E2	Castavaloninic acid		1102.08		
Not Strawberry	32	E10	vescavaloninic acid	1102.7		301	
Not Strawberry	32	E11	castavaloninic acid	1102.7		301	
Not Strawberry	73	E12	Hippophaenin B	1104			
Not Strawberry	74	E7	Hippophaenin B		1104.09		
Not Strawberry	73	E14	Methyl derivative of hippophaenin B	1118			
Not Strawberry	73	E2	Catechin derivative of vescalagin	1206			
Not Strawberry	73	E6	Catechin derivative of stachyurin	1208			
Not Strawberry	73	E15	Elaeagnatin A	1236			
Not Strawberry	73	E13	Catechin derivative of hippophaenin B	1376			
Not Strawberry	74	E19	Oenothein B		1568.15		
Not Strawberry	73	E27	Oenothein B	1569			
Not Strawberry	32	E2	oenothein B	1569.1		765;301	
Not Strawberry	32	E13	sanguiin H10	1569.1		301	
Not Strawberry	73	E16	Roburin A	1851			
Not Strawberry	73	E17	Methyl derivative of roburin A	1865			
Not Strawberry	74	E20	Cocciferin D2		1868.14		
Not Strawberry	73	E18	Cocciferin D2	1869			
Not Strawberry	32	E12	cocciferin D2	1869.3		301	
Not Strawberry	74	E22	Sanguiin H-6		1870.16		

Not Strawberry	74	E23	Agrimoniin		1870.16		
Not Strawberry	73	E26	Sanguiin H-6	1871			
Not Strawberry	32	E4	sanguiin H6	1871.3		301	
Not Strawberry	32	E6	agrimoniin	1871.3		301	
Not Strawberry	73	E23	Gemin A	1873			
Not Strawberry	32	E7	gemin A	1873.3		301	
Not Strawberry	32	E14	rugosin D	1875.3		301	
Not Strawberry	32	E3	oenothein A	2353.6		301	
Not Strawberry	74	E21	Lambertianin C		2804.23		
Not Strawberry	73	E25	Lambertainin C	2805			
Not Strawberry	32	E5	lambertianin C	2805.9		301	

Table S-33: Screen [IV], T-2RT Comparison Match Strawberry Leaves

Comparison of 154 non-isomeric ellagitannins (NIE's) present in strawberry leaves with 11 previously reported species in the same plant tissue.

NIE No.	Matched Fragment(s) (*m/z*)	Precursor Δ (Da)	Mass (Da)		Reported Mass (Da)	Reported Fragment (*m/z*)	Ref.	E No.	Ellagitannin
2R_IV_NIE_1	300.999	0.002	482.072		482.070	301.00	64	E1	2,3-(S)-HHDP-glucose
2R_IV_NIE_12	300.999; 300.918	0	634.081		634.081	300.92	64	E3, E4, E5	galloyl-HHDP-glucose a-c
2R_IV_NIE_22	300.999	0.001	784.077		784.076	301.04	64	E2	pedunculagin
2R_IV_NIE_23	300.998	0	786.092		786.092	300.97	64	E6	tellimagrandin I
2R_IV_NIE_105	300.998	0.004	1416.145		1416.141	300.97	64	E7	dimeric ellagitannin 1416 Da
2R_IV_NIE_119	300.998	0.003	1718.150		1718.147	300.96	64	E8, E9, E10	dimeric ellagitannin 1718 Da a-c
2R_IV_NIE_132	300.999	0.001	1870.159		1870.158	300.99	73;64	E24; E17, E18	agrimoniin, dimeric ellagitannin 1870 Da
2R_IV_NIE_146	300.999	0.003	2020.156		2020.153	300.99	64	E11, E12, E13, E14	dimeric ellagitannin 2020 Da a-d
2R_IV_NIE_147	300.999	0.003	2038.167		2038.164	300.96	64	E15, E16	dimeric ellagitannin 2038 Da a-b
					2502.223	301.00	64	E19	trimeric ellagitannin 2502 Da
					2804.229	300.99	64	E20	trimeric ellagitannin 2804 Da
2R_IV_NIE_2	N/A	N/A	491.047						
2R_IV_NIE_3	N/A	N/A	492.185						
2R_IV_NIE_4	N/A	N/A	494.040						
2R_IV_NIE_5	N/A	N/A	510.066						
2R_IV_NIE_6	N/A	N/A	524.211						
2R_IV_NIE_7	N/A	N/A	542.295						
2R_IV_NIE_8	N/A	N/A	550.058						
2R_IV_NIE_9	N/A	N/A	580.039						
2R_IV_NIE_10	N/A	N/A	606.086						
2R_IV_NIE_11	N/A	N/A	632.066						
2R_IV_NIE_13	N/A	N/A	638.075						
2R_IV_NIE_14	N/A	N/A	650.076						
2R_IV_NIE_15	N/A	N/A	656.063						
2R_IV_NIE_16	N/A	N/A	664.091						
2R_IV_NIE_17	N/A	N/A	666.072						
2R_IV_NIE_18	N/A	N/A	672.058						
2R_IV_NIE_19	N/A	N/A	682.066						
2R_IV_NIE_20	N/A	N/A	688.054						
2R_IV_NIE_21	N/A	N/A	724.051						
2R_IV_NIE_24	N/A	N/A	800.072						
2R_IV_NIE_25	N/A	N/A	802.087						
2R_IV_NIE_26	N/A	N/A	806.058						
2R_IV_NIE_27	N/A	N/A	816.103						
2R_IV_NIE_28	N/A	N/A	832.098						
2R_IV_NIE_29	N/A	N/A	838.084						
2R_IV_NIE_30	N/A	N/A	852.064						
2R_IV_NIE_31	N/A	N/A	874.045						
2R_IV_NIE_32	N/A	N/A	876.067						
2R_IV_NIE_33	N/A	N/A	906.115						
2R_IV_NIE_34	N/A	N/A	908.093						
2R_IV_NIE_35	N/A	N/A	918.171						
2R_IV_NIE_36	N/A	N/A	922.073						
2R_IV_NIE_37	N/A	N/A	926.105						

2R_IV_NIE_38	N/A	N/A	930.075
2R_IV_NIE_39	N/A	N/A	934.072
2R_IV_NIE_40	N/A	N/A	935.075
2R_IV_NIE_41	N/A	N/A	936.087
2R_IV_NIE_42	N/A	N/A	936.125
2R_IV_NIE_43	N/A	N/A	938.096
2R_IV_NIE_44	N/A	N/A	938.103
2R_IV_NIE_45	N/A	N/A	940.120
2R_IV_NIE_46	N/A	N/A	942.034
2R_IV_NIE_47	N/A	N/A	950.068
2R_IV_NIE_48	N/A	N/A	952.082
2R_IV_NIE_49	N/A	N/A	954.098
2R_IV_NIE_50	N/A	N/A	956.054
2R_IV_NIE_51	N/A	N/A	956.115
2R_IV_NIE_52	N/A	N/A	958.070
2R_IV_NIE_53	N/A	N/A	964.083
2R_IV_NIE_54	N/A	N/A	964.141
2R_IV_NIE_55	N/A	N/A	966.099
2R_IV_NIE_56	N/A	N/A	968.118
2R_IV_NIE_57	N/A	N/A	974.065
2R_IV_NIE_58	N/A	N/A	982.094
2R_IV_NIE_59	N/A	N/A	984.071
2R_IV_NIE_60	N/A	N/A	984.111
2R_IV_NIE_61	N/A	N/A	988.081
2R_IV_NIE_62	N/A	N/A	996.047
2R_IV_NIE_63	N/A	N/A	998.126
2R_IV_NIE_64	N/A	N/A	1004.075
2R_IV_NIE_65	N/A	N/A	1024.042
2R_IV_NIE_66	N/A	N/A	1026.057
2R_IV_NIE_67	N/A	N/A	1046.025
2R_IV_NIE_68	N/A	N/A	1056.070
2R_IV_NIE_69	N/A	N/A	1060.105
2R_IV_NIE_70	N/A	N/A	1064.034
2R_IV_NIE_71	N/A	N/A	1082.086
2R_IV_NIE_72	N/A	N/A	1084.126
2R_IV_NIE_73	N/A	N/A	1086.017
2R_IV_NIE_74	N/A	N/A	1086.084
2R_IV_NIE_75	N/A	N/A	1098.142
2R_IV_NIE_76	N/A	N/A	1100.122
2R_IV_NIE_77	N/A	N/A	1104.094
2R_IV_NIE_78	N/A	N/A	1108.066
2R_IV_NIE_79	N/A	N/A	1110.104
2R_IV_NIE_80	N/A	N/A	1114.012
2R_IV_NIE_81	N/A	N/A	1114.136
2R_IV_NIE_82	N/A	N/A	1118.110
2R_IV_NIE_83	N/A	N/A	1126.077
2R_IV_NIE_84	N/A	N/A	1128.117
2R_IV_NIE_85	N/A	N/A	1130.047
2R_IV_NIE_86	N/A	N/A	1140.092
2R_IV_NIE_87	N/A	N/A	1148.059
2R_IV_NIE_88	N/A	N/A	1190.168
2R_IV_NIE_89	N/A	N/A	1198.173
2R_IV_NIE_90	N/A	N/A	1216.047
2R_IV_NIE_91	N/A	N/A	1234.196
2R_IV_NIE_92	N/A	N/A	1236.080
2R_IV_NIE_93	N/A	N/A	1252.073

2R_IV_NIE_94	N/A	N/A	1258.060
2R_IV_NIE_95	N/A	N/A	1264.131
2R_IV_NIE_96	N/A	N/A	1266.145
2R_IV_NIE_97	N/A	N/A	1288.129
2R_IV_NIE_98	N/A	N/A	1296.128
2R_IV_NIE_99	N/A	N/A	1334.152
2R_IV_NIE_100	N/A	N/A	1348.263
2R_IV_NIE_101	N/A	N/A	1352.257
2R_IV_NIE_102	N/A	N/A	1374.240
2R_IV_NIE_103	N/A	N/A	1386.075
2R_IV_NIE_104	N/A	N/A	1404.086
2R_IV_NIE_106	N/A	N/A	1418.102
2R_IV_NIE_107	N/A	N/A	1434.153
2R_IV_NIE_108	N/A	N/A	1438.124
2R_IV_NIE_109	N/A	N/A	1448.170
2R_IV_NIE_110	N/A	N/A	1460.102
2R_IV_NIE_111	N/A	N/A	1482.090
2R_IV_NIE_112	N/A	N/A	1514.233
2R_IV_NIE_113	N/A	N/A	1566.139
2R_IV_NIE_114	N/A	N/A	1568.155
2R_IV_NIE_115	N/A	N/A	1584.149
2R_IV_NIE_116	N/A	N/A	1590.135
2R_IV_NIE_117	N/A	N/A	1598.126
2R_IV_NIE_118	N/A	N/A	1612.117
2R_IV_NIE_120	N/A	N/A	1720.166
2R_IV_NIE_121	N/A	N/A	1736.159
2R_IV_NIE_122	N/A	N/A	1738.165
2R_IV_NIE_123	N/A	N/A	1740.130
2R_IV_NIE_124	N/A	N/A	1750.175
2R_IV_NIE_125	N/A	N/A	1758.141
2R_IV_NIE_126	N/A	N/A	1762.114
2R_IV_NIE_127	N/A	N/A	1772.159
2R_IV_NIE_128	N/A	N/A	1780.123
2R_IV_NIE_129	N/A	N/A	1784.094
2R_IV_NIE_130	N/A	N/A	1794.139
2R_IV_NIE_131	N/A	N/A	1868.144
2R_IV_NIE_133	N/A	N/A	1884.140
2R_IV_NIE_134	N/A	N/A	1886.154
2R_IV_NIE_135	N/A	N/A	1890.125
2R_IV_NIE_136	N/A	N/A	1892.140
2R_IV_NIE_137	N/A	N/A	1893.145
2R_IV_NIE_138	N/A	N/A	1900.133
2R_IV_NIE_139	N/A	N/A	1914.119
2R_IV_NIE_140	N/A	N/A	1914.147
2R_IV_NIE_141	N/A	N/A	1916.126
2R_IV_NIE_142	N/A	N/A	1918.143
2R_IV_NIE_143	N/A	N/A	1936.096
2R_IV_NIE_144	N/A	N/A	1994.285
2R_IV_NIE_145	N/A	N/A	2016.265
2R_IV_NIE_148	N/A	N/A	2042.138
2R_IV_NIE_149	N/A	N/A	2052.184
2R_IV_NIE_150	N/A	N/A	2060.147
2R_IV_NIE_151	N/A	N/A	2064.119
2R_IV_NIE_152	N/A	N/A	2278.361
2R_IV_NIE_153	N/A	N/A	2300.340
2R_IV_NIE_154	N/A	N/A	2318.224

Table S-34: Screen [IV], T-2RT Comparison Match Any Source

Comparison of 154 non-isomeric ellagitannins (NIE's) present in strawberry leaves to those previously reported not limited to the same plant tissue. Species previously given incorrect tentative identification by closest matched molecular formula permutations have strikethroughs in this table for increased clarity.

NIE No.	Matched Fragment(s) (*m/z*)	Precursor Δ (Da)	Mass (Da)		Reported Mass (Da)	Reported Fragment(s) (*m/z*)	Ref.	E No.	Ellagitannin	Plant Source
2R_IV_NIE_1	300.999	0.002	482.072		482.070	301.00	64	E1	2,3-(S)-HHDP-glucose	*Strawberry Leaves*
2R_IV_NIE_12	481.063; 463.051; 300.999; 275.019; 300.918	0	634.081		634.081	633.073; 481.062; 463.051; 331.067; 300.999; 275.020; 300.92	73;62;64; 74;NIST	E21;E4,E5, E8;E3,E4, E5;E6,E9	Isostrictinin, Corilagin, Corilagin - CAS [23094-69-1], Galloyl-HHDP-hexose, galloyl-HHDP-glucose a-c	*Strawberry Leaves,* Strawberry Fruit, Not Strawberry
2R_IV_NIE_22	300.999	0.001	784.077		784.076	481; 301; 301.04; 275	32;72; 73;64;74	E15;E1;E20; E2;E16	Pedunculagin, Bis-(HHDP)-glucose	*Strawberry Leaves,* Strawberry Fruit, Not Strawberry
2R_IV_NIE_23	633.077; 615.065; 483.076; 313.056; 300.998	0	786.092		786.092	633.074; 615.063; 463.053; 483.078; 313.057; 300.999; 300.97	32;73;62; 64;74	E1;E22;E7, E13;E6;E18	Digalloyl-HHDP-hexose, Tellimagrandin I	*Strawberry Leaves,* Strawberry Fruit, Not Strawberry
2R_IV_NIE_39	915.053; 631.057; 300.999	-0.001	934.072		934.073	915.051; 631.058; 479.046; 461.038; 300.999	32;73;62;74	E8,E9;E1,E4; E9,E10,E12, E19;E3,E4	Galloyl-HHDP-hexose-HHDP, Vescalagin, Castalagin	Not Strawberry, Strawberry Fruit
2R_IV_NIE_41	633.073; 300.999	0.001	936.087		936.086	935.081; 783.069; 765.057; 633.073; 463.052; 300.999	72;73;62;74	E7;E5,E9,E19; E20,E21,E26; E5,E8,E12	Galloyl bis-hexahydroxydiphenoyl (HHDP)-glucose, Stachyurin, Galloyl-diHHDP-hexose, Casuarinin, Casuarictin	Not Strawberry, Strawberry Fruit
2R_IV_NIE_44	767.075; 300.999	0	938.103		938.103	937.095, 785.079, 767.074, 635.198; 465.067, 300.999	62	E22,E23	Trigalloyl-HHDP hexose	Strawberry Fruit
2R_IV_NIE_47	N/A	N/A	950.068		950		73	E7	Methyl derivative of stachyurin	Not Strawberry

2R_IV_NIE_48	781.056; 300.999	-0.001	952.082		952.083	907.085; 781.054; 605.079; 479; 425.025; 298; 273.004; 301	32;62;74; NIST	E18;E1,E2,E6,E11, E14,E15,E18;E11	Galloyl-HHDP-DHHDP-hexose, Geraniin, Geraniin - CAS[60976-49-0]	Not Strawberry, Strawberry Fruit
2R_IV_NIE_49	300.999	0	954.098		954.1	301	32;74;NIST	E16;E13	Chebulagic acid, Chebulagic acid - CAS [23094-71-5]	Not Strawberry
2R_IV_NIE_51	N/A	0	956.115		956.11		74;NIST	E14	Chebulinic acid, Chebulinic acid - CAS [18942-26-2]	Not Strawberry
2R_IV_NIE_77	N/A	0	1104.094		1104.09		73;74	E12;E7	Hippophaenin B	Not Strawberry
2R_IV_NIE_82	N/A	N/A	1118.110		1118		73	E14	Methyl derivative of hippophaenin B	Not Strawberry
2R_IV_NIE_92	N/A	N/A	1236.080		1236		73	E15	Elaeagnatin A	Not Strawberry
2R_IV_NIE_105	300.998	0.004	1416.145		1416.141	300.97	64	E7	dimeric ellagitannin 1416 Da	*Strawberry Leaves*
2R_IV_NIE_114	765.055; 300.999	0	1568.155		1568.15	765;301	32;73;74	E2;E27;E19	Oenothein B	Not Strawberry
2R_IV_NIE_119	300.998	0.003	1718.150		1718.147	300.96	64	E8,E9,E10	dimeric ellagitannin 1718 Da a-c	*Strawberry Leaves*
2R_IV_NIE_131	300.999	0	1868.144		1868.14	301	32;73;74	E12;E18;E20	Cocciferin D2	Not Strawberry
2R_IV_NIE_132	300.999	0.001	1870.159		1870.158	300.99	32;73;64;74	E4,E6;E24, E26;E17,E18; E22,E23	Sanguiin H-6, Agrimoniin, dimeric ellagitannin 1870 Da	*Strawberry Leaves,* Not Strawberry
2R_IV_NIE_146	300.998	0.003	2020.156		2020.153	300.99	64	E11,E12, E13,E14	dimeric ellagitannin 2020 Da a-d	*Strawberry Leaves*
2R_IV_NIE_147	300.999	0.003	2038.167		2038.164	300.96	64	E15, E16	dimeric ellagitannin 2038 Da a-b	*Strawberry Leaves*
					484.159	331.0674; 313.0574	62	E16	~~Digalloyl hexose~~	Strawberry Fruit
2R_IV_NIE_2	N/A	N/A	491.047							
2R_IV_NIE_3	N/A	N/A	492.185							
2R_IV_NIE_4	N/A	N/A	494.040							
2R_IV_NIE_5	N/A	N/A	510.066							

2R_IV_NIE_6	N/A	N/A	524.211							
2R_IV_NIE_7	N/A	N/A	542.295							
2R_IV_NIE_8	N/A	N/A	550.058							
2R_IV_NIE_9	N/A	N/A	580.039							
2R_IV_NIE_10	N/A	N/A	606.086							
2R_IV_NIE_11	N/A	N/A	632.066							
					636.096	465	72	E6	Trigalloyl glucose	Strawberry Fruit
					636.099	465.067; 313.056; 483.077;	62	E17	~~Trigalloyl hexose~~	Strawberry Fruit
2R_IV_NIE_13	N/A	N/A	638.075							
2R_IV_NIE_14	N/A	N/A	650.076							
					652.09		74	E10	Chebulanin	Not Strawberry
2R_IV_NIE_15	N/A	N/A	656.063							
2R_IV_NIE_16	N/A	N/A	664.091							
2R_IV_NIE_17	N/A	N/A	666.072							
2R_IV_NIE_18	N/A	N/A	672.058							
2R_IV_NIE_19	N/A	N/A	682.066							
2R_IV_NIE_20	N/A	N/A	688.054							
2R_IV_NIE_21	N/A	N/A	724.051							
					788.111	787.096; 635.088; 617.078; 465.068; 300.999; 169.013	62	E27	~~Tetragalloyl hexose~~	Strawberry Fruit
2R_IV_NIE_24	N/A	N/A	800.072							
2R_IV_NIE_25	N/A	N/A	802.087							
2R_IV_NIE_26	N/A	N/A	806.058							
2R_IV_NIE_27	N/A	N/A	816.103							
2R_IV_NIE_28	N/A	N/A	832.098							
2R_IV_NIE_29	N/A	N/A	838.084							
2R_IV_NIE_30	N/A	N/A	852.064							
2R_IV_NIE_31	N/A	N/A	874.045							

2R_IV_NIE_32	N/A	N/A	876.067						
2R_IV_NIE_33	N/A	N/A	906.115						
2R_IV_NIE_34	N/A	N/A	908.093						
2R_IV_NIE_35	N/A	N/A	918.171						
2R_IV_NIE_36	N/A	N/A	922.073						
2R_IV_NIE_37	N/A	N/A	926.105						
2R_IV_NIE_38	N/A	N/A	930.075						
2R_IV_NIE_40	N/A	N/A	935.075						
2R_IV_NIE_42	N/A	N/A	936.125						
2R_IV_NIE_43	N/A	N/A	938.096						
2R_IV_NIE_45	N/A	N/A	940.120						
				940.126	939.112; 787.098; 769.089; 617.080; 599.070; 447.059; 300.999; 169.014	62;74	E29;E15	~~Pentagalloyl hexose, PGG~~	Not Strawberry, Strawberry Fruit
2R_IV_NIE_46	N/A	N/A	942.034						
				948		73	E3	Methyl derivative of vescalagin	Not Strawberry
				948.051	929; 901; 883; 875	72	E2	Unknown Ellagitannin	Strawberry Fruit
2R_IV_NIE_50	N/A	N/A	956.054						
2R_IV_NIE_52	N/A	N/A	958.070						
2R_IV_NIE_53	N/A	N/A	964.083						
2R_IV_NIE_54	N/A	N/A	964.141						
2R_IV_NIE_55	N/A	N/A	966.099						
2R_IV_NIE_56	N/A	N/A	968.118						
2R_IV_NIE_57	N/A	N/A	974.065						
2R_IV_NIE_58	N/A	N/A	982.094						
2R_IV_NIE_59	N/A	N/A	984.071						
2R_IV_NIE_60	N/A	N/A	984.111						
2R_IV_NIE_61	N/A	N/A	988.081						

2R_IV_NIE_62	N/A	N/A	996.047						
2R_IV_NIE_63	N/A	N/A	998.126						
2R_IV_NIE_64	N/A	N/A	1004.075						
2R_IV_NIE_65	N/A	N/A	1024.042						
2R_IV_NIE_66	N/A	N/A	1026.057						
2R_IV_NIE_67	N/A	N/A	1046.025						
2R_IV_NIE_68	N/A	N/A	1056.070						
2R_IV_NIE_69	N/A	N/A	1060.105						
2R_IV_NIE_70	N/A	N/A	1064.034						
				1068		73	E8	Pterocarinin A	Not Strawberry
2R_IV_NIE_71	N/A	N/A	1082.086						
				1084.07	301	32;74;NIST	E17;E17	Punicalagin, Punicalagin - CAS [65995-63-3]	Not Strawberry
2R_IV_NIE_72	N/A	N/A	1084.126						
2R_IV_NIE_73	N/A	N/A	1086.017						
2R_IV_NIE_74	N/A	N/A	1086.084						
2R_IV_NIE_75	N/A	N/A	1098.142						
2R_IV_NIE_76	N/A	N/A	1100.122						
				1102.08	301	32;73;74	E10,E11;E10,E11;E1,E2	Vescavaloninic acid, Castavaloninic acid	Not Strawberry
2R_IV_NIE_78	N/A	N/A	1108.066						
2R_IV_NIE_79	N/A	N/A	1110.104						
2R_IV_NIE_80	N/A	N/A	1114.012						
2R_IV_NIE_81	N/A	N/A	1114.136						
2R_IV_NIE_83	N/A	N/A	1126.077						
2R_IV_NIE_84	N/A	N/A	1128.117						
2R_IV_NIE_85	N/A	N/A	1130.047						
2R_IV_NIE_86	N/A	N/A	1140.092						
2R_IV_NIE_87	N/A	N/A	1148.059						
2R_IV_NIE_88	N/A	N/A	1190.168						

2R_IV_NIE_89	N/A	N/A	1198.173						
				1206		73	E2	Catechin derivative of vescalagin	Not Strawberry
				1208		73	E6	Catechin derivative of stachyurin	Not Strawberry
2R_IV_NIE_90	N/A	N/A	1216.047						
2R_IV_NIE_91	N/A	N/A	1234.196						
2R_IV_NIE_93	N/A	N/A	1252.073						
2R_IV_NIE_94	N/A	N/A	1258.060						
2R_IV_NIE_95	N/A	N/A	1264.131						
2R_IV_NIE_96	N/A	N/A	1266.145						
2R_IV_NIE_97	N/A	N/A	1288.129						
2R_IV_NIE_98	N/A	N/A	1296.128						
2R_IV_NIE_99	N/A	N/A	1334.152						
2R_IV_NIE_100	N/A	N/A	1348.263						
2R_IV_NIE_101	N/A	N/A	1352.257						
2R_IV_NIE_102	N/A	N/A	1374.240						
				1376		73	E13	Catechin derivative of hippophaenin B	Not Strawberry
2R_IV_NIE_103	N/A	N/A	1386.075						
2R_IV_NIE_104	N/A	N/A	1404.086						
2R_IV_NIE_106	N/A	N/A	1418.102						
2R_IV_NIE_107	N/A	N/A	1434.153						
2R_IV_NIE_108	N/A	N/A	1438.124						
2R_IV_NIE_109	N/A	N/A	1448.170						
2R_IV_NIE_110	N/A	N/A	1460.102						
2R_IV_NIE_111	N/A	N/A	1482.090						
2R_IV_NIE_112	N/A	N/A	1514.233						
2R_IV_NIE_113	N/A	N/A	1566.139						

				1568.15	1415.141; 1265.137; 1103.059; 1085.076; 633.074; 935.081; 783.069; 481.061	62	E24,E25	Sanguiin h10	Strawberry Fruit
				1569.1	301	32	E13	sanguiin H10	Not Strawberry
2R_IV_NIE_115	N/A	N/A	1584.149						
2R_IV_NIE_116	N/A	N/A	1590.135						
2R_IV_NIE_117	N/A	N/A	1598.126						
2R_IV_NIE_118	N/A	N/A	1612.117						
2R_IV_NIE_120	N/A	N/A	1720.166						
2R_IV_NIE_121	N/A	N/A	1736.159						
2R_IV_NIE_122	N/A	N/A	1738.165						
2R_IV_NIE_123	N/A	N/A	1740.130						
2R_IV_NIE_124	N/A	N/A	1750.175						
2R_IV_NIE_125	N/A	N/A	1758.141						
2R_IV_NIE_126	N/A	N/A	1762.114						
2R_IV_NIE_127	N/A	N/A	1772.159						
2R_IV_NIE_128	N/A	N/A	1780.123						
2R_IV_NIE_129	N/A	N/A	1784.094						
2R_IV_NIE_130	N/A	N/A	1794.139						
				1851		73	E16	Roburin A	Not Strawberry
				1865		73	E17	Methyl derivative of roburin A	Not Strawberry
				1870.146	1869.143; 1567.152; 1265.140; 1085.076; 935.082; 783.068; 633.074; 613.049; 481.064; 301.000	62	E28	Galloyl-diHHDP-hexose dimer	Strawberry Fruit
				1873.3	301	32;73	E7;E23	Gemin A	Not Strawberry

					1875.3	301	32	E14	rugosin D	Not Strawberry
2R_IV_NIE_133	N/A	N/A	1884.140							
2R_IV_NIE_134	N/A	N/A	1886.154							
2R_IV_NIE_135	N/A	N/A	1890.125							
2R_IV_NIE_136	N/A	N/A	1892.140							
2R_IV_NIE_137	N/A	N/A	1893.145							
2R_IV_NIE_138	N/A	N/A	1900.133							
2R_IV_NIE_139	N/A	N/A	1914.119							
2R_IV_NIE_140	N/A	N/A	1914.147							
2R_IV_NIE_141	N/A	N/A	1916.126							
					1918.139	1557; 1224; 1099; 1096; 986; 967	72	E3	Unknown Ellagitannin	Strawberry Fruit
2R_IV_NIE_142	N/A	N/A	1918.143							
					1934.125	1859; 948; 947; 942; 942; 929; 921; 920	72	E4	Unknown Ellagitannin	Strawberry Fruit
2R_IV_NIE_143	N/A	N/A	1936.096							
2R_IV_NIE_144	N/A	N/A	1994.285							
2R_IV_NIE_145	N/A	N/A	2016.265							
2R_IV_NIE_148	N/A	N/A	2042.138							
2R_IV_NIE_149	N/A	N/A	2052.184							
2R_IV_NIE_150	N/A	N/A	2060.147							
2R_IV_NIE_151	N/A	N/A	2064.119							
2R_IV_NIE_152	N/A	N/A	2278.361							
2R_IV_NIE_153	N/A	N/A	2300.340							
2R_IV_NIE_154	N/A	N/A	2318.224							
					2353.6	301	32	E3	oenothein A	Not Strawberry
					2502.223	301	64	E19	trimeric ellagitannin 2502 Da	*Strawberry Leaves*
					2804.23	301	32;73;74	E5;E25;E21	Lambertianin C	Not Strawberry

					2804.229	300.99	64	E20	trimeric ellagitannin 2804 Da	*Strawberry Leaves*

References:

(1) Hukkanen, A. T.; Kokko, H. I.; Buchala, A. J.; McDougall, G. J.; Stewart, D.; Kärenlampi, S. O.; Karjalainen, R. O. *Journal of Agricultural and Food Chemistry* **2007**, *55*, 1862-1870.
